# Adamantane-Substituted Purine Nucleosides: Synthesis, Host–Guest Complexes with β-Cyclodextrin and Biological Activity

**DOI:** 10.3390/ijms232315143

**Published:** 2022-12-01

**Authors:** Jana Rudolfová, Vladimír Kryštof, Marek Nečas, Robert Vícha, Michal Rouchal

**Affiliations:** 1 Department of Chemistry, Faculty of Technology, Tomas Bata University in Zlín, Vavrečkova 5669, 760 01 Zlín, Czech Republic; 2 Department of Experimental Biology, Palacký University, Šlechtitelů 27, 783 71 Olomouc, Czech Republic; 3 Department of Chemistry, Faculty of Science, Masaryk University, Kotlářská 2, 602 00 Brno, Czech Republic

**Keywords:** adamantane, purine, nucleoside, glycosylation, β-cyclodextrin, antiproliferative activity

## Abstract

Purine nucleosides represent an interesting group of nitrogen heterocycles, showing a wide range of biological effects. In this study, we designed and synthesized a series of 6,9-disubstituted and 2,6,9-trisubstituted purine ribonucleosides via consecutive nucleophilic aromatic substitution, glycosylation, and deprotection of the ribofuranose unit. We prepared eight new purine nucleosides bearing unique adamantylated aromatic amines at position 6. Additionally, the ability of the synthesized purine nucleosides to form stable host–guest complexes with β-cyclodextrin (β-CD) was confirmed using nuclear magnetic resonance (NMR) and mass spectrometry (ESI-MS) experiments. The in vitro antiproliferative activity of purine nucleosides and their equimolar mixtures with β-CD was tested against two types of human tumor cell line. Six adamantane-based purine nucleosides showed an antiproliferative activity in the micromolar range. Moreover, their effect was only slightly suppressed by the presence of β-CD, which was probably due to the competitive binding of the corresponding purine nucleoside inside the β-CD cavity.

## 1. Introduction

Purine nucleosides represent one of the most widespread groups of nitrogen heterocycles in nature. They have been developed as analogues to their physiological counterparts, endogenous nucleosides participating in biological processes such as DNA or RNA synthesis, metabolism, cell signaling, and enzyme regulation [1]. A wide range of significant biological effects have recently been described for synthetically prepared purine nucleosides or their bioisosteres. For example, they can act as adenosine receptor ligands [2], plant growth regulators [3], or antiviral [3,4,5,6], antitumor [5,6,7,8], and antiprotozoal agents [9,10,11,12]. From a synthetic point of view, purine nucleosides represent an interesting class of compounds with structural diversity and nearly unrestricted possibilities for modifying the purine ring, especially at positions C2, C6, and N9 [13,14]. 

**Figure 1:**
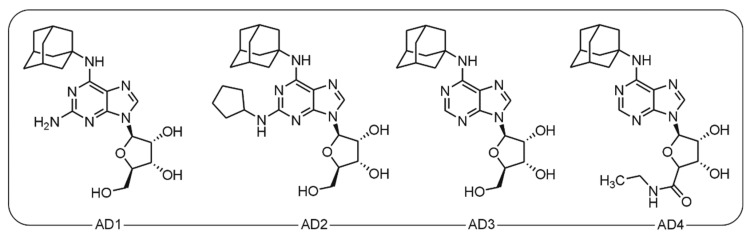
Previously published adamantane-based purine nucleosides.

Adamantane (systematically tricyclo[3.3.1.1^3,7^]decane) is a member of a group of compounds collectively referred to as cage hydrocarbons. Consisting of three cyclohexane rings in an almost ideal chair conformation, adamantane is a highly lipophilic motif introduced into the known pharmacophores to obtain biologically active compounds with a better pharmacological profile. For example, Papanastasiou and co-workers published a study dealing with the synthesis and biological evaluation of novel nifurtimox-like derivatives with the 5-nitrofuran-2-yl pharmacophore. The introduction of the adamantane motif led to higher antitrypanosomal activity and better selectivity of the new derivatives in comparison to the parent compound [15]. Structural modifications can also be carried out on drugs already containing an adamantane moiety. For instance, CMD-05, a novel orally available vildagliptin-derived dipeptidyl peptidase IV inhibitor shows a similar in vivo activity and has a much longer half-life and lower cytotoxicity than vildagliptin [16]. Notably, potential adamantane-based drugs show a wide range of interesting biological activities, such as antitumor [17,18,19], antiviral [20,21], antitrypanosomal [15,22,23], and tuberculostatic activities [22,24,25]. They can also act as cannabinoid receptor ligands [26,27,28] and adenosine receptor agonists [29,30]. Interestingly, only a few purine nucleosides bearing an adamantane moiety have been reported to date. It is possible to mention the compounds AD1–D4 (Figure 1) that were studied for their potency in displaying an antitrypanosomal activity [23] or affinity to adenosine receptors [29,30].

On the other hand, introducing an adamantane motif into the structure of the considered biologically-active substances can lead to a slight decrease in the solubility of the final compound in a water environment. This limitation can be overcome using one of the most interesting properties of the adamantane moiety, i.e., its ability to form relatively stable supramolecular host–guest complexes with cyclodextrins (CDs) [31,32]. CDs represent non-toxic, biodegradable, and water-soluble cyclic oligosaccharides, originating via bacterial degradation of starch. Natural CDs, consisting of six to eight glucose subunits linked by α-(1,4)-glycosidic bonds, are generally known as α-, β-, and γ-CD, respectively. CDs resemble a truncated cone shape with a hydrophilic exterior of their cavity (due to the presence of primary and secondary hydroxyl groups) and a hydrophobic cavity interior, in which a lipophilic guest molecule (or part of it) can be included via non-covalent interactions. Several types of modified CDs, prepared by the transformation of hydroxyl groups with different functional motifs, have been synthesized, with the aim of improving the water solubility of natural CDs [33]. The influence of the host–guest complex formation between potential drug candidates and CDs (natural and/or modified), for example, on water solubility, biological activity, bioavailability, and/or stability, has been extensively studied [31,32,34,35,36,37].

**Figure 2:**
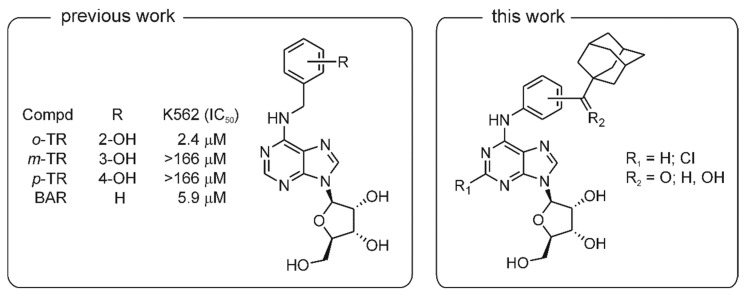
Purine nucleosides considered in this work and structurally related derivatives with their antiproliferative activity towards the K562 cell line. IC_50_ values were taken from a previously published paper [11].

Inspired by a previously published study dealing with the antiproliferative activity of natural cytokinin ribosides [11], such as *ortho*-, *meta*- and *para*-topolin ribosides (hereinafter discussed as *o*-TR, *m*-TR and *p*-TR, respectively) and N6-benzyladenosine (BAR), we synthesized a novel series of purine ribonucleosides substituted at C6 with aromatic amines bearing an adamantane moiety (Figure 2). Moreover, the ability of the prepared compounds to form stable host–guest complexes with β-CD was studied using nuclear magnetic resonance (NMR) and electrospray ionization mass spectrometry (ESI-MS). The new purine nucleosides and their equimolar mixtures with β-CD were assayed for their antiproliferative activity.

## 2. Results and Discussion

### 2.1. Chemistry

**Scheme 1:**
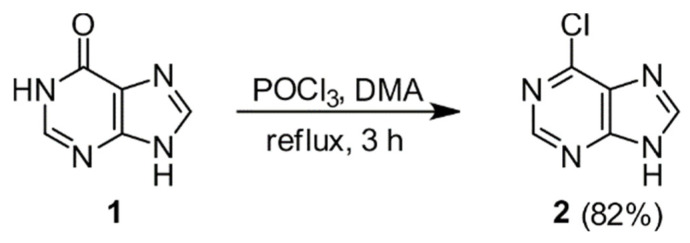
Synthesis of 6-chloro-9*H*-purine (**2**).

For the synthesis of a novel series of adamantane-substituted purine nucleosides, two starting purine derivatives, namely 6-chloro-9*H*-purine (**2**) and 2,6-dichloro-9*H*-purine (**3**), were used. Compound **2** was prepared using a slightly modified literature procedure [38] through the reaction of commercially available hypoxanthine (**1**) with phosphoryl oxychloride in dimethylaniline (Scheme 1). Compound **3** was obtained from commercial sources and used without further purification.

**Scheme 2:**
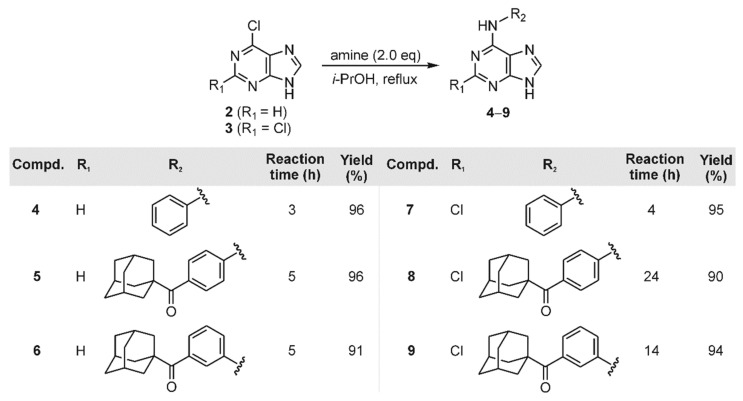
Introduction of aromatic amines to the C6 position of compounds **2** and **3**.

The introduction of the previously prepared adamantylated aromatic amines [39] or aniline (as a model substituent) to position C6 of purines **2** and **3**, respectively, led to the formation of compounds **4**–**9** (Scheme 2). The nucleophilic aromatic substitution of chlorine at C6 was carried out using two molar excess of the corresponding amine in refluxing propan-2-ol [40]. Purines **4**–**9** were isolated, after the purification using column chromatography and/or crystallization, in good purity and high yields (90–96%).

**Scheme 3:**
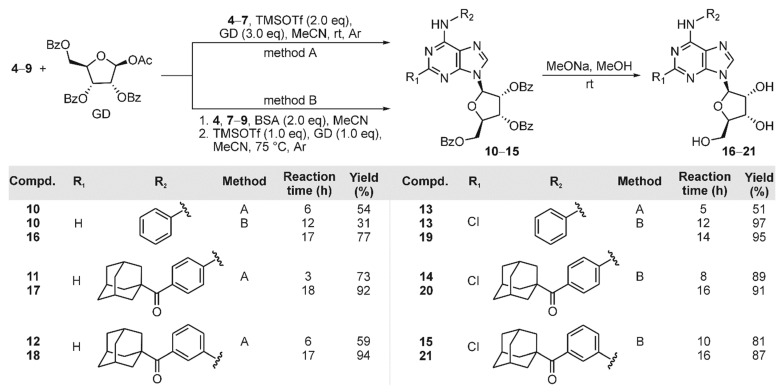
Glycosylation and deprotection of purine derivatives.

According to our synthetic strategy, we subsequently performed glycosylation of purines **4**–**9** at position N9 of the purine ring with commercially available 1-*O*-acetyl-2,3,5-*O*-benzoyl ribofuranose. To optimize this step, we carried out a series of reactions under different conditions (catalysator, solvent, temperature) [41,42,43] with model compounds **4** and **7**. During the optimization, we achieved the best results using modified Hilbert–Johnson glycosylation [44] under catalysis with trimethylsilyl trifluoromethanesulfonate (TMSOTf) in CH_3_CN at room temperature (hereinafter referred to as “method A”), and two-step Vörbruggen glycosylation [45] using *N*,*O*-bis(trimethylsilyl)acetamide (BSA) followed by glycosylation with TMSOTf in CH_3_CN at 75 °C (hereinafter referred to as “method B”). Interestingly, we obtained the desired compounds in different yields, depending on the substitution of the starting purine at the C2 position. In the case of purine **4**, bearing a hydrogen atom at C2, compound **10** was isolated, yielding about 54% using method A, while method B provided the same compound, yielding 31%. When these conditions were applied to purine **7** (with chlorine at C2), we obtained the desired compound **13**, yielding 51% (method A) and 97% (method B), respectively. According to these results we decided to prepare glycosylated purines bearing a 1-adamantyl moiety depending on the substitution of the starting purines at the C2 position. While compounds **11** and **12** were synthesized using method A, purines **14** and **15** were prepared using method B, as depicted in Scheme 3.

Deprotection of benzoyl groups from purine nucleosides **10**–**15** was carried out under the Zemplén conditions [46] using sodium methoxide in MeOH at room temperature (Scheme 3). The desired nucleosides **16**–**21** were prepared in good purity, with yields of about 77–95%.

**Scheme 4:**
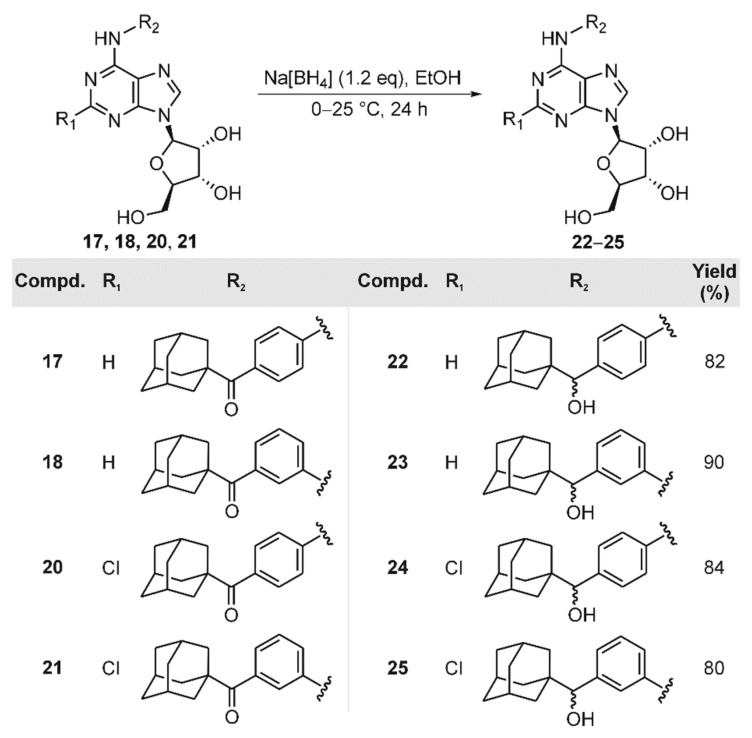
Reduction of purines **17**, **18**, **20**, and **21** with Na[BH_4_].

Finally, reduction of the oxo group linking the 1-adamantyl moiety and the benzene ring of the purine nucleosides **17**, **18**, **20**, and **21** was performed. The reaction was carried out using a slight excess of sodium borohydride in EtOH and provided the required alcohols **22**–**25** in yields of 80–90% (Scheme 4). It should be noted that we observed only one set of signals in the ^1^H-NMR spectra of purines **22**–**25** (see Supplementary Materials, Figures S38, S40, S42 and S44, respectively), so we assume that either the reaction proceeded diastereoselectively or that the diastereoisomers cannot be distinguished using NMR.

The structure of all the prepared compounds was proposed based on the results obtained by infrared spectroscopy, electron and/or electrospray ionization mass spectrometry, and ^1^H- and ^13^C-nuclear magnetic resonance (see Experimental Section 3.2, Section 3.3, Section 3.4, Section 3.5 and Section 3.6). Moreover, the molecular structure of purine **13** was proven using X-ray diffraction analysis, confirming the origination of β-nucleoside, as shown in Figure 3 (for all the geometric parameters, see Supplementary Materials, Figure S46 and Table S1). ^1^H- and ^13^C-NMR of the prepared compounds are given in the Supplementary Materials (Figures S1–S45).

### 2.2. Host–Guest Complexes with β-CD

#### 2.2.1. ESI-MS Analyses

The ability of purine nucleosides **16**–**25** to form host–guest complexes with β-CD in the gas phase was studied in both positive and negative-ion polarity modes. The equimolar mixture of the corresponding purine nucleoside and β-CD was prepared immediately before each experiment.

In the first-order mass spectra obtained in the positive-ion polarity mode, ions originating from the macrocycle, the corresponding purine nucleoside and their supramolecular complex were observed. The guest molecule was represented by singly charged ions, which we assigned as a protonated molecule [M + H^+^]^+^, and sodium [M + Na^+^]^+^ and potassium [M + K^+^]^+^ adducts of the molecule. These ions were accompanied by a singly charged ion approximately twice as high, assigned as the sodium adduct of the dimer [2·M + Na^+^]^+^. Two singly charged ions with *m*/*z* at 1157 and 1173, respectively, were assigned as sodium and potassium adducts of the β-CD ([β-CD + Na^+^]^+^ and [β-CD + K^+^]^+^), respectively. The value of the last ion observed in the spectrum corresponds to the sodium adduct of the host–guest complex with 1:1 stoichiometry ([M@β-CD + Na^+^]^+^).

**Figure 4:**
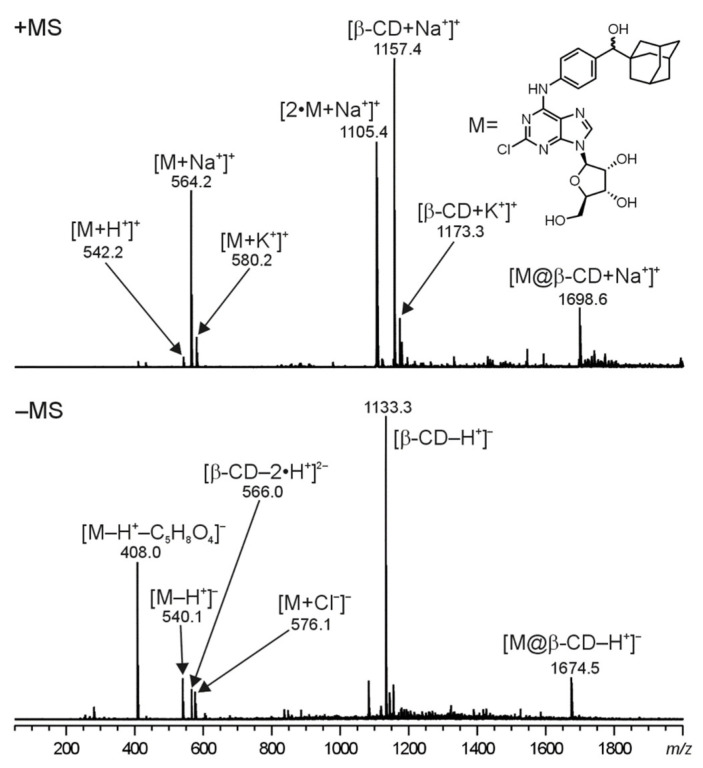
Positive and negative-ion ESI mass spectra of the equimolar mixture of **24**·β-CD. The assignments for the observed ions are shown in the brackets.

In the negative-ion polarity mode, singly charged ions assigned as a deprotonated molecule [M–H^+^]^−^ and the chloride adduct of the molecule [M + Cl^−^]^−^ were observed. The last ion related to the examined compound was assigned as a deprotonated molecule lacking the ribose unit [M–H^+^–C_5_H_8_O_4_]^−^. The structure of this ion was proposed according to the tandem mass spectrometry (MS/MS) experiment of the deprotonated molecule under collision-induced dissociation conditions. According to the obtained results, we can suggest that the last ion originated in the ion source as a product of the in-source fragmentation. Two ions, singly (at *m*/*z* 1133) and doubly charged (at *m*/*z* 566), were assigned as deprotonated molecules of the macrocycle ([β-CD–H^+^]^−^ and [β-CD–2·H^+^]^2−^, respectively). Finally, we observed the ion at *m*/*z* corresponding to the 1:1 host–guest complex assigned as [M@β-CD–H^+^]^−^. Unfortunately, all attempts to isolate and subsequently fragment (MS/MS) the ions corresponding to the host–guest complex were unsuccessful. Illustrative positive and negative-ion mass spectra of the equimolar mixture of purine **24** with β-CD are shown in Figure 4.

#### 2.2.2. Nuclear Magnetic Resonance Analyses

**Figure 5:**
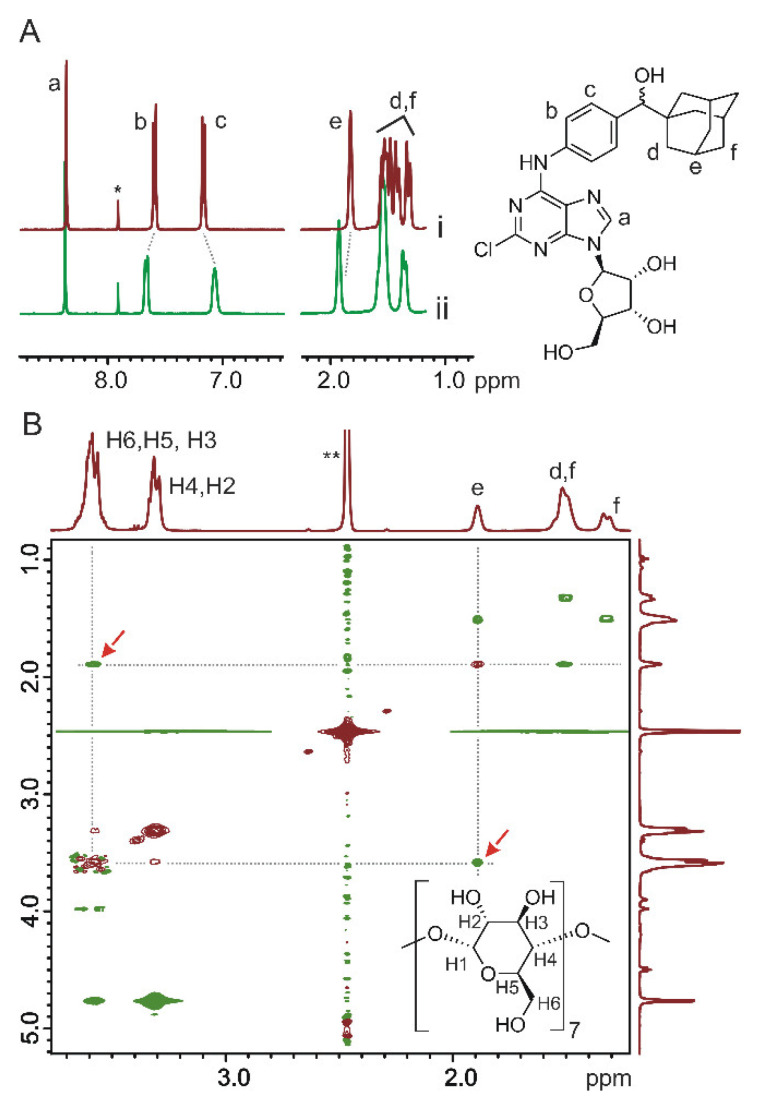
(**A**) Portions of the ^1^H-NMR spectra of the single compound **24** (i) and after the addition of 1 equiv. of β-CD (ii). (**B**) A ROESY spectrum of the latter mixture. All spectra were recorded in a mixture of DMSO-*d*_6_/D_2_O, 2/1, *v*/*v* at 303 K at 400 MHz. Signals of residual CHCl_3_ and DMSO-*d*_5_ are labelled with * and **, respectively.

Consequently, we employed NMR spectroscopy to gain an independent confirmation of the host–guest complex formation. We selected compound **24** as a representative of purine glycosides and examined its mixture with β-CD. These experiments were carried out in a DMSO-*d*_6_/D_2_O (2/1, *v*/*v*) mixture, due to insufficient solubility of the glycosides in pure water. Initially, we recorded two ^1^H-NMR spectra for a single compound **24** and its mixture with one equivalent of β-CD, respectively. Portions of these spectra are shown in Figure 5A. We observed clear complexation-induced shifts (CIS) of 0.10 ppm, 0.07, and −0.10 ppm for the adamantane bridgehead H(e) atoms and aromatic H(b) and H(c) atoms, respectively. The downfield shift of adamantane signals is well recognized as an indication of the inclusion of the adamantane moiety into the β-CD cavity [47,48]. In addition, we performed a 2D ^1^H-^1^H ROESY experiment on the equimolar mixture of **24** and β-CD. As can be seen in Figure 5B, this experiment revealed the spatial proximity of the adamantane H(e)-atoms and cyclodextrin H3 and H5 atoms, which decorate the interior of the β-CD cavity. The corresponding cross-peaks are indicated by red arrows in Figure 5B. These results imply that a significant portion of compound **24** was present in the form of an inclusion complex with the adamantane moiety immersed in the β-CD cavity in a DMSO/water mixture. However, this conclusion can be extended to the water environment, since the hydrophobic effect, where strength increases with the polarity of the medium, is the most important stabilization force in the case of CD complexes.

### 2.3. Antiproliferative Activity

The antiproliferative activity of purine nucleosides **16**–**25** was tested against two types of human tumor cell lines, namely MV4;11 (acute myelogenous leukemia) and K562 (chronic myelogenous leukemia). The in vitro activity was studied with both single purine nucleosides and their equimolar mixtures with β-CD (Table 1). Purine nucleosides **16** and **19** (with a phenyl ring at C6) were used as reference compounds. They showed no effect, both singly and with β-CD mixtures, against the tested cell lines for concentrations up to 25 μM. On the other hand, most of the adamantylated purine nucleosides (except compounds **17** and **25**) displayed antiproliferative activity against the MV4;11 cell line in the micromolar range (IC_50_ = 12.3–23.8 μM). The highest efficacy was shown by compounds with chlorine at C2 and a para-substituted aromatic ring at C6, namely purine **20** (with the oxo group between 1-adamantyl and the benzene ring) and purine **24** (with the hydroxy group between 1-adamantyl and the benzene ring). A similar antiproliferative activity was observed for purine **23** with a hydrogen atom at C2 and a hydroxy group between 1-adamantyl and benzene ring. Purine nucleosides **18**, **21**, and **22** showed effects against MV4;11 in concentrations higher than 20 μM.

Unfortunately, most of the prepared purine nucleosides and their mixtures with β-CD showed no antiproliferative activity against the K562 cell line for concentrations up to 25 μM. On the other hand, purine nucleosides **20** and **24** inhibited cell proliferation of this cell line in the micromolar range (IC_50_ = 21.4 and 23.4 μM, respectively). Similar results were previously published for a series of structurally related compounds (see Figure 2), where *meta*- and *para*-topolin-β-d-ribofuranosides showed no antiproliferative activity against K562. In comparison, the *ortho*-substituted derivative and N6-benzyladenosine (without hydroxyl group at the phenyl ring) displayed effects in the micromolar range [11].

Interestingly, purine nucleosides **18** and **20**–**24**, inhibiting the cell proliferation of one or both tested cell lines, also showed activity as equimolar mixtures with β-CD. This fact implied the presence of relatively stable supramolecular host–guest complexes of the prepared purine nucleosides with β-CD, as suggested by the NMR and ESI-MS analyses. The slight decrease in the antiproliferative activity of purine·β-CD equimolar mixtures could be explained as the result of competition for the biological target with β-CD for a corresponding purine nucleoside.

## 3. Materials and Methods

### 3.1. General Data

All starting compounds, reagents, and solvents were purchased from commercial sources in analytical quality and were used without further purification. Adamantylated aromatic amines were prepared following previously published procedures [39,49,50]. Melting points were measured on a Kofler block and were not corrected. Elemental analyses (C, H, N, S) were performed with a Thermo Fisher Scientific Flash EA 1112. Retention times were determined using thin-layer chromatography (TLC) plates (Alugram Sil G/UV) from Machrey-Nagel. Several types of mobile phases were used: CHCl_3_/EtOAc, 7/3, *v*/*v* (system a), CHCl_3_/MeOH, 5/1, *v*/*v* (system b), CHCl_3_/MeOH, 8/1, *v*/*v* (system c), CHCl_3_/MeOH, 12/1, *v*/*v* (system d), CHCl_3_/EtOH, 19/1, *v*/*v* (system e), CHCl_3_/EtOH, 4/1, *v*/*v* (system f), PE/EtOAc, 8/1, *v*/*v* (system h), and EtOAc/MeOH, 9/1, *v*/*v* (system i). The NMR spectra were recorded on a Bruker Avance-500 spectrometer at 500.13 MHz (^1^H) and 125.77 MHz (^13^C), or a JEOL ECZ400R spectrometer at 399.78 MHz (^1^H) and 100.95 MHz (^13^C). ^1^H- and ^13^C-NMR chemical shifts were referenced to the signal of the solvent (^1^H: δ(residual CHCl_3_) = 7.27 ppm and δ(residual DMSO-*d*_5_) = 2.5 ppm; ^13^C: δ(CDCl_3_) = 77.23 ppm and δ(DMSO-*d*_6_) = 39.51 ppm). The ROESY experiments were carried out with a spin-lock time of 600 ms. The IR spectra were recorded in a KBr disc with a Nicolet Avatar-380 spectrophotometer. GC-EI-MS analyses were run on a Shimadzu QP-2010 instrument using a Supelco SLB-5ms (30 m, 0.25 mm) column. Helium was employed as a carrier gas in constant linear flow mode (38 cm s^−1^): 100 °C/7 min, 25 °C/min to 250 °C, and held for the required amount of time. Electrospray mass spectra (ESI–MS) were recorded using an amaZon X ion-trap mass spectrometer (Bruker Daltonics, Bremen, Germany) equipped with an electrospray ionization source. All experiments were conducted in both positive- and negative-ion polarity modes. The instrumental conditions used to measure the single purine nucleosides and their mixtures with the host molecules were different; therefore, they are described separately. *Single guests*: Individual samples (with concentrations of 0.5 μg cm^−3^) were infused into the ESI source in MeOH/H_2_O (1/1, *v*/*v*) solutions using a syringe pump with a constant flow rate of 3 μL min^−1^. The other instrumental conditions were as follows: an electrospray voltage of ±4.2 kV, a capillary exit voltage of ±140 V, a drying gas temperature of 220 °C, a drying gas flow rate of 6.0 dm^3^ min^−1^, and a nebulizer pressure of 55.16 kPa. *Host–guest complexes*: An aqueous solution of the guest (12.5 μM) and an equimolar amount of the host were infused into the ESI source at a constant flow rate of 3 μL min^−1^. The other instrumental conditions were as follows: an electrospray voltage of ±4.0 kV, a capillary exit voltage of 40 V up to −100 V, a drying gas temperature of 300 °C, a drying gas flow rate of 6.0 dm^3^ min^−1^, and a nebulizer pressure of 206.84 kPa. Nitrogen was used as both the nebulizing and drying gas for all of the experiments. Tandem mass spectra were collected using CID, with He as the collision gas, after the isolation of the required ions. Diffraction data were collected on a Rigaku MicroMax-007 HF rotating anode four-circle diffractometer using Mo *Kα* radiation at 120 K. The structure was solved using direct methods and refined using full-matrix least-squares methods on *F*^2^ using the SHELXTL software package [51]. All non-hydrogen atoms were refined anisotropically, and hydrogen atoms were refined as riding on their carrier atoms. Crystal data and refinement parameters are gathered in Table S1 (see Supplementary Materials). The supplementary crystallographic data can be obtained free of charge from The Cambridge Crystallographic Data Centre via www.ccdc.cam.ac.uk/data_request/cif (accessed on 12 November 2022) (reference code: 2219330).

### 3.2. Synthesis of 6-Chloro-9H-purine (**2**)

Compound **2** was prepared following a modified literature procedure [38]. Into a well-stirred mixture of hypoxanthine (**1**) (200 mg, 1.47 mmol) in *N*,*N*-dimethylaniline (3 cm^3^), phosphoryl oxychloride (15 cm^3^) was added dropwise at 0 °C. After this, the reaction mixture was refluxed under an inert atmosphere till the TLC indicated the consumption of all starting material (ca 3 h). During this time, the pale green color of the solution changed to dark red. The reaction mixture was cooled down and the liquid was evaporated in a vacuum. The obtained dark green oil was poured onto crushed ice (ca 80 cm^3^) and the mixture was stirred. After the dissolution of the ice, the mixture was neutralized using concentrated ammonia (9 cm^3^), the water layer was extracted with CHCl_3_ (3 × 5 cm^3^) and evaporated in a vacuum. The obtained yellow crystalline solid was repeatedly washed with hot CH_3_CN (to remove the inorganic co-products), and combined CH_3_CN portions were dried over sodium sulfate and evaporated in a vacuum. Purification of the crude product using column chromatography (system b) provided the desired compound **2**.

6-Chloro-9*H*-purine (2)

Pale yellow crystalline powder, yield 187 mg (82%), mp 212–214 °C, R_f_ = 0.42 (system b). ^1^H NMR (DMSO-*d*_6_, 500 MHz): *δ* 8.44 (s, 1H, C^2^H), 8.63 (d, 1H, *J* = 6.51 Hz, C^8^H) ppm. ^13^C NMR (DMSO-*d*_6_, 125 MHz): *δ* 129.3 (C), 145.3 (C), 147.9 (C), 151.8 (C), 153.8 (C) ppm (NMR match the literature [52]). IR (KBr): 3064 (m), 2934 (m), 2804 (m), 1604 (m), 1573 (s), 1549 (m), 1445 (m), 1390 (s), 1325 (s), 1285 (s), 1234 (m), 988 (m), 850 (m), 693 (m), 604 (m) cm^−1^. GC-EI-MS *m*/*z* (%): 156 (M^+^(^37^Cl), 30), 155 (7), 154 (M^+^(^35^Cl), 97), 147 (6), 135 (11), 120 (7), 119 (100), 99 (13), 92 (36), 85 (10), 74 (7), 73 (28), 67 (8), 66 (7), 65 (29), 64 (11), 60 (19), 52 (13), 51 (6), 46 (13), 45 (40), 44 (21), 43 (23), 42 (6), 40 (6). ESI-MS (pos.) *m*/*z* (%): 193.1 [M(^35^Cl) + K^+^]^+^ (24), 177.1 [M(^35^Cl) + Na^+^]^+^ (19), 155.1 [M(^35^Cl) + H^+^]^+^ (100). ESI-MS (neg.) *m*/*z* (%): 153.0 [M(^35^Cl) − H^+^]^−^ (100). Anal. Cald for C_5_H_3_ClN_4_: C 38.86; H 1.96; N 36.25. Found: C 38.52; H 2.14; N 36.38.

### 3.3. General Procedure for the Preparation of 6-‘Amino’-9H-purine Derivatives **4**–**9**

Compounds **4**–**9** were prepared following a slightly modified literature procedure [40]. The starting purine **2** (1.30–1.75 mmol) or **3** (0.32–1.06 mmol) were dissolved in propan-2-ol (10 cm^3^), the corresponding aromatic amine (2.0 equiv.) was added into this solution, and the reaction mixture was refluxed. After 15–45 minutes of reflux, the formation of a slightly yellow precipitation was observed. The reaction mixture was refluxed till the TLC indicated the consumption of all starting material (3–24 h). After this, the precipitation was filtrated with suction and washed with cold propan-2-ol several times. Purification of the crude product using column chromatography (system c or d) and/or crystallization resulted in the desired products.

*N*-phenyl-9*H*-purin-6-amine (4)

Prepared from compound **2** (202 mg, 1.30 mmol) and aniline (2.60 mmol). Purified using crystallization from MeOH/CHCl_3_. Pale yellow crystalline powder, yield 265 mg (96%), mp 288–290 °C, R_f_ = 0.41 (system c). ^1^H NMR (DMSO-*d*_6_, 500 MHz): *δ* 7.22 (t, 1H, *J* = 7.25 Hz, Ph), 7.45 (t, 2H, *J* = 7.35 Hz, Ph), 7.92 (d, 2H, *J* = 7.67 Hz, Ph), 8.75 (s, 1H, C^2^H), 8.83 (d, 1H, C^8^H), 11.65 (s, 1H, C^6^N**H**Ph) ppm. ^13^C NMR (DMSO-*d*_6_, 125 MHz): *δ* 121.3 (CH(Ph)), 124.8 (CH(Ph)), 128.9 (CH(Ph)), 137.7 (C(Ph)), 143.3 (C), 148.4 (C), 149.6 (C) ppm. IR (KBr): 3104 (s), 2976 (s), 2613 (m), 1652 (s), 1617 (s), 1594 (s), 1500 (s), 1440 (s), 1390 (s), 1216 (m), 908 (w), 754 (m), 686 (m), 615 (m), 590 (m) cm^−1^. GC-EI-MS *m*/*z* (%): 212 (7), 211 (M^+^, 54), 210 (100), 156 (8), 129 (7), 105 (6), 104 (7), 103 (8), 93 (7), 92 (17), 78 (5), 77 (33), 76 (5), 66 (11), 65 (18), 53 (6), 52 (5), 51 (22), 50 (6), 44 (17), 43 (6). ESI-MS (pos.) *m*/*z* (%): 212.2 [M + H^+^]^+^ (100). ESI-MS (neg.) *m*/*z* (%): 210.1 [M − H^+^]^−^ (100). Anal. Cald for C_11_H_9_N_5_: C 62.55; H 4.29; N 33.16. Found: C 62.57; H 4.11; N 33.06.

(1-Adamantyl){4-[(9*H*-purin-6-yl)-amino]phenyl}methanone (**5**)

Prepared from compound **2** (268 mg, 1.73 mmol) and (1-adamantyl)(4-aminophenyl)methanone (3.46 mmol). Purified using column chromatography (system c). Pale yellow crystalline powder, yield 624 mg (96%), mp 317–318 °C, R_f_ = 0.50 (system c). ^1^H NMR (DMSO-*d*_6_, 500 MHz): *δ* 1.72 (m, 6H, CH_2_(Ad)), 1.97 (m, 6H, CH_2_(Ad)), 2.02 (m, 3H, CH(Ad)), 7.78 (d, 2H, *J* = 7.35 Hz, Ph), 8.04 (d, 2H, *J* = 7.35 Hz, Ph), 8.77 (s, 1H, C^2^H), 8.88 (s, 1H, C^8^H), 11.65 (s, 1H, C^6^N**H**Ph) ppm. ^13^C NMR (DMSO-*d*_6_, 125 MHz): *δ* 27.6 (CH_2_(Ad)), 36.0 (CH(Ad)), 38.6 (CH_2_(Ad)), 46.1 (C(Ad)), 113.2 (CH(Ph)), 119.9 (CH(Ph)), 128.8 (CH(Ph)), 133.7 (C(Ph)), 140.4 (C), 143.1 (C), 149.0 (C), 149.3 (C), 149.5 (C), 206.7 (Ph**C**OAd) ppm. IR (KBr): 2904 (s), 2850 (m), 1628 (s), 1589 (s), 1533 (m), 1479 (s), 1411 (m), 1358 (m), 1323 (m), 1273 (m), 1238 (s), 1174 (m), 929 (w), 750 (w), 644 (w) cm^−1^. ESI-MS (pos.) *m*/*z* (%): 747.2 [2·M + H^+^]^+^ (5), 412.2 [M + K^+^]^+^ (4), 396.3 [M + Na^+^]^+^ (9), 374.3 [M + H^+^]^+^ (100). ESI-MS (neg.) *m*/*z* (%): 781.3 [2·M + Cl^−^]^−^ (14), 745.3 [2·M − H^+^]^−^ (7), 408.2 [M + Cl^−^]^−^ (20), 372.2 [M − H^+^]^−^ (100). Anal. Cald for C_22_H_23_N_5_O: C 70.76; H 6.21; N 18.75. Found: C 70.42; H 5.93; N 18.86.

(1-Adamantyl){3-[(9*H*-purin-6-yl)-amino]phenyl}methanone (**6**)

Prepared from compound **2** (271 mg, 1.75 mmol) and (1-adamantyl)(3-aminophenyl)methanone (3.50 mmol). Purified using column chromatography (system c). Pale yellow crystalline powder, yield 596 mg (91%), mp 309–315 °C, R_f_ = 0.47 (system c). ^1^H NMR (DMSO-*d*_6_, 500 MHz): *δ* 1.72 (m, 6H, CH_2_(Ad)), 1.97 (m, 6H, CH_2_(Ad)), 2.03 (m, 3H, CH(Ad)), 7.36 (d, 1H, *J* = 6.45 Hz, Ph), 7.50 (t, 1H, *J* = 6.35 Hz, Ph), 8,10 (d, 1H, *J* = 7.05 Hz, Ph), 8.27 (s, 1H, Ph), 8.73 (s, 1H, C^2^H), 8.81 (s, 1H, C^8^H), 11.62 (s, 1H, C^6^N**H**Ph) ppm. ^13^C NMR (DMSO-*d*_6_, 125 MHz): *δ* 27.5 (CH_2_(Ad)), 35.9 (CH(Ad)), 38.5 (CH_2_(Ad)), 46.2 (C(Ad)), 113.3 (CH(Ph)), 119.4 (CH(Ph)), 122.6 (CH(Ph)), 122.9 (CH(Ph)), 128.8 (CH(Ph)), 137.8 (C(Ph)), 139.1 (C), 143.0 (C), 148.9 (C), 149.1 (C), 149.8 (C), 208.0 (Ph**C**OAd) ppm. IR (KBr): 2906 (s), 2852 (m), 1666 (s), 1574 (s), 1496 (s), 1433 (s), 1387 (m), 1346 (m), 1269 (m), 1250 (m), 1211 (m), 1128 (w), 997 (w), 784 (w), 611 (w) cm^−1^. ESI-MS (pos.) *m*/*z* (%): 747.3 [2·M + H^+^]^+^ (5), 412.2 [M + K^+^]^+^ (5), 396.3 [M + Na^+^]^+^ (8), 374.3 [M + H^+^]^+^ (100). ESI-MS (neg.) *m*/*z* (%): 781.3 [2·M + Cl^−^]^−^ (14), 745.3 [2·M − H^+^]^−^ (9), 408.2 [M + Cl^−^]^−^ (31), 372.2 [M − H^+^]^−^ (100). Anal. Cald for C_22_H_23_N_5_O: C 70.76; H 6.21; N 18.75. Found: C 70.94; H 6.39; N 18.55.

*N*-phenyl-2-chloro-9*H*-purin-6-amine (**7**)

Prepared from compound **3** (200 mg, 1.06 mmol) and aniline (2.12 mmol). Purified using crystallization from CHCl_3_. Pale yellow crystalline powder, yield 247 mg (95%), mp 322–329 °C, R_f_ = 0.52 (system c). ^1^H NMR (DMSO-*d*_6_, 500 MHz): *δ* 7.08 (t, 1H, *J* = 7.35 Hz, Ph), 7.36 (t, 2H, *J* = 7.45 Hz, Ph), 7.84 (d, 2H, *J* = 7.80 Hz, Ph), 8.29 (d, 1H, C^8^H), 10.12 (s, 1H, C^6^N**H**Ph), 13.25 (s, 1H, N^9^H) ppm. ^13^C NMR (DMSO-*d*_6_, 125 MHz): *δ* 121.5 (CH(Ph)), 123.8 (CH(Ph)), 129.0 (CH(Ph)), 139.4 (C(Ph)), 141.5 (C), 152.2 (C), 152.6 (C) ppm. IR (KBr): 3040 (bm), 2782 (bm), 1634 (s), 1561 (s), 1496 (s), 1434 (s), 1254 (s), 1177 (m), 1102 (m), 960 (m), 938 (m), 730 (m), 684 (m), 611 (m), 557 (m) cm^−1^. ESI-MS (pos.) *m*/*z* (%): 268.0 [M(^35^Cl) + Na^+^]^+^ (69), 246.0 [M(^35^Cl) + H^+^]^+^ (100). ESI-MS (neg.) *m*/*z* (%): 243.8 [M(^35^Cl) − H^+^]^−^ (100). Anal. Cald for C_11_H_8_ClN_5_: C 53.78; H 3.28; N 28.51. Found: C 53.68; H 3.26; N 28.40. Spectral data match the literature [53].

(1-Adamantyl){4-[(2-chloro-9*H*-purin-6-yl)-amino]phenyl}methanone (**8**)

Prepared from compound **3** (70 mg, 0.37 mmol) and (1-adamantyl)(4-aminophenyl)methanone (0.74 mmol). Purified using column chromatography (system d) and crystallization from MeOH. Colorless crystalline powder, yield 136 mg (90%), mp 292–298 °C, R_f_ = 0.30 (system d). ^1^H NMR (DMSO-*d*_6_, 500 MHz): *δ* 1.70–1.76 (m, 6H, CH_2_(Ad)), 1.98 (m, 6H, CH_2_(Ad)), 2.03 (m, 3H, CH(Ad)), 7.75 (d, 2H, *J* = 6.45 Hz, Ph), 7.98 (d, 2H, *J* = 6.45 Hz, Ph), 8.33 (s, 1H, C^8^H), 10.46 (s, 1H, C^6^N**H**Ph), 13.38 (s, 1H, N^9^H) ppm. ^13^C NMR (DMSO-*d*_6_, 125 MHz): *δ* 27.7 (CH_2_(Ad)), 36.0 (CH(Ad)), 38.7 (CH_2_(Ad)), 46.0 (C(Ad)), 119.5 (CH(Ph)), 128.8 (CH(Ph)), 132.4 (CH(Ph)), 140.4 (C(Ph)), 141.5 (C), 142.9 (C), 149.0 (C), 149.2 (C), 149.5 (C), 206.3 (Ph**C**OAd) ppm. IR (KBr): 2906 (s), 2850 (m), 1649 (m), 1626 (m), 1585 (s), 1572 (s), 1533 (m), 1460 (s), 1412 (w), 1321 (s), 1236 (s), 1174 (m), 930 (m), 805 (w), 627 (m) cm^−1^. ESI-MS (pos.) *m*/*z* (%): 853.1 [2·M(^35^Cl) + K^+^]^+^ (5), 837.2 [2·M(^35^Cl) + Na^+^]^+^ (33), 446.1 [M(^35^Cl) + K^+^]^+^ (63), 430.1 [M(^35^Cl) + Na^+^]^+^ (100), 408.1 [M(^35^Cl) + H^+^]^+^ (18). ESI-MS (neg.) *m*/*z* (%): 813.1 [2·M(^35^Cl) − H^+^]^−^ (8), 406.1 [M(^35^Cl) − H^+^]^−^ (100). Anal. Cald for C_22_H_22_ClN_5_O: C 64.78; H 5.44; N 17.17. Found: C 64.75; H 5.19; N 17.47.

(1-Adamantyl){3-[(2-chloro-9*H*-purin-6-yl)-amino]phenyl}methanone (**9**)

Prepared from compound **3** (60 mg, 0.32 mmol) and (1-adamantyl)(3-aminophenyl)methanone (0.64 mmol). Purified using column chromatography (system d) and crystallization from i-PrOH. Pale yellow crystalline powder, yield 123 mg (94%), mp 332–338 °C, R_f_ = 0.44 (system d). ^1^H NMR (DMSO-*d*_6_, 500 MHz): *δ* 1.73 (m, 6H, CH_2_(Ad)), 1.99 (m, 6H, CH_2_(Ad)), 2.04 (m, 3H, CH(Ad)), 7.21 (d, 1H, *J* = 7.65 Hz, Ph), 7.43 (t, 1H, *J* = 7.90 Hz, Ph), 7.91 (d, 1H, *J* = 6.55 Hz, Ph), 8.32 (s, 1H, Ph), 8.34 (s, 1H, C^8^H), 10.35 (s, 1H, C^6^N**H**Ph), 13.35 (s, 1H, N^9^H) ppm. ^13^C NMR (DMSO-*d*_6_, 125 MHz): *δ* 27.5 (CH_2_(Ad)), 35.9 (CH(Ad)), 38.5 (CH_2_(Ad)), 46.1 (C(Ad)), 119.2 (CH(Ph)), 122.1 (CH(Ph)), 122.6 (CH(Ph)), 128.6 (CH(Ph)), 138.3 (C(Ph)), 138.9 (C(Ph)), 152.0 (C), 208.3 (Ph**C**OAd) ppm. IR (KBr): 3345 (s), 2901 (m), 1662 (m), 1627 (s), 1583 (s), 1480 (m), 1430 (m), 1391 (w), 1348 (m), 1310 (s), 1243 (m), 1108 (w), 952 (m), 788 (m), 638 (w) cm^−1^. ESI-MS (pos.) *m*/*z* (%): 837.2 [2·M(^35^Cl) + Na^+^]^+^ (14), 446.1 [M(^35^Cl) + K^+^]^+^ (38), 430.1 [M(^35^Cl) + Na^+^]^+^ (100), 408.1 [M(^35^Cl) + H^+^]^+^ (26). ESI-MS (neg.) *m*/*z* (%): 813.1 [2·M(^35^Cl) − H^+^]^−^ (9), 406.0 [M(^35^Cl) − H^+^]^−^ (100). Anal. Cald for C_22_H_22_ClN_5_O: C 64.78; H 5.44; N 17.17. Found: C 64.54; H 5.42; N 17.36.

### 3.4. General Procedures for the Glycosylation of 6-‘Amino’-9H-purines (**4**–**9**)

Compounds **10**–**15** were prepared following modified literature procedures, hereinafter referred to as “method A” [44] and “method B” [45], respectively.

“Method A”

The corresponding 6-‘amino’-9*H*-purine (0.20–0.27 mmol) and commercially available 1-*O*-2,3,5-tri-*O*-benzoyl-β-d-ribofuranose (3.0 equiv.) were added into the anhydrous acetonitrile (5 cm^3^). Into this suspension, trimethylsilyl trifluoromethanesulfonate (2.0 equiv.) was added in one portion, using a syringe. The resulting pale yellow solution was stirred at room temperature under an argon atmosphere till the TLC indicated the consumption of all starting material (4–6 h). After this, 5 cm^3^ of distilled water was added and the reaction mixture was stirred for 15 minutes. Subsequently, the mixture was extracted with ethyl acetate (7 × 10 cm^3^). The combined organic layers were washed twice with brine and dried over sodium sulfate. The solvent was removed by the evaporation in a vacuum. The desired compound was obtained after purification of the crude product using column chromatography.

“Method B”

The corresponding 6-‘amino’-9*H*-purine (0.24–0.72 mmol) was added into the anhydrous acetonitrile (5 cm^3^). Into this suspension, *N*,*O*-bis(trimethylsilyl)acetamide (2.0 equiv.) was added in one portion, using a syringe. The resulting solution was stirred at 75 °C under an argon atmosphere for 6 h. After this, a mixture of commercially available 1-*O*-2,3,5-tri-*O*-benzoyl-β-d-ribofuranose (1.1 equiv.) dissolved in anhydrous acetonitrile (1 cm^3^) and trimethylsilyl trifluoromethanesulfonate (1.0 equiv.) was added dropwise into the reaction mixture. The reaction mixture was stirred under the same conditions till the TLC indicated the consumption of all starting material (2–6 h). After this, 10 cm^3^ of distilled water was added and the reaction mixture was stirred for 15 minutes. Subsequently, the mixture was extracted with ethyl acetate (9 × 10 cm^3^). The combined organic layers were washed twice with brine and dried over sodium sulfate. The solvent was removed by evaporation in a vacuum. The desired compound was obtained after purification of the crude product using column chromatography.

*N*-phenyl-9-(2,3,5-tri-*O*-benzoyl-β-d-ribofuranos-1-yl)-9*H*-purin-6-amine (**10**)

Prepared using “method A” from compound **4** (50 mg, 0.24 mmol) and 1-*O*-2,3,5-tri-*O*-benzoyl-β-d-ribofuranose (0.72 mmol) and trimethylsilyl trifluoromethanesulfonate (0.48 mmol), and using “method B” from compound **4** (50 mg, 0.24 mmol), *N*,*O*-bis(trimethylsilyl)acetamide (0.48 mmol), and 1-*O*-2,3,5-tri-*O*-benzoyl-β-d-ribofuranose (0.24 mmol) and trimethylsilyl trifluoromethanesulfonate (0.24 mmol). Purified using column chromatography (system e). Colorless crystalline powder, yield (method A) 83 mg (54%), (method B) 48 mg (31%), mp 75–83 °C, R_f_ = 0.68 (system e). ^1^H NMR (DMSO-*d*_6_, 500 MHz): *δ* 4.67–4.70 (m, 1H, C^5′^H(Rib)), 4.81–4.84 (m, 1H, C^5′^H(Rib)), 4.89 (ddd, 1H, *J*_1_ = 4.30 Hz, *J*_2_ = 9.50 Hz, C^4′^H(Rib)), 6.31 (dd, 1H, *J*_1_ = 5.95 Hz, *J*_2_ = 11.90 Hz, C^3′^H(Rib)), 6.55 (dd, 1H, *J*_1_ = 5.00 Hz, *J*_2_ = 10.65 Hz, C^2′^H(Rib)), 6.63 (d, 1H, *J* = 4.40 Hz, C^1′^H(Rib)), 7.06 (t, 1H, *J* = 7.35 Hz, Ph), 7.34 (t, 2H, *J* = 7.65 Hz, Ph), 7.45–7.50 (m, 6H, Bz), 7.63–7.68 (m, 3H, Bz), 7.89–7.96 (m, 6H, Bz), 8.00 (d, 2H, *J* = 7.05 Hz, Ph), 8.32 (s, 1H, C^2^H), 8.57 (s, 1H, C^8^H), 9.96 (s, 1H, C^6^N**H**Ph) ppm. ^13^C NMR (DMSO-*d*_6_, 125 MHz): *δ* 63.2 (CH(Rib)), 70.7 (CH(Rib)), 73.1 (CH(Rib)), 79.2 (CH(Rib)), 86.5 (CH(Rib)), 120.4 (CH(Ph)), 121.0 (CH(Ph)), 122.8 (CH(Ph)), 128.3 (CH(Bz)), 128.5 (CH(Ph)), 128.6 (CH(Bz)), 128.7 (CH(Bz)), 129.2 (CH(Bz)), 129.3 (CH(Bz)), 133.4 (C(Bz)), 133.8 (C(Bz)), 133.9 (C(Bz)), 139.4 (C), 141.2 (C), 149.1 (C), 152.2 (C), 164.5 (CO(Bz)), 164.6 (CO(Bz)), 165.4 (CO(Bz)) ppm. IR (KBr): 3348 (bw), 3059 (w), 2926 (w), 1727 (s), 1622 (s), 1583 (s), 1477 (m), 1374 (m), 1268 (s), 1178 (m), 1122 (s), 1026 (m), 751 (w), 710 (s), 645 (w) cm^−1^. ESI-MS (pos.) *m*/*z* (%): 694.2 [M + K^+^]^+^ (5), 678.3 [M + Na^+^]^+^ (12), 656.3 [M + H^+^]^+^ (100). ESI-MS (neg.) *m*/*z* (%): 654.2 [M − H^+^]^−^ (100). Anal. Cald for C_37_H_29_N_5_O_7_: C 67.78; H 4.46; N 10.68. Found: C 67.90; H 4.42; N 10.78.

(1-Adamantyl){4-[(9-(2,3,5-tri-*O*-benzoyl-β-d-ribofuranos-1-yl)-9*H*-purin-6-yl)amino]phenyl}methanone (**11**)

Prepared using “method A” from compound **5** (101 mg, 0.27 mmol) and 1-*O*-2,3,5-tri-*O*-benzoyl-β-d-ribofuranose (0.81 mmol) and trimethylsilyl trifluoromethanesulfonate (0.54 mmol). Purified using column chromatography (system a). Colorless crystalline powder, yield 164 mg (73%), mp 103–113 °C, R_f_ = 0.47 (system a). ^1^H NMR (DMSO-*d*_6_, 500 MHz): *δ* 1.68–1.75 (m, 6H, CH_2_(Ad)), 1.98 (m, 6H, CH_2_(Ad)), 2.02 (m, 3H, CH(Ad)), 4.66–4.69 (m, 1H, C^5′^H(Rib)), 4.81–4.84 (m, 1H, C^5′^H(Rib)), 4.89 (ddd, 1H, *J*_1_ = 1.70 Hz, *J*_2_ = 5.80 Hz, *J_3_* = 8.25 Hz, C^4′^H(Rib)), 6.31 (dd, 1H, *J*_1_ = 6.15 Hz, *J*_2_ = 11.95 Hz, C^3′^H(Rib)), 6.55 (dd, 1H, *J_1_* = 4.60 Hz, *J*_2_ = 5.80 Hz, C^2′^H(Rib)), 6.65 (d, 1H, *J* = 4.25 Hz, C^1′^H(Rib)), 7.44–7.52 (m, 6H, Bz), 7.63–7.68 (m, 3H, Bz), 7.77 (d, 2H, *J* = 8.85 Hz, Ph), 7.89–8.01 (m, 6H, Bz), 8.06 (d, 2H, *J* = 8.90 Hz, Ph), 8.37 (s, 1H, C^2^H), 8.62 (s, 1H, C^8^H), 10.32 (s, 1H, C^6^N**H**Ph) ppm. ^13^C NMR (DMSO-*d*_6_, 125 MHz): *δ* 27.7 (CH_2_(Ad)), 36.0 (CH(Ad)), 38.8 (CH_2_(Ad)), 46.0 (C(Ad)), 61.5 (CH(Rib)), 70.7 (CH(Rib)), 73.1 (CH(Rib)), 85.4 (CH(Rib)), 86.6 (CH(Rib)), 119.5 (CH(Ph)), 120.7 (CH(Ph)), 123.2 (CH(Ph)), 128.5 (CH(Ph)), 128.7 (C(Bz)), 128.8 (C(Bz)), 128.9 (C(Bz)), 129.3 (CH(Bz)), 129.4 (CH(Bz)), 133.5 (C(Bz)), 133.9 (C(Bz)), 134.0 (C(Bz)), 141.8 (C), 149.4 (C), 151.0 (C), 152.0 (C), 152.2 (C), 164.7 (CO (Bz)), 164.9 (CO (Bz)), 165.6 (CO (Bz)), 208.2 (Ph**C**OAd) ppm. IR (KBr): 2905 (s), 2851 (s), 1729 (s), 1655 (w), 1605 (m), 1580 (s), 1473 (m), 1370 (m), 1270 (s), 1176 (m), 1121 (m), 1094 (m), 1026 (m), 987 (w), 711 (s) cm^−1^. ESI-MS (pos.) *m*/*z* (%): 856.3 [M + K^+^]^+^ (6), 840.3 [M + Na^+^]^+^ (16), 818.4 [M + H^+^]^+^ (100). ESI-MS (neg.) *m*/*z* (%): 816.3 [M − H^+^]^−^ (100). Anal. Cald for C_48_H_43_N_5_O_8_: C 70.49; H 5.30; N 8.56. Found: C 70.66; H 5.27; N 8.41.

(1-Adamantyl){3-[(9-(2,3,5-tri-*O*-benzoyl-β-d-ribofuranos-1-yl)-9*H*-purin-6-yl)amino]phenyl}methanone (**12**)

Prepared using “method A” from compound **6** (101 mg, 0.27 mmol) and 1-*O*-2,3,5-tri-*O*-benzoyl-β-d-ribofuranose (0.81 mmol) and trimethylsilyl trifluoromethanesulfonate (0.54 mmol). Purified using column chromatography (system a). Colorless crystalline powder, yield 130 mg (59%), mp 94–105 °C, R_f_ = 0.50 (system a). ^1^H NMR (DMSO-*d*_6_, 500 MHz): *δ* 1.69–1.71 (m, 6H, CH_2_(Ad)), 1.96–1.98 (m, 6H, CH_2_(Ad)), 2.03 (m, 3H, CH(Ad)), 4.65–4.69 (m, 1H, C^5′^H(Rib)), 4.81–4.84 (m, 1H, C^5′^H(Rib)), 4.89 (ddd, 1H, *J*_1_ = 3.65 Hz, *J*_2_ = 8.25 Hz, C^4′^H(Rib)), 6.31 (dd, 1H, *J*_1_ = 5.85 Hz, *J*_2_ = 11.95 Hz, C^3′^H(Rib)), 6.56 (dd, 1H, *J*_1_ = 4.60 Hz, *J*_2_ = 10.40 Hz, C^2′^H(Rib)), 6.65 (d, 1H, *J* = 4.55 Hz, C^1′^H(Rib)), 7.22 (d, 1H, *J* = 6.45 Hz, Ph), 7.37–7.57 (m, 6H, Bz), 7.63 (t, 1H, *J* = 1.25 Hz, Ph), 7.64–7.71 (m, 3H, Bz), 7.86–8.01 (m, 6H, Bz), 8.10 (d, 1H, *J* = 7.05 Hz, Ph), 8.30 (s, 1H, Ph), 8.38 (s, 1H, C^2^H), 8.60 (s, 1H, C^8^H), 10.23 (s, 1H, C^6^N**H**Ph) ppm. ^13^C NMR (DMSO-*d*_6_, 125 MHz): *δ* 28.1 (CH_2_(Ad)), 36.5 (CH(Ad)), 39.1 (CH_2_(Ad)), 46.7 (C(Ad)), 63.3 (CH(Rib)), 70.7 (CH(Rib)), 73.1 (CH(Rib)), 82.9 (CH(Rib)), 86.5 (CH(Rib)), 119.9 (CH(Ph)), 121.1 (CH(Ph)), 123.2 (CH(Ph)), 128.5 (CH(Ph)), 129.0 (CH(Bz)), 129.1 (CH(Bz)), 129.2 (CH(Bz)), 129.3 (CH(Bz)), 129.5 (CH(Bz)), 129.7 (CH(Bz)), 129.8 (C(Ph)), 130.0 (C(Ph)), 134.0 (C(Bz)), 134.4 (C(Bz)), 134.5 (C(Bz)), 139.1 (C), 139.5 (C), 142.1 (C), 149.8 (C), 152.7 (C), 164.5 (CO(Bz)), 164.7 (CO(Bz)), 165.4 (CO(Bz)), 208.1 (Ph**C**OAd) ppm. IR (KBr): 2905 (s), 2851 (m), 1728 (s), 1667 (w), 1621 (m), 1602 (m), 1580 (s), 1474 (m), 1371 (m), 1270 (s), 1177 (m), 1122 (m), 1026 (m), 998 (w), 711 (s) cm^−1^. ESI-MS (pos.) *m*/*z* (%): 856.3 [M + K^+^]^+^ (7), 840.3 [M + Na^+^]^+^ (19), 818.4 [M + H^+^]^+^ (100). ESI-MS (neg.) *m*/*z* (%): 816.3 [M − H^+^]^−^ (100). Anal. Cald for C_48_H_43_N_5_O_8_: C 70.49; H 5.30; N 8.56. Found: C 70.68; H 5.42; N 8.48.

*N*-phenyl-2-chloro-9-(2,3,5-tri-*O*-benzoyl-β-d-ribofuranos-1-yl)-9*H*-purin-6-amine (**13**)

Prepared using “method A” from compound **7** (50 mg, 0.20 mmol) and 1-*O*-2,3,5-tri-*O*-benzoyl-β-d-ribofuranose (0.60 mmol) and trimethylsilyl trifluoromethanesulfonate (0.40 mmol), and using “method B” from compound **7** (60 mg, 0.24 mmol), *N*,*O*-bis(trimethylsilyl)acetamide (0.48 mmol), 1-*O*-2,3,5-tri-*O*-benzoyl-β-d-ribofuranose (0.26 mmol), and trimethylsilyl trifluoromethanesulfonate (0.48 mmol). Purified using column chromatography (system e). Colorless crystalline powder, yield (method A) 71 mg (51%), (method B) 164 mg (97%), mp 105–113 °C, R_f_ = 0.57 (system e). ^1^H NMR (DMSO-*d*_6_, 500 MHz): *δ* 4.68–4.71 (m, 1H, C^5′^H(Rib)), 4.79–4.82 (m, 1H, C^5′^H(Rib)), 4.90 (ddd, 1H, *J*_1_ = 4.25 Hz, *J*_2_ = 9.65 Hz, C^4′^H(Rib)), 6.24 (dd, 1H, *J*_1_ = 6.05 Hz, *J*_2_ = 10.95 Hz, C^3′^H(Rib)), 6.37 (dd, 1H, *J*_1_ = 5.15 Hz, *J*_2_ = 10.55 Hz, C^2′^H(Rib)), 6.60 (d, 1H, *J* = 4.30 Hz, C^1′^H(Rib)), 7.12 (t, 1H, *J* = 7.20 Hz, Ph), 7.37 (t, 2H, *J* = 7.15 Hz, Ph), 7.45–7.48 (m, 6H, Bz), 7.61–7.67 (m, 3H, Bz), 7.83 (d, 2H, *J* = 7.65 Hz, Ph), 7.91–7.96 (m, 6H, Bz), 8.58 (s, 1H, C^8^H), 10.39 (s, 1H, C^6^N**H**Ph) ppm. ^13^C NMR (DMSO-*d*_6_, 125 MHz): *δ* 63.4 (CH(Rib)), 67.1 (CH(Rib)), 70.9 (CH(Rib)), 75.05 (CH(Rib)), 79.7 (CH(Rib)), 119.2 (CH(Ph)), 121.4 (CH(Ph)), 123.6 (CH(Ph)), 128.4 (CH(Bz)), 128.5 (CH(Ph)), 128.6 (CH(Bz)), 128.7 (CH(Bz)), 129.2 (CH(Bz)), 129.3 (CH(Bz)), 129.5 (CH(Bz)), 129.7 (CH(Bz)), 133.5 (C(Bz)), 133.9 (C(Bz)), 134.6 (C(Bz)), 141.4 (C), 152.2 (C), 152.5 (C), 163.7 (CO(Bz)), 164.4 (CO(Bz)), 165.3 (CO(Bz)) ppm. IR (KBr): 3338 (w, b), 3061 (bw), 1728 (s), 1623 (s), 1581 (s), 1498 (s), 1452 (s), 1316 (s), 1268 (s), 1178 (m), 1121 (s), 1094 (s), 1026 (m), 941 (w), 710 (s) cm^−1^. Anal. Cald for C_37_H_28_ClN_5_O_7_: C 64.40; H 4.09; N 10.15. Found: C 64.56; H 4.18; N 10.09.

(1-Adamantyl){4-[(2-chloro-9-(2,3,5-tri-*O*-benzoyl-β-d-ribofuranos-1-yl)-9*H*-purin-6-yl)amino]phenyl}methanone (**14**)

Prepared using “method B” from compound **8** (92 mg, 0.72 mmol), *N*,*O*-bis(trimethylsilyl)acetamide (1.44 mmol), 1-*O*-2,3,5-tri-*O*-benzoyl-β-d-ribofuranose (0.79 mmol), and trimethylsilyl trifluoromethanesulfonate (0.72 mmol). Purified using column chromatography (system a). Colorless crystalline powder, yield 171 mg (89%), mp 117–124 °C, R_f_ = 0.54 (system a). ^1^H NMR (DMSO-*d*_6_, 500 MHz): *δ* 1.69–1.75 (m, 6H, CH_2_(Ad)), 1.96–1.98 (m, 6H, CH_2_(Ad)), 2.03 (m, 3H, CH(Ad)), 4.6–4.71 (m, 1H, C^5′^H(Rib)), 4.80–4.83 (m, 1H, C^5′^H(Rib)), 4.90 (ddd, 1H, *J*_1_ = 4.85 Hz, *J*_2_ = 9.45 Hz, C^4′^H(Rib)), 6.24 (dd, 1H, *J*_1_ = 5.95 Hz, *J*_2_ = 11.50 Hz, C^3′^H(Rib)), 6.37 (dd, 1H, *J*_1_ = 4.60 Hz, *J*_2_ = 5.95 Hz, C^2′^H(Rib)), 6.61 (d, 1H, *J* = 4.40 Hz, C^1′^H(Rib)), 7.45–7.49 (m, 6H, Bz), 7.63–7.69 (m, 3H, Bz), 7.75 (d, 2H, *J* = 8.85 Hz, Ph), 7.92 (d, 2H, *J* = 7.20 Hz, Ph), 7.94–7.96 (m, 6H, Bz), 8.63 (s, 1H, C^8^H), 10.65 (s, 1H, C^6^N**H**Ph) ppm. ^13^C NMR (DMSO-*d*_6_, 125 MHz): *δ* 27.6 (CH_2_(Ad)), 35.9 (CH(Ad)), 38.7 (CH_2_(Ad)), 46.0 (C(Ad)), 63.3 (CH(Rib)), 70.7 (CH(Rib)), 73.5 (CH(Rib)), 79.4 (CH(Rib)), 86.3 (CH(Rib)), 119.6 (CH(Ph)), 120.0 (CH(Ph)), 128.3 (CH(Ph)), 128.6 (CH(Bz)), 128.7 (CH(Bz)), 128.7 (CH(Bz)), 129.2 (CH(Bz)), 129.3 (CH(Bz)), 129.4 (CH(Bz)), 132.9 (C(Ph)), 133.8 (C(Bz)), 133.9 (C(Bz)), 141.0 (C), 141.5 (C), 150.4 (C), 152.2 (C), 152.6 (C), 164.5 (CO(Bz)), 164.6 (CO(Bz)), 165.4 (CO(Bz)), 206.4 (Ph**C**OAd) ppm. IR (KBr): 2905 (s), 2851 (s), 1729 (s), 1604 (m), 1574 (s), 1506 (w), 1452 (m), 1317 (m), 1270 (s), 1176 (m), 1120 (m), 1094 (m), 1026 (m), 987 (w), 711 (s) cm^−1^. Anal. Cald for C_48_H_42_ClN_5_O_8_: C 67.64; H 4.97; N 8.22. Found: C 67.33; H 5.02; N 8.45.

(1-Adamantyl){3-[(2-chloro-9-(2,3,5-tri-*O*-benzoyl-β-d-ribofuranos-1-yl)-9*H*-purin-6-yl)amino]phenyl}methanone (**15**)

Prepared using “method B” from compound **9** (60 mg, 0.41 mmol), *N*,*O*-bis(trimethylsilyl)acetamide (0.82 mmol), 1-*O*-2,3,5-tri-*O*-benzoyl-β-d-ribofuranose (0.45 mmol), and trimethylsilyl trifluoromethanesulfonate (0.41 mmol). Purified using column chromatography (system g). Colorless crystalline powder, yield 101 mg (81%), mp 95–106 °C, R_f_ = 0.40 (system g). ^1^H NMR (DMSO-*d*_6_, 500 MHz): *δ* 1.68–1.70 (m, 6H, CH_2_(Ad)), 1.98 (m, 6H, CH_2_(Ad)), 2.02 (m, 3H, CH(Ad)), 3.67–3.71 (m, 1H, C^5′^H(Rib)), 3.79–3.82 (m, 1H, C^5′^H(Rib)), 4.90 (ddd, 1H, *J*_1_ = 4.90 Hz, *J*_2_ = 9.45 Hz, C^4′^H(Rib)), 6.24 (dd, 1H, *J*_1_ = 5.80 Hz, *J_2_* = 11.90 Hz, C^3′^H(Rib)), 6.37 (dd, 1H, *J*_1_ = 4.60 Hz, *J*_2_ = 5.80 Hz, C^2′^H(Rib)), 6.61 (d, 1H, *J* = 4.30 Hz, C^1′^H(Rib)), 7.24 (d, 1H, *J* = 7.65 Hz, Ph), 7.44 (t, 1H, *J* = 8.25 Hz, Ph), 7.45–7.49 (m, 6H, Bz), 7.60–7.67 (m, 3H, Bz), 7.90 (d, 1H, *J* = 10.7 Hz, Ph), 7.91 (m, 6H, Bz), 8.32 (s, 1H, Ph), 8.62 (s, 1H, C^8^H), 10.62 (s, 1H, C^6^N**H**Ph) ppm. ^13^C NMR (DMSO-*d*_6_, 125 MHz): *δ* 27.6 (CH_2_(Ad)), 35.9 (CH(Ad)), 38.5 (CH_2_(Ad)), 46.2 (C(Ad)), 63.3 (CH(Rib)), 70.7 (CH(Rib)), 73.7 (CH(Rib)), 79.4 (CH(Rib)), 86.3 (CH(Rib)), 119.5 (CH(Ph)), 119.8 (CH(Ph)), 123.1 (CH(Ph)), 128.4 (CH(Ph)), 128.5 (CH(Ph)), 128.6 (CH(Bz)), 128.7 (CH(Bz)), 128.8 (CH(Bz)), 129.0 (CH(Bz)), 129.2 (CH(Bz)), 129.4 (CH(Bz)), 133.5 (C(Bz)), 133.9 (C(Bz)), 134.0 (C(Bz)), 138.0 (C(Ph)), 138.8 (C(Ph)), 141.4 (C), 152.5 (C), 152.7 (C), 164.5 (CO(Bz)), 164.6 (CO(Bz)), 165.4 (CO(Bz)), 208.2 (Ph**C**OAd) ppm. IR (KBr): 2905 (s), 2851 (m), 1728 (s), 1669 (m), 1623 (s), 1575 (s), 1452 (s), 1316 (s), 1270 (s), 1177 (m), 1121 (s), 1094 (s), 1070 (s), 788 (w), 711 (s) cm^−1^. Anal. Cald for C_48_H_42_ClN_5_O_8_: C 67.64; H 4.97; N 8.22. Found: C 67.33; H 5.08; N 8.49.

### 3.5. General Procedure for the Deprotection of Purines **10**–**15**

Sodium methoxide (1 M solution, 0.2 equiv.) was added into the mixture of the corresponding purine (0.17–0.42 mmol) in methanol (10 cm^3^). The reaction mixture was vigorously stirred at room temperature till the TLC indicated the consumption of all starting material (14–18 h). After this, the solvent was removed by evaporation in a vacuum. The desired compound was obtained after purification of the crude product using column chromatography.

*N*-phenyl-9-β-d-ribofuranos-1-yl-9*H*-purin-6-amine (**16**)

Prepared from compound **10** (230 mg, 0.30 mmol), 1 M sodium methoxide (0.06 mmol). Purified using column chromatography (system f). Colorless crystalline powder, yield 82 mg (77%), mp 196–198 °C, R_f_ = 0.40 (system f). ^1^H NMR (DMSO-*d*_6_, 500 MHz): *δ* 3.58–3.62 (m, 1H, C^5‘^H(Rib)), 3.69–3.74 (m, 1H, C^5‘^H(Rib)), 4.00 (ddd, 1H, *J*_1_ = 3.14 Hz, *J*_2_ = 6.65 Hz, C^4′^H(Rib)), 4.20 (dd, 1H, *J*_1_ = 4.35 Hz, *J*_2_ = 7.86 Hz, C^3′^H(Rib)), 4.66 (dd, 1H, *J*_1_ = 5.65 Hz, *J*_2_ = 10.79 Hz, C^2′^H(Rib)), 5.22 (d, 1H, *J* = 3.85 Hz, C^3′^HO**H**(Rib)), 5.30 (dd, 1H, *J*_1_ = 4.55 Hz, *J*_2_ = 10.10 Hz, C^5′^H_2_O**H**(Rib)), 5.49 (d, 1H, *J* = 6.10 Hz, C^2′^HO**H**(Rib)), 5.98 (d, 1H, *J* = 5.75 Hz, C^1′^H(Rib)), 7.06 (t, *J* = 7.30 Hz, 1H, Ph), 7.34 (t, *J* = 7.65 Hz, 2H, Ph), 7.94 (d, *J* = 7.95 Hz, 2H, Ph), 8.41 (s, 1H, C^2^H), 8.53 (s, 1H, C^8^H), 9.91 (s, 1H, C^6^N**H**Ph) ppm. ^13^C NMR (DMSO-*d*_6_, 125 MHz): *δ* 61.6 (CH(Rib)), 70.6 (CH(Rib)), 73.7 (CH(Rib)), 85.9 (CH(Rib)), 87.9 (CH(Rib)), 120.4 (CH(Ph)), 120.9 (CH(Ph)), 122.8 (CH(Ph)), 128.4 (CH(Ph)), 139.5 (C(Ph)), 140.7 (C), 149.4 (C), 151.9 (C), 152.2 (C) ppm. IR (KBr): 3334 (bm), 2922 (m), 1627 (s), 1588 (s), 1499 (s), 1480 (s), 1441 (m), 1376 (m), 1234 (m), 1099 (m), 791 (w), 749 (m), 691 (m), 669 (m), 638 (m) cm^−1^. ESI-MS (pos.) *m*/*z* (%): 366.0 [M + Na^+^]^+^ (9), 344.1 [M + H^+^]^+^ (100). ESI-MS (neg.) *m*/*z* (%): 685.1 [2·M − H^+^]^−^ (95), 341.9 [M − H^+^]^−^ (100). Anal. Cald for C_16_H_17_N_5_O_4_: C 55.97; H 4.99; N 20.40. Found: C 55.73; H 5.02; N 20.38.

(1-Adamantyl){4-[(9-β-d-ribofuranos-1-yl-9*H*-purin-6-yl)amino]phenyl}methanone (**17**)

Prepared from compound **11** (140 mg, 0.17 mmol), 1 M sodium methoxide (0.03 mmol). Purified using column chromatography (system c). Colorless crystalline powder, yield 80 mg (92%), mp 145–155 °C, R_f_ = 0.28 (system c). ^1^H NMR (DMSO-*d*_6_, 500 MHz): *δ* 1.69–1.75 (m, 6H, CH_2_(Ad)), 1.98–1.99 (m, 6H, CH_2_(Ad)), 2.03 (m, 3H, CH(Ad)), 3.56–3.61 (m, 1H, C^5′^H(Rib)), 3.69–3.73 (m, 1H, C^5′^H(Rib)), 3.99 (ddd, 1H, *J*_1_ = 3.65 Hz, *J*_2_ = 7.10 Hz, C^4′^H(Rib)), 4.19 (ddd, 1H, *J*_1_ = 4.80 Hz, *J*_2_ = 8.15 Hz, C^3′^H(Rib)), 4.65 (dd, 1H, *J*_1_ = 5.95 Hz, *J*_2_ = 11.05 Hz, C^2′^H(Rib)), 5.23 (d, 1H, *J* = 4.80 Hz, C^3′^HO**H**(Rib)), 5.27 (dd, 1H, *J*_1_ = 4.90 Hz, *J*_2_ = 6.65 Hz, C^5′^H_2_O**H**(Rib)), 5.52 (d, 1H, *J* = 6.15 Hz, C^2′^HO**H**(Rib)), 5.98 (d, 1H, *J* = 6.00 Hz, C^1′^H(Rib)), 7.77 (d, 2H, *J* = 8.90 Hz, Ph), 8.09 (d, 2H, *J* = 8.90 Hz, Ph), 8.48 (s, 1H, C^2^H), 8.61 (s, 1H, C^8^H), 10.30 (s, 1H, C^6^N**H**Ph) ppm. ^13^C NMR (DMSO-*d*_6_, 125 MHz): *δ* 27.7 (CH_2_(Ad)), 36.0 (CH(Ad)), 38.8 (CH_2_(Ad)), 46.0 (C(Ad)), 61.5 (CH(Rib)), 70.5 (CH(Rib)), 73.7 (CH(Rib)), 85.9 (CH(Rib)), 87.9 (CH(Rib)), 119.4 (CH(Ph)), 120.7 (CH(Ph)), 128.9 (CH(Ph)), 131.7 (CH(Ph)), 141.2 (C(Ph)), 142.3 (C), 149.7 (C), 151.9 (C), 152.2 (C), 206.1 (Ph**C**OAd) ppm. IR (KBr): 3397 (s, b), 2905 (s), 2851 (s), 1628 (s), 1606 (s), 1581 (s), 1474 (s), 1414 (m), 1370 (m), 1272 (m), 1238 (s), 1175 (m), 1083 (m), 987 (w), 750 (m) cm^−1^. ESI-MS (pos.) *m*/*z* (%): 528.2 [M + Na^+^]^+^ (8), 506.2 [M + H^+^]^+^ (100). ESI-MS (neg.) *m*/*z* (%): 1045.3 [2·M + Cl^−^]^−^ (8), 1009.4 [2·M − H^+^]^−^ (12), 540.1 [M + Cl^−^]^−^ (37), 504.2 [M − H^+^]^−^ (100). Anal. Cald for C_27_H_31_N_5_O_5_: C 64.14; H 6.18; N 13.85. Found: C 64.15; H 6.33; N 13.54.

(1-Adamantyl){3-[(9-β-d-ribofuranos-1-yl-9*H*-purin-6-yl)amino]phenyl}methanone (**18**)

Prepared from compound **12** (144 mg, 0.18 mmol), 1 M sodium methoxide (0.04 mmol). Purified using column chromatography (system c). Colorless crystalline powder, yield 84 mg (94%), mp 120–130 °C, R_f_ = 0.34 (system c). ^1^H NMR (DMSO-*d*_6_, 500 MHz): *δ* 1.71–1.72 (m, 6H, CH_2_(Ad)), 1.98–1.99 (m, 6H, CH_2_(Ad)), 2.04 (m, 3H, CH(Ad)), 3.56–3.61 (m, 1H, C^5′^H(Rib)), 3.68–3.73 (m, 1H, C^5′^H(Rib)), 3.99 (ddd, 1H, *J*_1_ = 3.65 Hz, *J*_2_ = 7.35 Hz, C^4′^H(Rib)), 4.18 (dd, 1H, *J*_1_ = 3.35 Hz, *J*_2_ = 4.55 Hz, C^3′^H(Rib)), 4.64 (dd, 1H, *J*_1_ = 6.15 Hz, *J*_2_ = 11.35 Hz, C^2′^H(Rib)), 5.24 (d, 1H, *J* = 4.90 Hz, C^3′^HO**H**(Rib)), 5.28 (dd, 1H, *J*_1_ = 4.90 Hz, *J*_2_ = 6.70 Hz, C^5′^H_2_O**H**(Rib)), 5.51 (d, 1H, *J* = 6.10 Hz, C^2′^HO**H**(Rib)), 5.98 (d, 1H, *J* = 6.10 Hz, C^1′^H(Rib)), 7.22 (d, 1H, *J* = 7.65 Hz, Ph), 7.41 (t, 1H, *J* = 7.95 Hz, Ph), 8.15 (d, 1H, *J* = 8.25 Hz, Ph), 8.36 (s, 1H, Ph), 8.43 (s, 1H, C^2^H), 8.59 (s, 1H, C^8^H), 10.19 (s, 1H, C^6^N**H**Ph) ppm. ^13^C NMR (DMSO-*d*_6_, 125 MHz): *δ* 28.1 (CH_2_(Ad)), 36.5 (CH(Ad)), 39.1 (CH_2_(Ad)), 46.7 (C(Ad)), 62.0 (CH(Rib)), 71.0 (CH(Rib)), 74.2 (CH(Rib)), 86.4 (CH(Rib)), 88.3 (CH(Rib)), 119.8 (CH(Ph)), 121.1 (CH(Ph)), 122.0 (CH(Ph)), 123.0 (CH(Ph)), 128.9 (C(Ph)), 139.2 (C(Ph)), 139.7 (C), 141.4 (C), 150.0 (C), 152.3 (C), 152.5 (C), 208.7 (Ph**C**OAd) ppm. IR (KBr): 3347 (bs), 2906 (s), 2851 (m), 1625 (s), 1602 (s), 1577 (s), 1478 (s), 1432 (m), 1372 (m), 1331 (m), 1296 (m), 1227 (m), 1084 (m), 998 (m), 755 (m) cm^−1^. ESI-MS (pos.) *m*/*z* (%): 528.2 [M + Na^+^]^+^ (5), 506.2 [M + H^+^]^+^ (100). ESI-MS (neg.) *m*/*z* (%): 1045.3 [2·M + Cl^−^]^−^ (6), 1009.3 [2·M − H^+^]^−^ (11), 540.1 [M + Cl^−^]^−^ (22), 504.1 [M − H^+^]^−^ (100). Anal. Cald for C_27_H_31_N_5_O_5_: C 64.14; H 6.18; N 13.85. Found: C 64.03; H 6.47; N 13.74.

*N*-phenyl-2-chloro-9-β-d-ribofuranos-1-yl-9*H*-purin-6-amine (**19**)

Prepared from compound **13** (289 mg, 0.42 mmol), 1 M sodium methoxide (0.08 mmol). Purified using column chromatography (system i). Colorless crystalline powder, yield 151 mg (95%), mp 160–162 °C, R_f_ = 0.42 (system i). ^1^H NMR (DMSO-*d*_6_, 500 MHz): *δ* 3.57–3.60 (m, 1H, C^5‘^H(Rib)), 3.68–3.71 (m, 1H, C^5‘^H(Rib)), 3.98 (ddd, 1H, *J*_1_ = 3.55 Hz, *J*_2_ = 7.20 Hz, C^4′^H(Rib)), 4.17 (dd, 1H, *J*_1_ = 4.00 Hz, *J*_2_ = 8.25 Hz, C^3′^H(Rib)), 4.56 (dd, 1H, *J*_1_ = 5.35 Hz, *J*_2_ = 10.65 Hz, C^2‘^H(Rib)), 5.08 (d, 1H, *J* = 1.70 Hz, C^3′^HO**H**(Rib)), 5.37 (m, 1H, C^5′^H_2_O**H**(Rib)) 5.47 (d, 1H, *J* = 4.60 Hz, C^2′^HO**H**(Rib)), 5.90 (d, 1H, *J* = 5.80 Hz, C^1′^H(Rib)), 7.10 (t, *J* = 7.30 Hz, 1H, Ph), 7.36 (t, *J* = 7.60 Hz, 2H, Ph), 7.83 (d, *J* = 7.95 Hz, 2H, Ph), 8.56 (s, 1H, C^8^H), 10.34 (s, 1H, C^6^N**H**Ph) ppm. ^13^C NMR (DMSO-*d*_6_, 125 MHz): *δ* 61.3 (CH(Rib)), 70.3 (CH(Rib)), 73.8 (CH(Rib)), 85.7 (CH(Rib)), 87.5 (CH(Rib)), 119.2 (CH(Ph)), 121.4 (CH(Ph)), 123.6 (CH(Ph)), 128.5 (CH(Ph)), 138.7 (C(Ph)), 140.7 (C), 150.5 (C), 152.5 (C) ppm. IR (KBr): 3336 (bm), 2926 (m), 1627 (s), 1584 (s), 1498 (s), 1458 (m), 1351 (m), 1318 (s), 1233 (m), 1121 (m), 1082 (m), 1048 (m), 749 (m), 691 (m), 631 (m) cm^−1^. ESI-MS (pos.) *m*/*z* (%): 416.0 [M(^35^Cl) + K^+^]^+^ (21), 400.0 [M(^35^Cl) + Na^+^]^+^ (100), 378.1 [M(^35^Cl) + H^+^]^+^ (92). ESI-MS (neg.) *m*/*z* (%): 753.0 [2·M(^35^Cl) − H^+^]^−^ (27), 411.9 [M(^35^Cl) + Cl^−^]^−^ (54), 375.9 [M(^35^Cl) − H^+^]^−^ (100). Anal. Cald for C_16_H_16_ClN_5_O_4_: C 50.87; H 4.27; N 18.54. Found: C 51.14; H 4.06; N 18.93.

(1-Adamantyl){4-[(2-chloro-9-β-d-ribofuranos-1-yl-9*H*-purin-6-yl)amino]phenyl}methanone (**20**)

Prepared from compound **14** (260 mg, 0.30 mmol), 1 M sodium methoxide (0.06 mmol). Purified using column chromatography (system c). Colorless crystalline powder, yield 149 mg (91%), mp 130–138 °C, R_f_ = 0.34 (system c). ^1^H NMR (DMSO-*d*_6_, 500 MHz): *δ* 1.70–1.76 (m, 6H, CH_2_(Ad)), 1.98 (m, 6H, CH_2_(Ad)), 2.03 (m, 3H, CH(Ad)), 3.56–3.61 (m, 1H, C^5′^H(Rib)), 3.67–3.71 (m, 1H, C^5′^H(Rib)), 3.99 (ddd, 1H, *J*_1_ = 3.85 Hz, *J*_2_ = 7.65 Hz, C^4′^H(Rib)), 4.17 (dd, 1H, *J*_1_ = 4.90 Hz, *J*_2_ = 8.55 Hz, C^3′^H(Rib)), 4.56 (dd, 1H, *J*_1_ = 5.65 Hz, *J*_2_ = 10.80 Hz, C^2′^H(Rib)), 5.04 (dd, 1H, *J*_1_ = 5.65 Hz, *J*_2_ = 11.1 Hz, C^5′^H_2_O**H**(Rib)), 5.21 (d, 1H, *J* = 5,05 Hz, C^3′^HO**H**(Rib)), 5.51 (d, 1H, *J* = 4.45 Hz, C^2′^HO**H**(Rib)), 5.92 (d, 1H, *J* = 5.20 Hz, C^1′^H(Rib)), 7.75 (d, 2H, *J* = 7.60 Hz, Ph), 7.96 (d, 2H, *J* = 7.65 Hz, Ph), 8.61 (s, 1H, C^8^H), 10.61 (s, 1H, C^6^N**H**Ph) ppm. ^13^C NMR (DMSO-*d*_6_, 125 MHz): *δ* 27.6 (CH_2_(Ad)), 36.0 (CH(Ad)), 38.7 (CH_2_(Ad)), 46.0 (C(Ad)), 61.2 (CH(Rib)), 70.2 (CH(Rib)), 73.8 (CH(Rib)), 85.7 (CH(Rib)), 87.5 (CH(Rib)), 119.5 (CH(Ph)), 119.9 (CH(Ph)), 128.6 (CH(Ph)), 132.8 (C(Ph)), 141.2 (C), 150.8 (C), 152.1 (C), 152.2 (C), 206.5 (Ph**C**OAd) ppm. IR (KBr): 3349 (bs), 2906 (s), 2851 (s), 1629 (s), 1606 (s), 1574 (s), 1508 (m), 1459 (s), 1321 (m), 1237 (m), 1175 (m), 1125 (m), 1084 (m), 987 (w), 750 (m) cm^−1^. ESI-MS (pos.) *m*/*z* (%): 1101.4 [2·M(^35^Cl) + Na^+^]^+^ (13), 578.2 [M(^35^Cl) + K^+^]^+^ (49), 562.2 [M(^35^Cl) + Na^+^]^+^ (100), 540.2 [M(^35^Cl) + H^+^]^+^ (72). ESI-MS (neg.) *m*/*z* (%): 574.0 [M(^35^Cl)+Cl^−^]^−^ (42), 538.1 [M(^35^Cl) − H^+^]^−^ (80), 406.0 [M(^35^Cl) − H^+^ − C_5_H_8_O_4_]^−^ (100). Anal. Cald for C_27_H_30_ClN_5_O_5_: C 60.05; H 5.60; N 12.97. Found: C 59.86; H 5.81; N 12.72.

(1-Adamantyl){3-[(2-chloro-9-β-d-ribofuranos-1-yl-9*H*-purin-6-yl)amino]phenyl}methanone (**21**)

Prepared from compound **15** (282 mg, 0.33 mmol), 1 M sodium methoxide (0.07 mmol). Purified using column chromatography (system c). Colorless crystalline powder, yield 155 mg (87%), mp 144–151 °C, R_f_ = 0.36 (system c). ^1^H NMR (DMSO-*d*_6_, 500 MHz): *δ* 1.69–1.74 (m, 6H, CH_2_(Ad)), 1.99 (m, 6H, CH_2_(Ad)), 2.04 (m, 3H, CH(Ad)), 3.57–3.61 (m, 1H, C^5′^H(Rib)), 3.67–3.72 (m, 1H, C^5′^H(Rib)), 3.98 (ddd, 1H, *J*_1_ = 3.85 Hz, *J*_2_ = 7.50 Hz, C^4′^H(Rib)), 4.17 (dd, 1H, *J*_1_ = 4.55 Hz, *J*_2_ = 8.55 Hz, C^3′^H(Rib)), 4.56 (dd, 1H, *J*_1_ = 5.50 Hz, *J*_2_ = 10.95 Hz, C^2′^H(Rib)), 5.04 (dd, 1H, *J*_1_ = 5.65 Hz, *J*_2_ = 11.15 Hz, C^5′^H_2_O**H**(Rib)), 5.21 (d, 1H, *J* = 5.05 Hz, C^3′^HO**H**(Rib)), 5.50 (d, 1H, *J* = 6.10 Hz, C^2′^HO**H**(Rib)), 5.91 (d, 1H, *J* = 5.80 Hz, C^1′^H(Rib)), 7.23 (d, 1H, *J* = 7.80 Hz, Ph), 7.44 (t, 1H, *J* = 7.93 Hz, Ph), 7.91 (d, 1H, *J* = 8.40 Hz, Ph), 8.32 (s, 1H, Ph), 8.59 (s, 1H, C^8^H), 10.54 (s, 1H, C^6^N**H**Ph) ppm. ^13^C NMR (DMSO-*d*_6_, 125 MHz): *δ* 27.5 (CH_2_(Ad)), 35.9 (CH(Ad)), 38.5 (CH_2_(Ad)), 46.1 (C(Ad)), 61.2 (CH(Rib)), 70.3 (CH(Rib)), 73.8 (CH(Rib)), 85.7 (CH(Rib)), 87.5 (CH(Rib)), 119.3 (CH(Ph)), 119.6 (CH(Ph)), 122.4 (CH(Ph)), 122.9 (CH(Ph)), 128.6 (C(Ph)), 138.0 (C(Ph)), 138.8 (C), 141.0 (C), 150.6 (C), 152.3 (C), 208.2 (Ph**C**OAd) ppm. IR (KBr): 3355 (bs), 2906 (s), 2852 (m), 1626 (s), 1601 (s), 1574 (s), 1454 (m), 1319 (s), 1273 (m), 1227 (m), 1174 (w), 1120 (m), 1084 (m), 1051 (m), 754 (m) cm^−1^. ESI-MS (pos.) *m*/*z* (%): 578.2 [M(^35^Cl) + K^+^]^+^ (10), 562.2 [M(^35^Cl) + Na^+^]^+^ (27), 540.2 [M(^35^Cl) + H^+^]^+^ (100). ESI-MS (neg.) *m*/*z* (%): 1077.2 [2·M(^35^Cl) − H^+^]^−^ (12), 574.1 [M(^35^Cl) + Cl^−^]^−^ (10), 538.1 [M(^35^Cl) − H^+^]^−^ (100). Anal. Cald for C_27_H_30_ClN_5_O_5_: C 60.05; H 5.60; N 12.97. Found: C 60.03; H 5.66; N 12.85.

### 3.6. General Procedure for the Reduction of Compounds **17**, **18**, **20**, and **21** with NaBH_4_

Reduction of compounds **17**, **18**, **20**, and **21** was performed according to a slightly modified version of a previously published procedure [39]. The corresponding ketone (0.12–0.17 mmol) was dissolved in ethanol (5 cm^3^) and the mixture was cooled in an ice bath. After this, sodium borohydride (1.23 equiv.) was added into the mixture in one portion at 0 °C. The reaction mixture was vigorously stirred at room temperature till the TLC indicated the consumption of all starting material (24 h). After this, the reaction was quenched by the addition of 1 M HCl (6 cm^3^) and the formation of a colorless precipitation was observed. The precipitate was filtrated with suction and the solid material was washed several times with distilled water. The desired compound was obtained after purification of the crude product using column chromatography.

(1-Adamantyl){4-[(9-β-d-ribofuranos-1-yl-9*H*-purin-6-yl)amino]phenyl}methanol (**22**)

Prepared from compound **17** (61 mg, 0.12 mmol), sodium borohydride (0.15 mmol). Purified using column chromatography (system b). Colorless crystalline powder, yield 51 mg (82%), mp 145–156 °C, R_f_ = 0.43 (system b). ^1^H NMR (DMSO-*d*_6_, 500 MHz): *δ* 1.40–1.42 (m, 3H, CH_2_(Ad)), 1.51–1.53 (m, 3H, CH_2_(Ad)), 1.59–1.63 (m, 6H, CH_2_(Ad)), 1.91 (m, 3H, CH(Ad)), 3.56–3.60 (m, 1H, C^5′^H(Rib)), 3.68–3.72 (m, 1H, C^5′^H(Rib)), 3.99 (ddd, 1H, *J*_1_ = 3.44 Hz, *J*_2_ = 6.79 Hz, C^4′^H(Rib)), 4.02 (d, 1H, *J* = 3.81 Hz, PhC**H**OHAd), 4.18 (dd, 1H, *J*_1_ = 4.43 Hz, *J*_2_ = 8.09 Hz, C^3′^H(Rib)), 4.64 (dd, 1H, *J_1_* = 5.65 Hz, *J_2_* = 11.29 Hz, C^2′^H(Rib)), 4.93 (d, 1H, *J* = 4.12 Hz, PhCHO**H**Ad)), 5.18 (d, 1H, *J* = 4.58 Hz, C^3′^HO**H**(Rib)), 5.27 (dd, 1H, *J*_1_ = 5.49 Hz, *J*_2_ = 10.99 Hz, C^5′^H_2_O**H**(Rib)), 5.45 (d, 1H, *J* = 6.10 Hz, C^2′^HO**H**(Rib)), 5.96 (d, 1H, *J* = 5.95 Hz, C^1′^H(Rib)), 7.17 (d, 2H, *J* = 8.39 Hz, Ph), 7.82 (d, 2H, *J* = 8.39 Hz, Ph), 8.38 (s, 1H, C^2^H), 8.52 (s, 1H, C^8^H), 9.86 (s, 1H, C^6^N**H**Ph) ppm. ^13^C NMR (DMSO-*d*_6_, 125 MHz): *δ* 27.8 (CH_2_(Ad)), 36.7 (CH(Ad)), 36.8 (CH_2_(Ad)), 37.9 (C(Ad)), 61.5 (CH(Rib)), 70.5 (CH(Rib)), 73.6 (CH(Rib)), 80.5 (Ph**C**HOHAd), 85.8 (CH(Rib)), 87.8 (CH(Rib)), 119.9 (CH(Ph)), 120.3 (CH(Ph)), 127.7 (CH(Ph)), 136.9 (C(Ph)), 137.8 (C(Ph)), 140.5 (C), 149.2 (C), 151.9 (C), 152.2 (C) ppm. IR (KBr): 3354 (bs), 2904 (s), 2848 (s), 1627 (s), 1586 (s), 1511 (s), 1478 (s), 1420 (m), 1374 (m), 1296 (m), 1234 (m), 1125 (m), 1084 (m), 1029 (m), 742 (m) cm^−1^. ESI-MS (pos.) *m*/*z* (%): 508.3 [M + H^+^]^+^ (100). ESI-MS (neg.) *m*/*z* (%): 1013.4 [2·M − H^+^]^−^ (16), 542.1 [M + Cl^−^]^−^ (21), 506.2 [M − H^+^]^−^ (100). Anal. Cald for C_27_H_33_N_5_O_5_: C 63.89; H 6.55; N 13.80. Found: C 63.63; H 6.36; N 13.91.

(1-Adamantyl){3-[(9-β-d-ribofuranos-1-yl-9*H*-purin-6-yl)amino]phenyl}methanol (**23**)

Prepared from compound **18** (59 mg, 0.12 mmol), sodium borohydride (0.15 mmol). Purified using column chromatography (system b). Colorless crystalline powder, yield 53 mg (90%), mp 130–145 °C, R_f_ = 0.52 (system b). ^1^H NMR (DMSO-*d*_6_, 500 MHz): *δ* 1.42–1.45 (m, 3H, CH_2_(Ad)), 1.51–1.53 (m, 3H, CH_2_(Ad)), 1.61–1.64 (m, 6H, CH_2_(Ad)), 1.91 (m, 3H, CH(Ad)), 3.56–3.60 (m, 1H, C^5′^H(Rib)), 3.68–3.72 (m, 1H, C^5′^H(Rib)), 3.98 (ddd, 1H, *J*_1_ = 3.51 Hz, *J*_2_ = 7.02 Hz, C^4′^H(Rib)), 4.02 (d, 1H, *J* = 3.97 Hz, PhC**H**OHAd), 4.18 (dd, 1H, *J*_1_ = 4.58 Hz, *J*_2_ = 8.09 Hz, C^3′^H(Rib)), 4.64 (dd, 1H, *J*_1_ = 5.95 Hz, *J*_2_ = 11.29 Hz, C^2′^H(Rib)), 5.00 (d, 1H, *J* = 3.97 Hz, PhCHO**H**Ad), 5.18 (d, 1H, *J* = 4.73 Hz, C^3′^HO**H**(Rib)), 5.26 (dd, 1H, *J*_1_ = 4.88 Hz, *J*_2_ = 6.56 Hz, C^5′^H_2_O**H**(Rib)), 5.46 (d, 1H, *J* = 6.10 Hz, C^2′^HO**H**(Rib)), 5.96 (d, 1H, *J* = 5.95 Hz, C^1′^H(Rib)), 6.93 (d, 1H, *J* = 7.63 Hz, Ph), 7.23 (t, 1H, *J* = 7.78 Hz, Ph), 7.70 (s, 1H, Ph), 7.84 (d, 1H, *J* = 9.00 Hz, Ph), 8.36 (s, 1H, C^2^H), 8.52 (s, 1H, C^8^H), 9.86 (s, 1H, C^6^N**H**Ph) ppm. ^13^C NMR (DMSO-*d*_6_, 125 MHz): *δ* 28.3 (CH_2_(Ad)), 37.2 (CH(Ad)), 37.3 (CH_2_(Ad)), 38.5 (C(Ad)), 62.0 (CH(Rib)), 71.0 (CH(Rib)), 74.1 (CH(Rib)), 81.5 (Ph**C**HOHAd), 86.3 (CH(Rib)), 88.3 (CH(Rib)), 119.9 (CH(Ph)), 120.8 (CH(Ph)), 121.5 (CH(Ph)), 123.1 (CH(Ph)), 127.3 (C(Ph)), 138.7 (C(Ph)), 141.0 (C), 143.4 (C), 149.8 (C), 152.3 (C), 152.7 (C) ppm. IR (KBr): 3330 (bs), 2904 (s), 2846 (m), 1628 (s), 1581 (s), 1477 (s), 1375 (m), 1304 (m), 1225 (m), 1111 (m), 1084 (m), 1043 (m), 795 (m), 731 (m), 644 (m) cm^−1^. ESI-MS (pos.) *m*/*z* (%): 546.2 [M + K^+^]^+^ (13), 530.3 [M + Na^+^]^+^ (16), 508.3 [M + H^+^]^+^ (100). ESI-MS (neg.) *m*/*z* (%): 1049.3 [2·M + Cl^−^]^−^ (7), 1013.4 [2·M − H^+^]^−^ (6), 542.1 [M + Cl^−^]^−^ (71), 506.2 [M − H^+^]^−^ (100). Anal. Cald for C_27_H_33_N_5_O_5_: C 63.89; H 6.55; N 13.80. Found: C 63.33; H 6.74; N 14.05.

(1-Adamantyl){4-[(2-chloro-9-β-d-ribofuranos-1-yl-9*H*-purin-6-yl)amino]phenyl}methanol (**24**)

Prepared from compound **20** (92 mg, 0.17 mmol), sodium borohydride (0.21 mmol). Purified using column chromatography (system c). Colorless crystalline powder, yield 79 mg (86%), mp 138–142 °C, R_f_ = 0.28 (system c). ^1^H NMR (DMSO-*d*_6_, 500 MHz): *δ* 1.40–1.43 (m, 3H, CH_2_(Ad)), 1.51–1.53 (m, 3H, CH_2_(Ad)), 1.59–1.63 (m, 6H, CH_2_(Ad)), 1.91 (m, 3H, CH(Ad)), 3.57–3.59 (m, 1H, C^5′^H(Rib)), 3.67–3.70 (m, 1H, C^5′^H(Rib)), 3.97 (ddd, 1H, *J*_1_ = 3.66 Hz, *J*_2_ = 7.32 Hz, C^4′^H(Rib)), 4.03 (d, 1H, *J* = 3.05 Hz, PhC**H**OHAd), 4.16 (dd, 1H, *J*_1_ = 3.81 Hz, *J*_2_ = 7.93 Hz, C^3′^H(Rib)), 4.55 (dd, 1H, *J*_1_ = 5.19 Hz, *J*_2_ = 10.68 Hz, C^2′^H (Rib)), 4.96 (d, 1H, *J* = 3.81 Hz, PhCHO**H**Ad)), 5.04 (d, 1H, C^3′^HO**H**(Rib)), 5.22 (dd, 1H, C^5′^H_2_O**H**(Rib)) 5.51 (d, 1H, C^2′^HO**H**(Rib)), 5.89 (d, 1H, *J* = 5.80 Hz, C^1′^H(Rib)), 7.20 (d, 2H, *J* = 8.39 Hz, Ph), 7.73 (d, 2H, *J* = 8.39 Hz, Ph), 8.54 (s, 1H, C^8^H), 10.29 (s, 1H, C^6^N**H**Ph) ppm. ^13^C NMR (DMSO-*d*_6_, 125 MHz): *δ* 28.3 (CH_2_(Ad)), 37.2 (CH(Ad)), 37.3 (CH_2_(Ad)), 38.4 (C(Ad)), 61.8 (CH(Rib)), 70.8 (CH(Rib)), 74.3 (CH(Rib)), 80.9 (Ph**C**HOHAd), 86.2 (CH(Rib)), 87.9 (CH(Rib)), 119.6 (CH(Ph)), 120.6 (CH(Ph)), 128.3 (CH(Ph)), 137.5 (C(Ph)), 138.4 (C), 141.2 (C), 151.0 (C), 152.9 (C), 153.0 (C) ppm. IR (KBr): 3387 (bs), 2905 (s), 2848 (s), 1626 (s), 1582 (s), 1511 (m), 1465 (m), 1348 (m), 1317 (s), 1233 (m), 1122 (m), 1084 (m), 1048 (m), 756 (w), 632 (m) cm^−1^. ESI-MS (pos.) *m*/*z* (%): 580.2 [M(^35^Cl) + K^+^]^+^ (19), 564.2 [M(^35^Cl) + Na^+^]^+^ (100), 542.2 [M(^35^Cl) + H^+^]^+^ (90). ESI-MS (neg.) *m*/*z* (%): 1117.2 [2·M(^35^Cl) − Cl^−^]^−^ (12), 1081.2 [2·M(^35^Cl) − H^+^]^−^ (7), 576.2 [M(^35^Cl) + Cl^−^]^−^ (100), 540.1 [M(^35^Cl) − H^+^]^−^ (84). Anal. Cald for C_27_H_32_ClN_5_O_5_: C 59.83; H 5.95; N 12.92. Found: C 59.45; H 5.61; N 12.54.

(1-Adamantyl){3-[(2-chloro-9-β-d-ribofuranos-1-yl-9*H*-purin-6-yl)amino]phenyl}methanol (**25**)

Prepared from compound **21** (90 mg, 0.17 mmol), sodium borohydride (0.21 mmol). Purified using column chromatography (system c). Colorless crystalline powder, yield 73 mg (80%), mp 170–174 °C, R_f_ = 0.22 (system c). ^1^H NMR (DMSO-*d*_6_, 500 MHz): *δ* 1.44–1.46 (m, 3H, CH_2_(Ad)), 1.52–1.54 (m, 3H, CH_2_(Ad)), 1.61–1.66 (m, 6H, CH_2_(Ad)), 1.92 (m, 3H, CH(Ad)), 3.55–3.60 (m, 1H, C^5′^H(Rib)), 3.66–3.70 (m, 1H, C^5′^H(Rib)), 3.97 (ddd, 1H, *J*_1_ = 3.81 Hz, *J*_2_ = 7.48 Hz, C^4′^H(Rib)), 4.02 (d, 1H, *J* = 3.97 Hz, PhC**H**OHAd), 4.16 (dd, 1H, *J*_1_ = 4.73 Hz, *J*_2_ = 8.54 Hz, C^3′^H(Rib)), 4.54 (dd, 1H, *J*_1_ = 5.49 Hz, *J*_2_ = 10.83 Hz, C^2′^H(Rib)), 5.01 (d, 1H, *J* = 4.12 Hz, PhCHO**H**Ad), 5.05 (d, 1H, *J* = 5.65 Hz, C^3′^H_2_O**H**(Rib)), 5.20 (dd, 1H, *J* = 5.04 Hz, C^5′^HO**H**(Rib)), 5.50 (d, 1H, *J* = 6.10 Hz, C^2′^HO**H**(Rib)), 5.89 (d, 1H, *J* = 5.80 Hz, C^1′^H(Rib)), 6.98 (d, 1H, *J* = 7.63 Hz, CH(Ph)), 7.26 (t, 1H, *J* = 7.78 Hz, Ph), 7.59 (d, 1H, *J* = 7.93 Hz, Ph), 7.67 (s, 1H, Ph), 8.54 (s, 1H, C^8^H), 10.31 (s, 1H, C^6^N**H**Ph) ppm. ^13^C NMR (DMSO-*d*_6_, 125 MHz): *δ* 27.8 (CH_2_(Ad)), 36.7 (CH(Ad)), 36.8 (CH_2_(Ad)), 37.9 (C(Ad)), 61.2 (CH(Rib)), 70.3 (CH(Rib)), 73.8 (CH(Rib)), 80.9 (Ph**C**HOHAd), 85.7 (CH(Rib)), 87.4 (CH(Rib)), 119.1 (CH(Ph)), 119.9 (CH(Ph)), 121.8 (CH(Ph)), 123.5 (CH(Ph)), 126.9 (C(Ph)), 137.2 (C(Ph)), 140.6 (C), 142.9 (C), 150.5 (C), 152.3 (C), 152.6 (C) ppm. IR (KBr): 3346 (bs), 2904 (s), 2849 (m), 1627 (s), 1578 (s), 1459 (m), 1352 (m), 1317 (m), 1222 (m), 1125 (m), 1086 (m), 1046 (m), 787 (w), 730 (m), 632 (m) cm^−1^. ESI-MS (pos.) *m*/*z* (%): 1121.4 [2·M(^35^Cl) + K^+^]^+^ (13), 1105.4 [2·M(^35^Cl) + Na^+^]^+^ (25), 1083.3 [2·M(^35^Cl) + H^+^]^+^ (7), 580.2 [M(^35^Cl) + K^+^]^+^ (37), 564.2 [M(^35^Cl) + Na^+^]^+^ (61), 542.2 [M(^35^Cl) + H^+^]^+^ (100). ESI-MS (neg.) *m*/*z* (%): 1081.3 [2·M(^35^Cl) − H^+^]^−^ (8), 576.1 [M(^35^Cl) + Cl^−^]^−^ (22), 540.1 [M(^35^Cl) − H^+^]^−^ (100). Anal. Cald for C_27_H_32_ClN_5_O_5_: C 59.83; H 5.95; N 12.92. Found: C 59.56; H 6.25; N 12.68.

### 3.7. Proliferation Assay

The cell lines K562 and MV4;11 were obtained from the European Collection of Cell Cultures. The cell lines were cultivated in Dulbecco’s modified Eagle’s medium supplemented with 10% fetal bovine serum, penicillin (100 U/mL), and streptomycin (100 μg/mL) at 37 °C in 5% CO_2_. For the assays, cells seeded into 96 well plates (5000 cells per well) were treated in triplicate, with six different doses of each compound. After 72 h, a resazurin (Sigma-Aldrich, St. Louis, MO, USA) solution was added for four hours, and the fluorescence of resorufin formed in the live cells was measured at 544 nm/590 nm (excitation/emission) using a Fluoroskan Ascent microplate reader (Labsystems). The IC_50_ value, the drug concentration reducing the number of cells to 50%, was calculated from the dose–response curves.

## 4. Conclusions

Purine nucleosides represent one of the most important substrates in living systems, and they continue to fascinate researchers with their nearly unlimited allotropy and potential to extend their biological effects. According to a previously published study dealing with the antiproliferative activity of natural cytokinins, including topolin ribosides, we designed and prepared a novel series of 2,6-disubstituted and 2,6,9-trisubstituted purine ribonucleosides at the C6 adamantylated aromatic amines. In concert with the fact that the adamantane moiety can be immersed into the cavity of β-CD, we studied the ability of the newly prepared adamantane-based purine ribonucleosides to form host–guest complexes with β-CD. The formation of host–guest complexes with 1:1 stoichiometry was proven by the results obtained in NMR and ESI-MS experiments, respectively. Additionally, the antiproliferative activity of single purine ribonucleosides and their equimolar mixtures with β-CD against two types of tumor cell lines (MV4;11 and K562, respectively) was assayed. Most of the adamantylated purine nucleosides (except compounds **17** and **25**) displayed antiproliferative activity against one or both of the tested cell lines in the micromolar range (IC_50_ = 12.3–23.8 μM). The highest efficacy was shown by compounds **20** and **24**, respectively, with chlorine at C2 and the para-substituted aromatic ring at C6. Finally, we demonstrated that β-CD had only a slightly negative effect on the biological activity of the prepared adamantylated purine nucleosides, which was probably incurred due to competitive drug binding inside the cavity of β-CD.

## Figures and Tables

**Figure 3:**
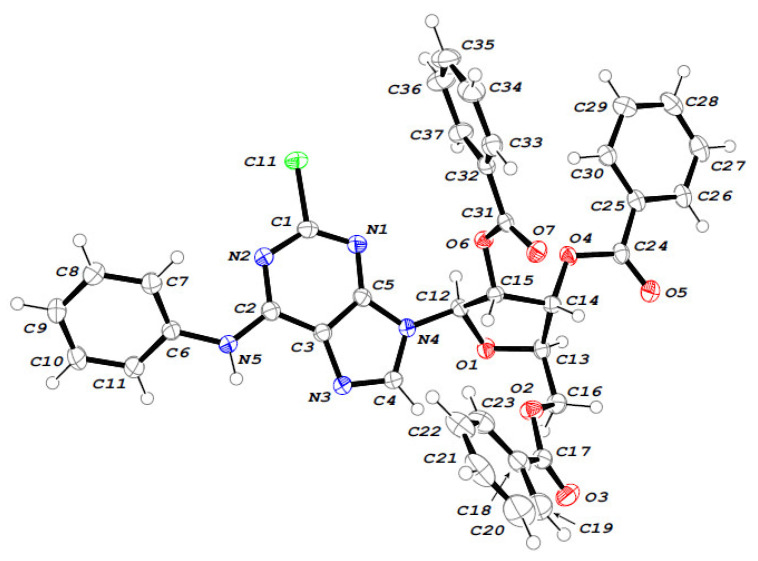
An X-ray diffraction analysis of purine 13. An ORTEP diagram was drawn with a 40% ellipsoid probability level.

**Table 1:** In vitro antiproliferative activity of purines **16**–**25** and their complexes with β-CD.

Compound	MV4;11 IC_50_ (μM) ^1^		K562 IC_50_ (μM) ^1^	
	Purine	Purine·β-CD (1:1)	Purine	Purine·β-CD (1:1)
**16**	>25	>25	>25	>25
**17**	>25	>25	>25	>25
**18**	21.8 ± 5.6	24.6 ± 0.7	>25	>25
**19**	>25	>25	>25	>25
**20**	16.6 ± 7.6	21.2 ± 5.4	21.4 ± 2.3	22.7 ± 3.2
**21**	23.8 ± 1.7	24.1 ± 1.3	>25	>25
**22**	20.0 ± 7.1	24.4 ± 0.9	>25	>25
**23**	18.8 ± 8.8	25.0 ± 0.0	>25	>25
**24**	12.3 ± 3.9	18.5 ± 7.8	23.4 ± 0.4	24.4 ± 0.8
**25**	>25	>25	>25	>25

^1^ IC_50_ values are expressed as the mean ± SD of duplicated experiments. Tumor cell lines: MV4;11, human acute myelogenous leukemia; K562, human chronic myelogenous leukemia.

## Data Availability

Not applicable.

## Supplementary Materials

The following are available online at https://www.mdpi.com/article/10.3390/ijms232315143/s1. Figures S1–S45: ^1^H and ^13^C NMR spectra of compounds **2**, **4**–**24**; Table S1: Crystal data and refinement parameters of compound **13**; Figure S46: crystal packing of compound **13**; CIF file of compound **13**.
