# Supplementary material for: Adamantane-Substituted Purine Nucleosides: Synthesis, Host–Guest Complexes with β-Cyclodextrin and Biological Activity
Source: Int J Mol Sci. 2022 Dec 1;23(23):15143. doi: 10.3390/ijms232315143 (PMC9739181; doi:10.3390/ijms232315143)
Supplement: Supplementary file 1 [file ijms-23-15143-s001.zip › ijms-2067232-supplementary.pdf]

Adamantane-Substituted Purine Nucleosides: Synthesis, Host–Guest Complexes with β-Cyclodextrin and Biological Activity

Jana Rudolfová [1], Vladimír Kryštof [2], Marek Nečas[3], Robert Vícha [1] and Michal Rouchal [1,*]

[1] *Department of Chemistry, Faculty of Technology, Tomas Bata University in Zlín, Vavrečkova 5669, 760 01 Zlín, Czech Republic*

[2] *Department of Experimental Biology, Palacký University, Šlechtitelů 27, 783 71 Olomouc, Czech Republic*

[3] *Department of Chemistry, Faculty of Science, Masaryk University, Kotlářská 2, 602 00 Brno, Czech Republic*

*Corresponding author: e-mail: rouchal@utb.cz; tel.: +420-576031432

Supporting Information

Table of Contents

^{1}H and ^{13}C NMR spectra of compounds **2, 4–25** S2

Crystal data and structure refinement of compound **13** S47

^{1}H and ^{13}C NMR spectra of compounds 2, 4–25

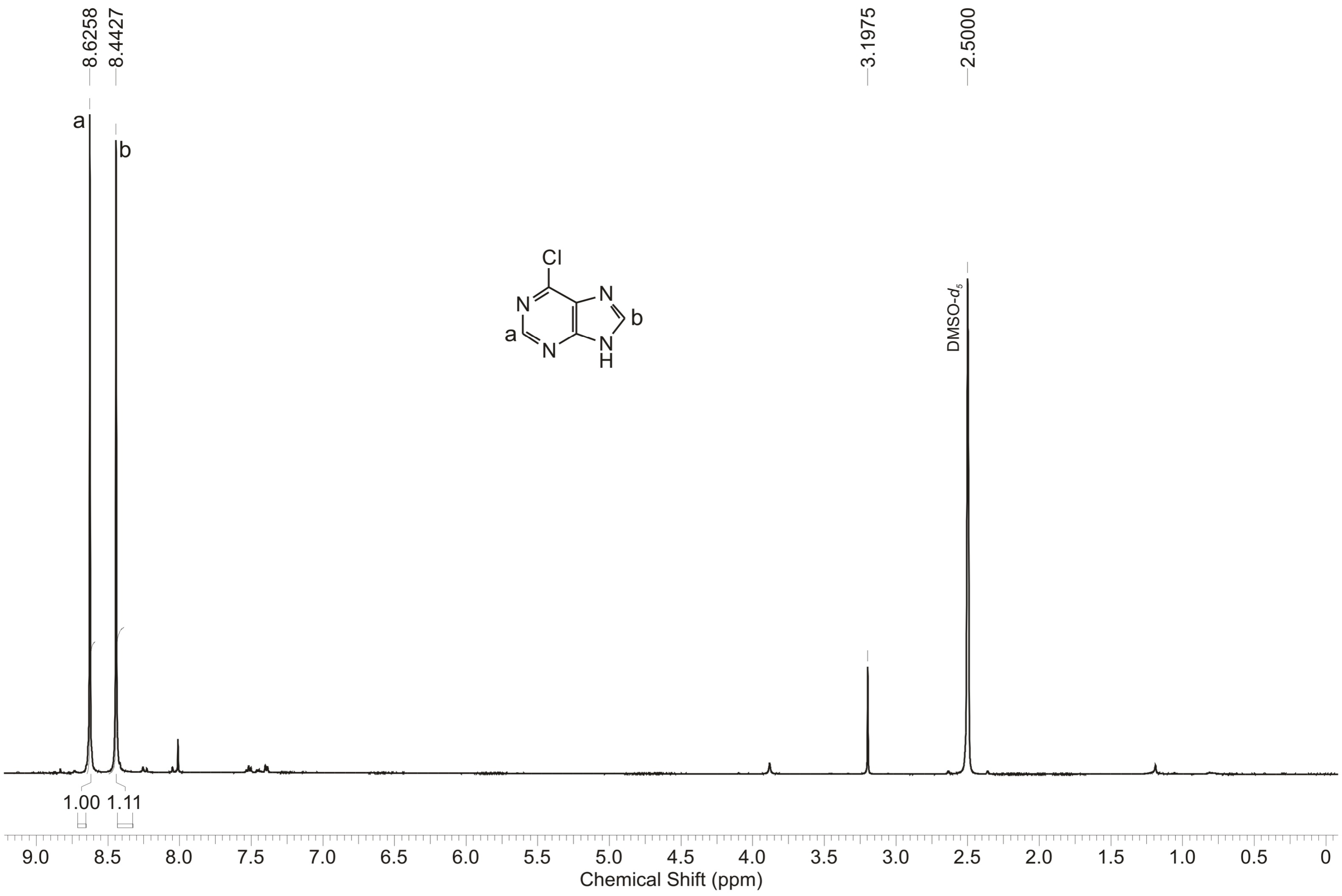

Figure S1. ^{1}H NMR spectrum (DMSO-d_6, 500 MHz, 303 K) of compound **2**.

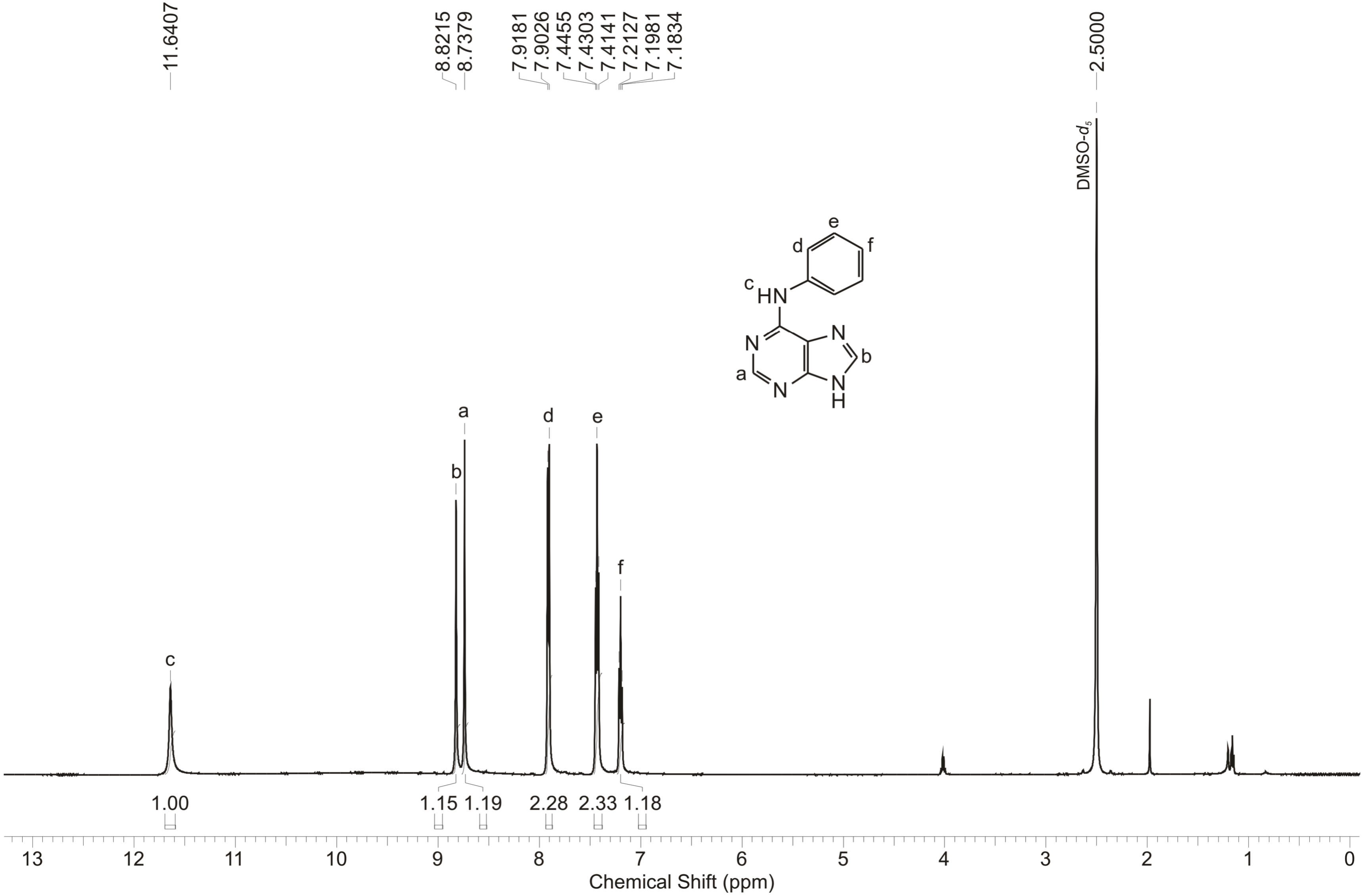

Figure S2. ^{1}H NMR spectrum (DMSO-d_6, 500 MHz, 303 K) of compound **4**.

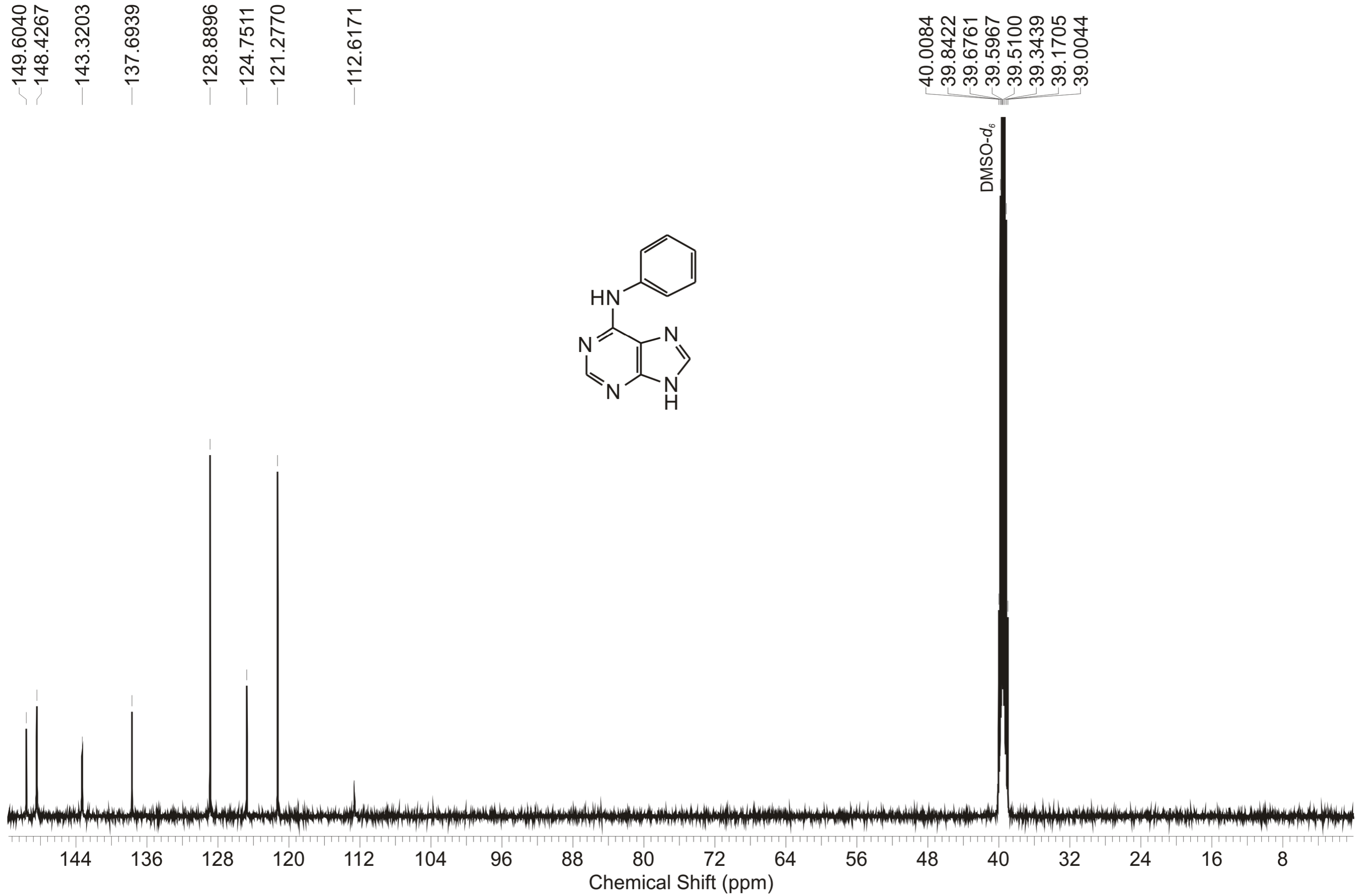

Figure S3. ^{13}C NMR spectrum (DMSO-d_6, 125 MHz, 303 K) of compound **4**.

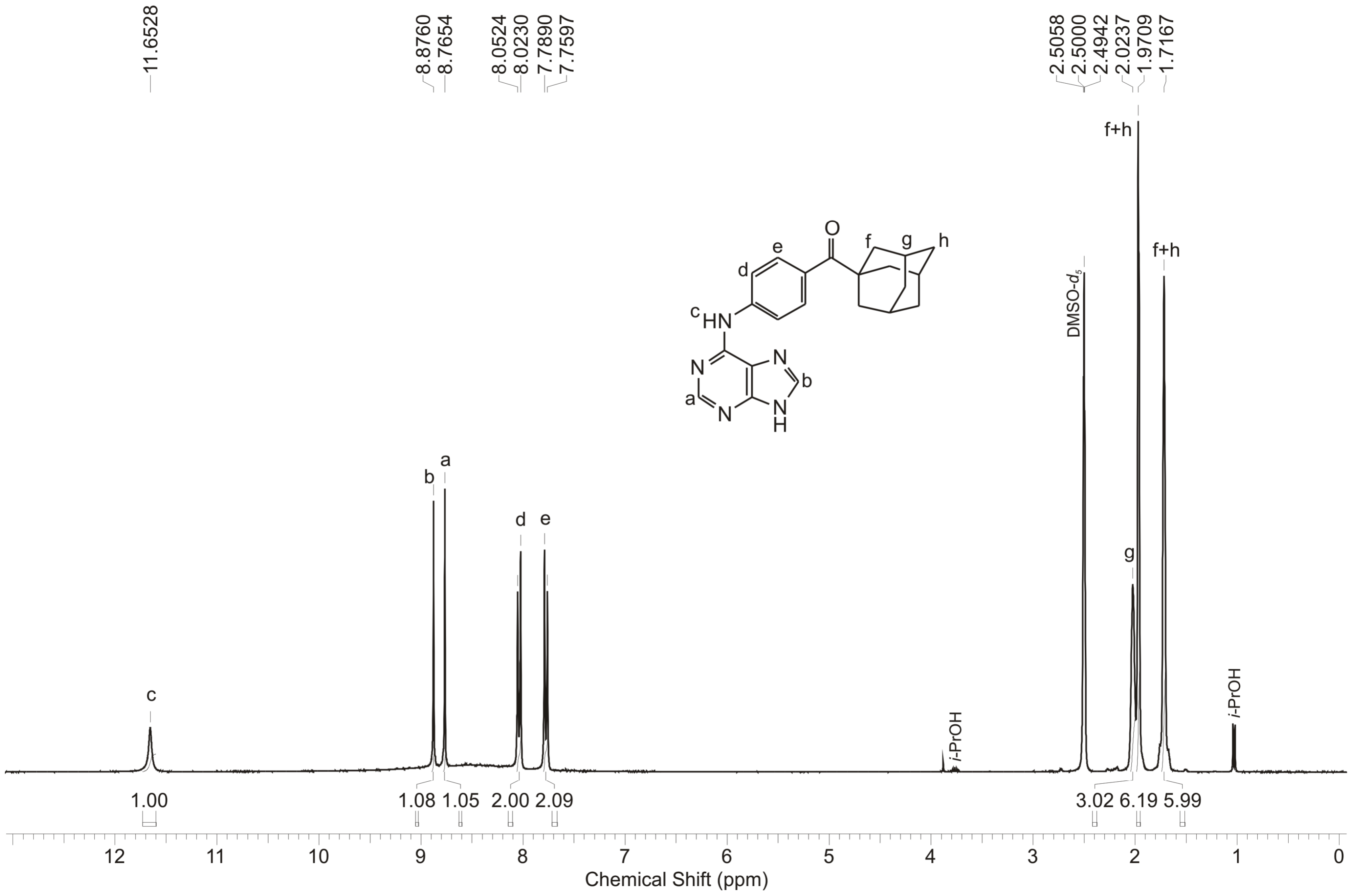

Figure S4. ^{1}H NMR spectrum (DMSO-d_6, 500 MHz, 303 K) of compound **5**.

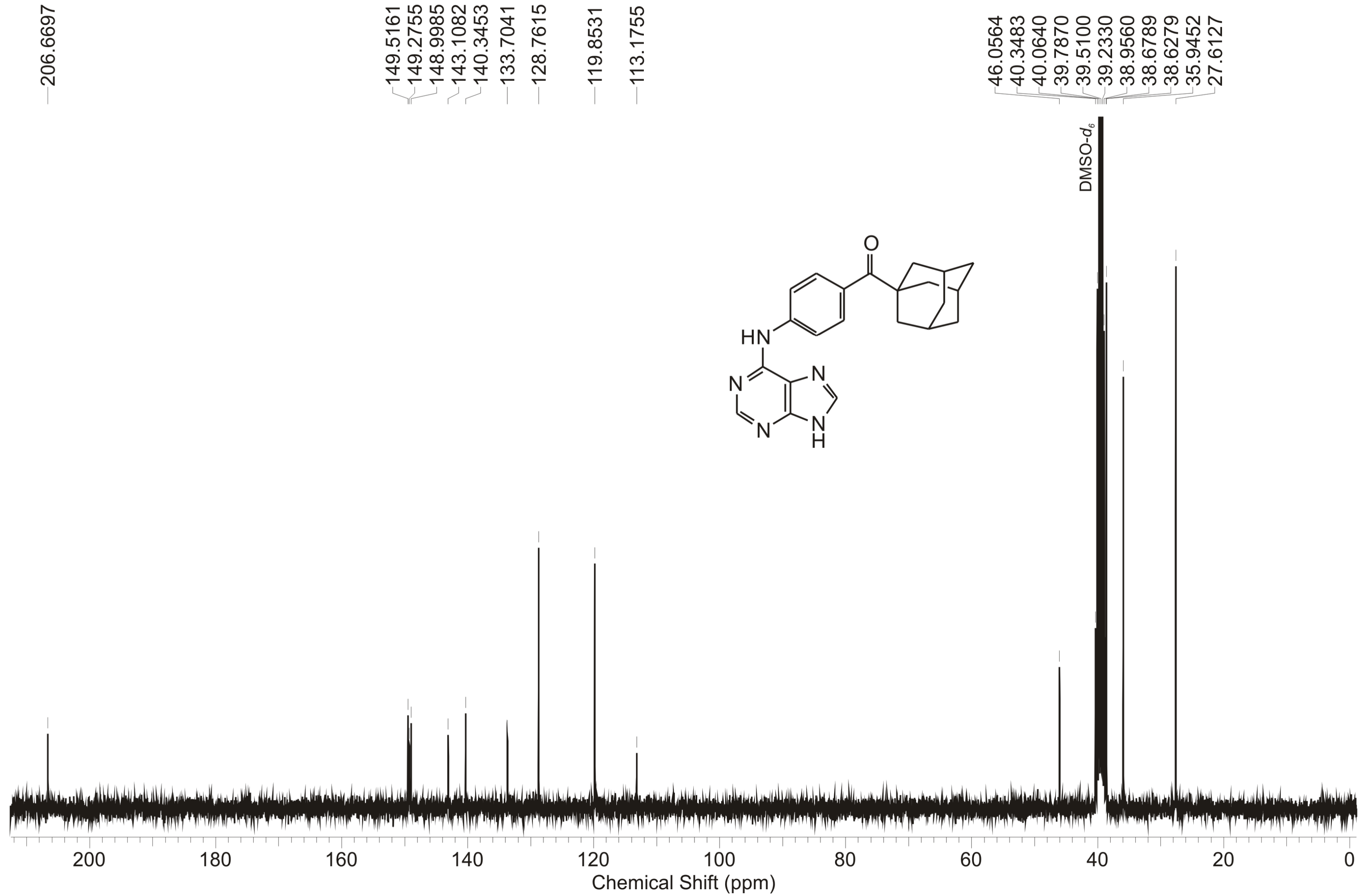

Figure S5. ^{13}C NMR spectrum (DMSO-d_6, 125 MHz, 303 K) of compound **5**.

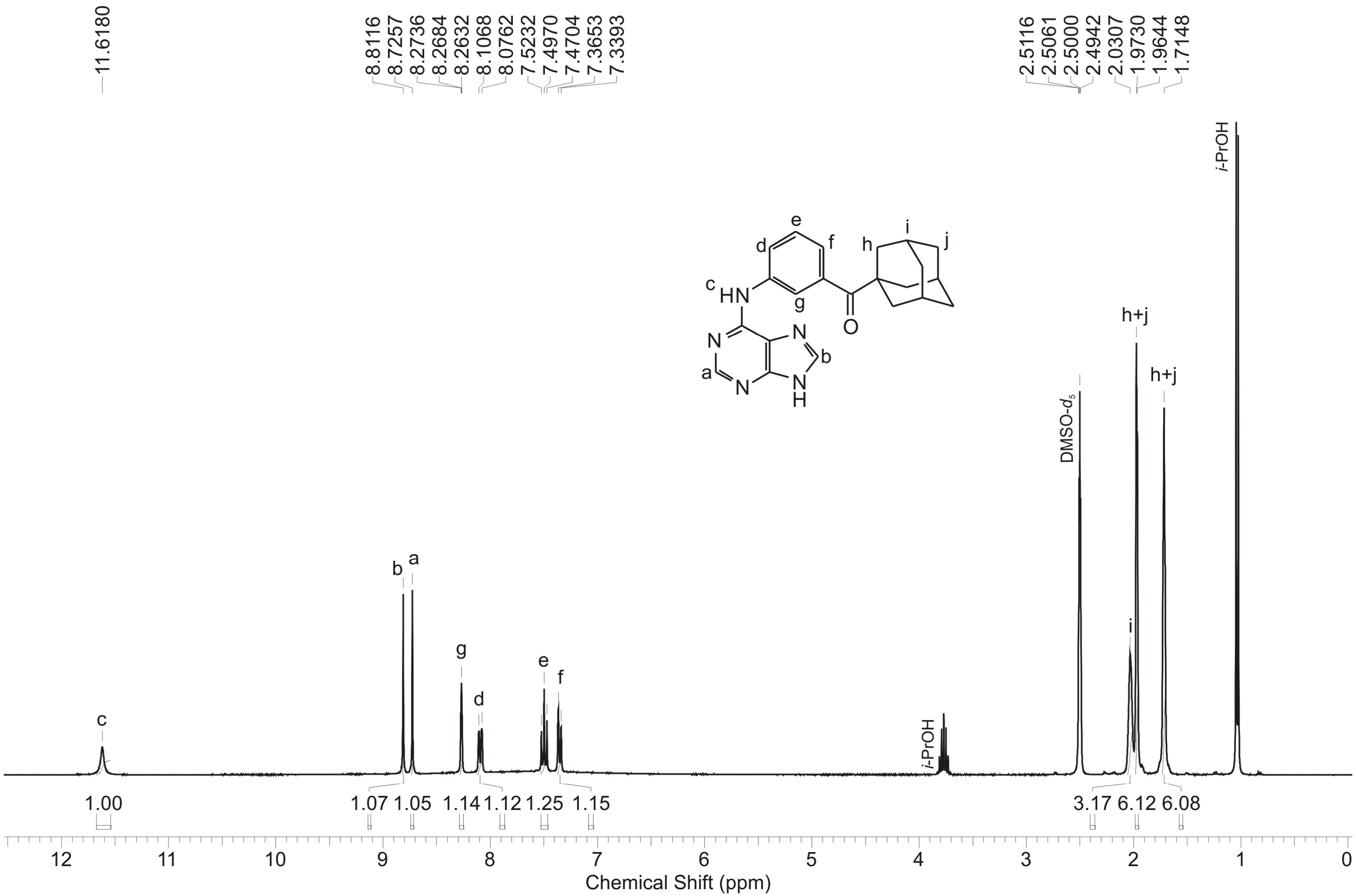

Figure S6. ^{1}H NMR spectrum (DMSO-d_6, 500 MHz, 303 K) of compound **6**.

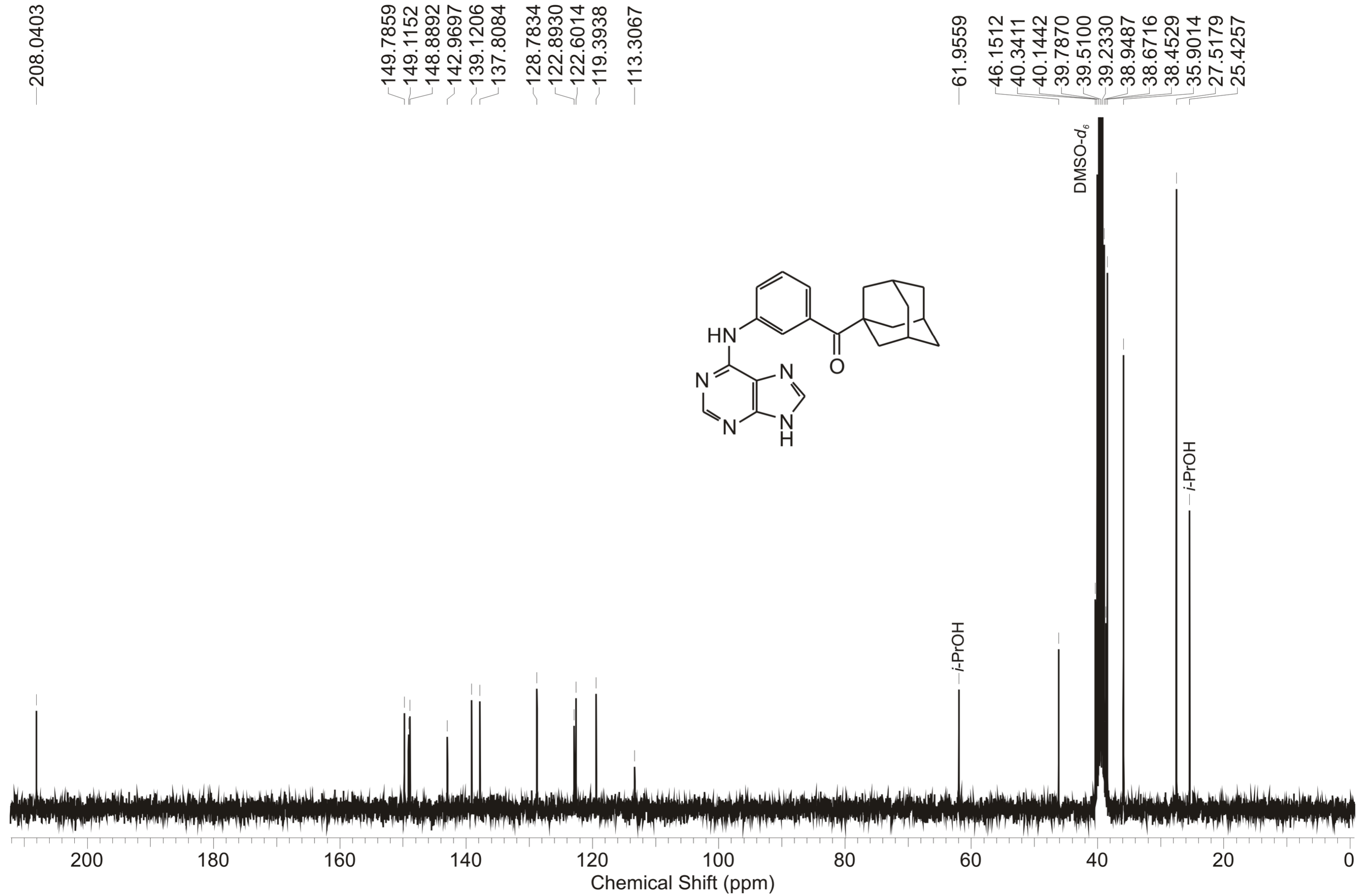

Figure S7. ^{13}C NMR spectrum (DMSO-d_6, 125 MHz, 303 K) of compound **6**.

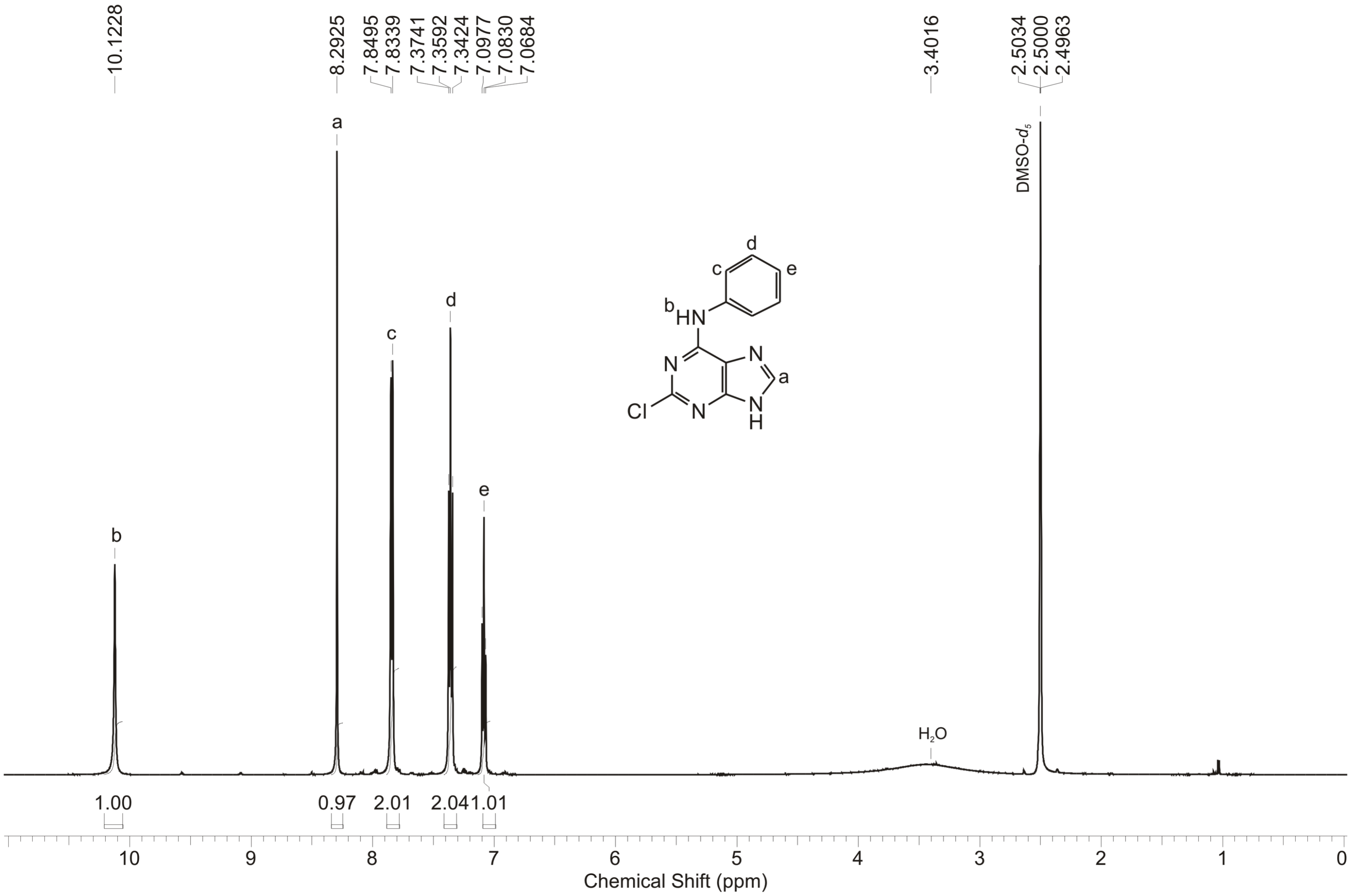

Figure S8. ^{1}H NMR spectrum (DMSO-d_6, 500 MHz, 303 K) of compound **7**.

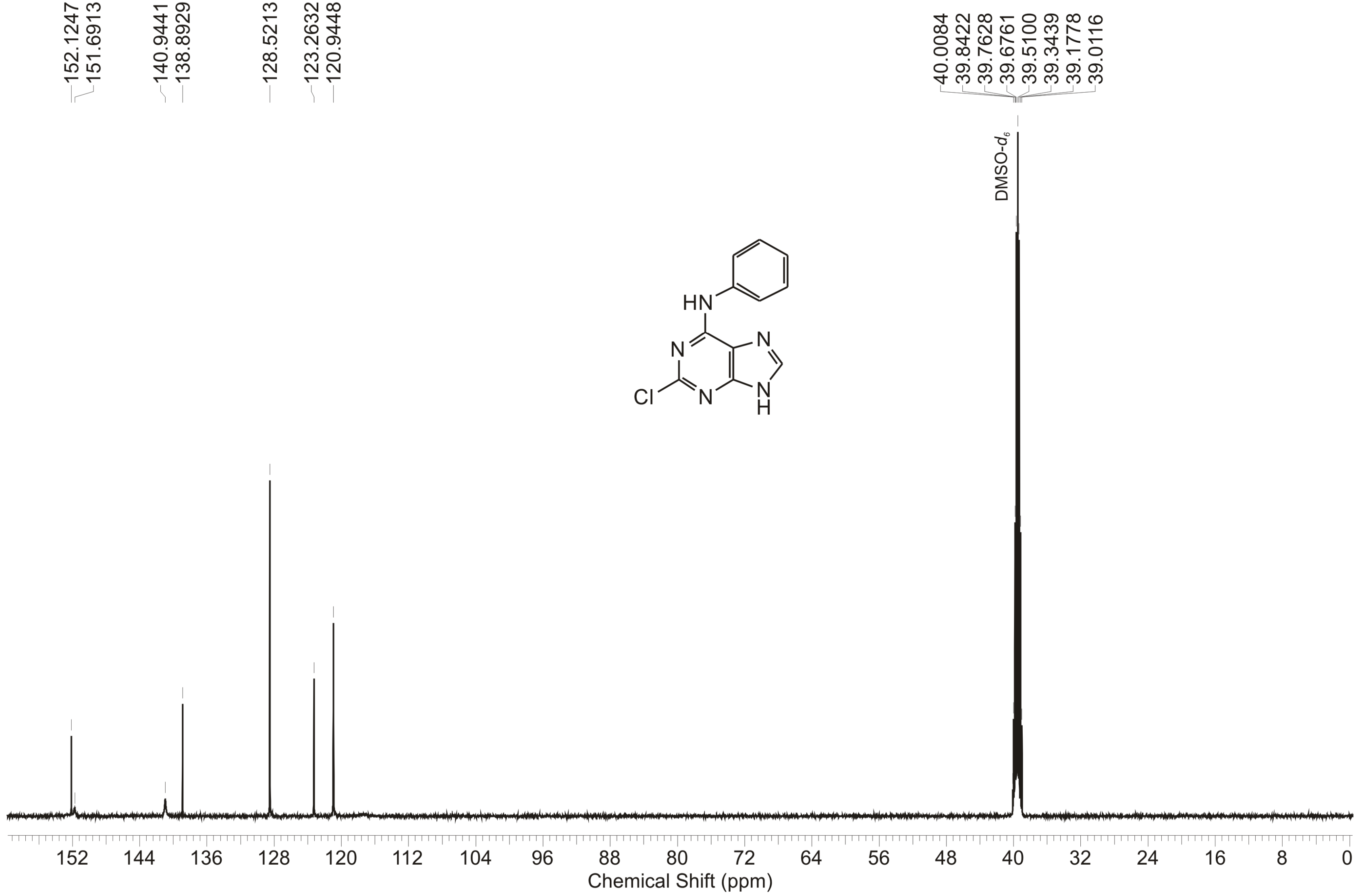

Figure S9. ^{13}C NMR spectrum (DMSO-d_6, 125 MHz, 303 K) of compound **7**.

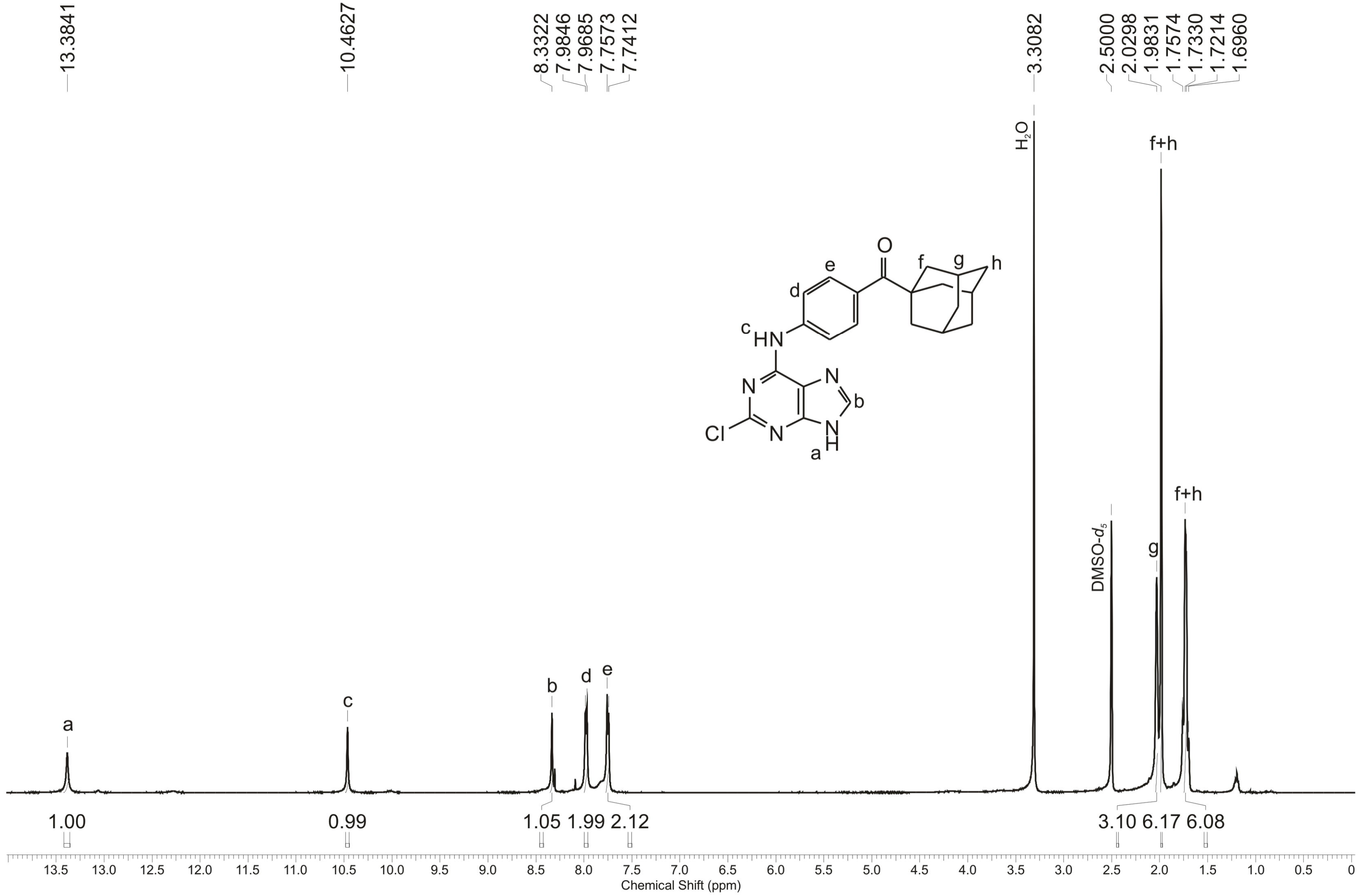

Figure S10. ^{1}H NMR spectrum (DMSO-d_6, 500 MHz, 303 K) of compound **8**.

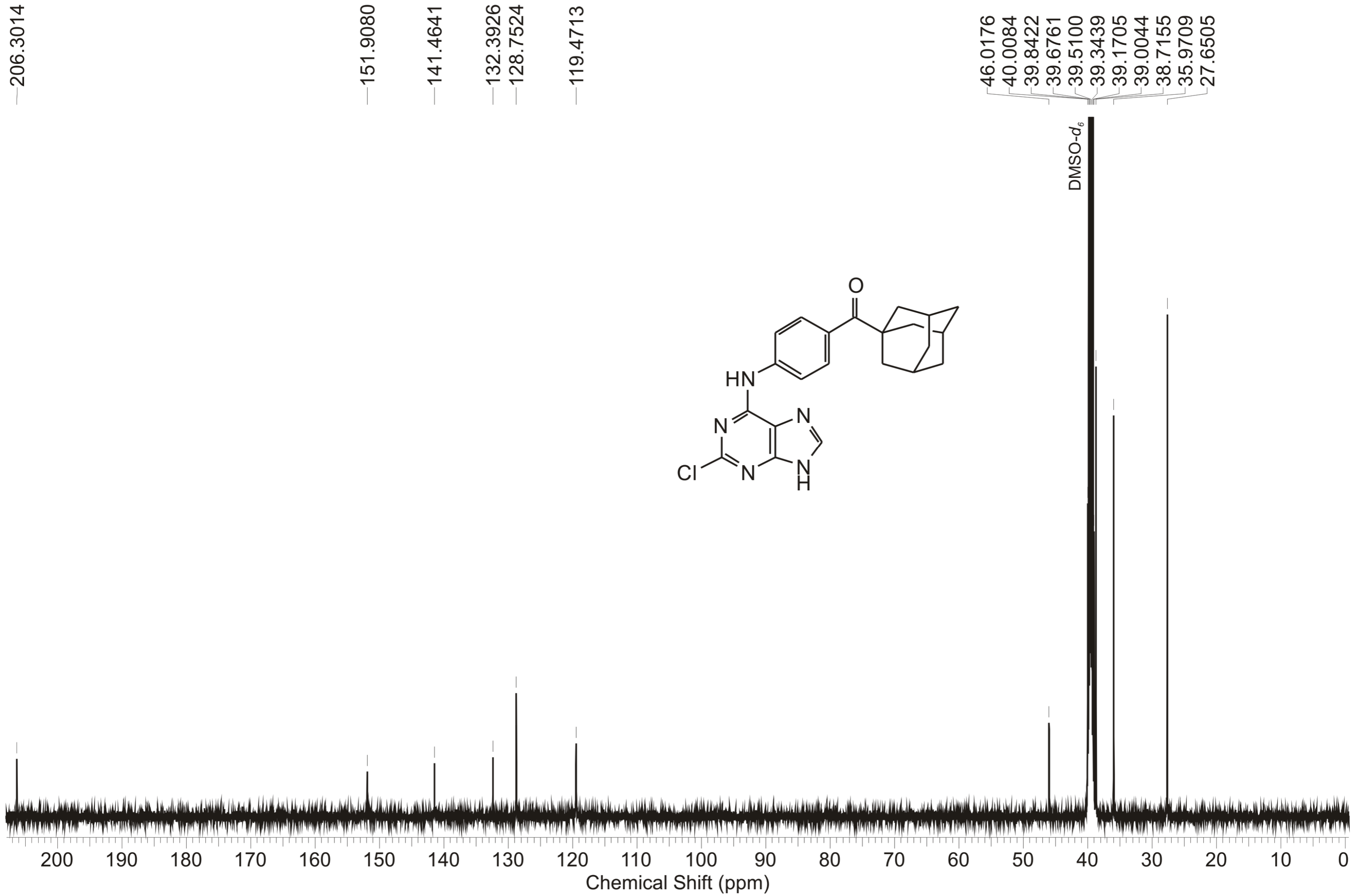

Figure S11. ^{13}C NMR spectrum (DMSO-d_6, 125 MHz, 303 K) of compound **8**.

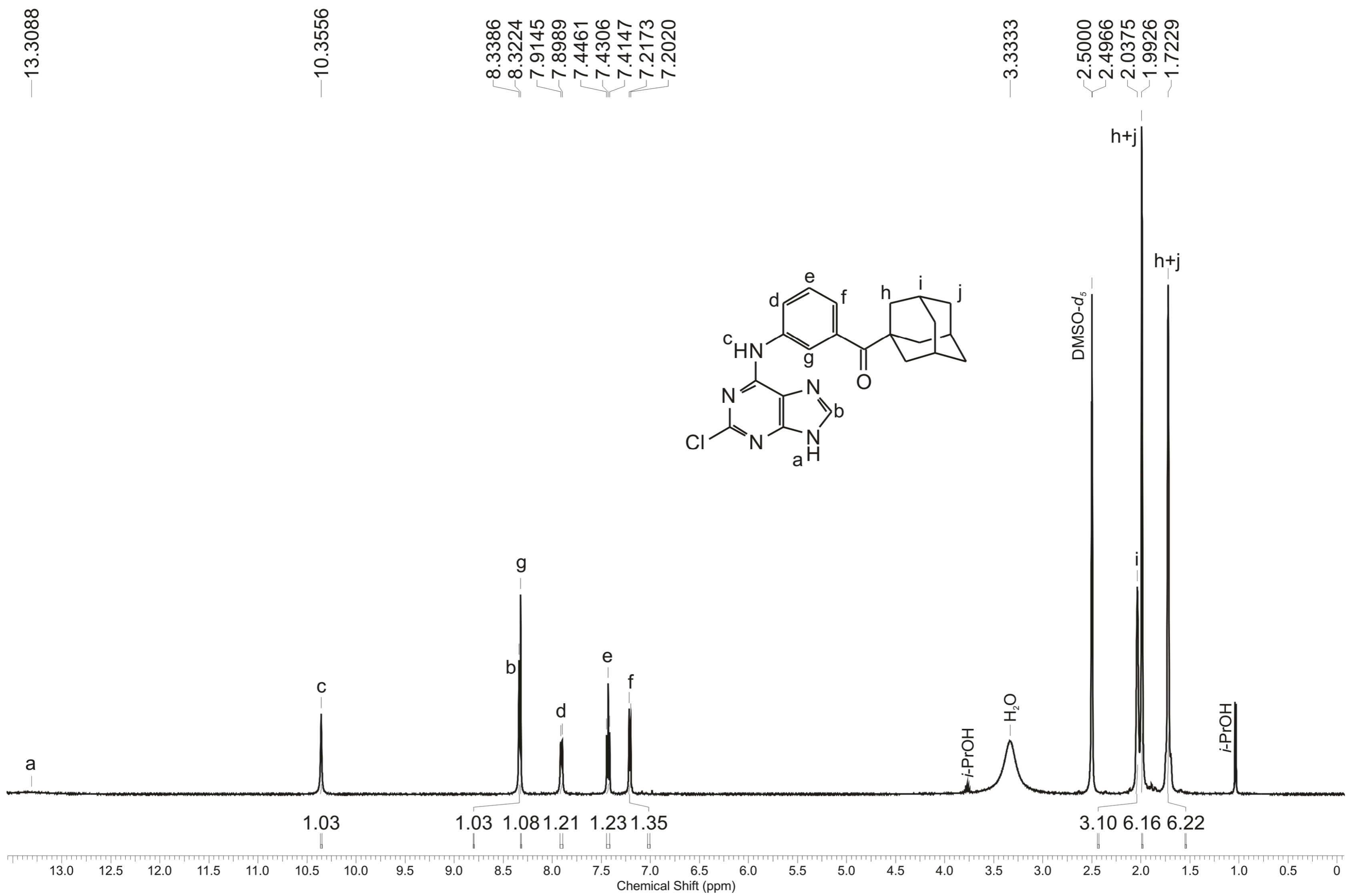

Figure S12. ^{1}H NMR spectrum (DMSO-d_6, 500 MHz, 303 K) of compound **9**.

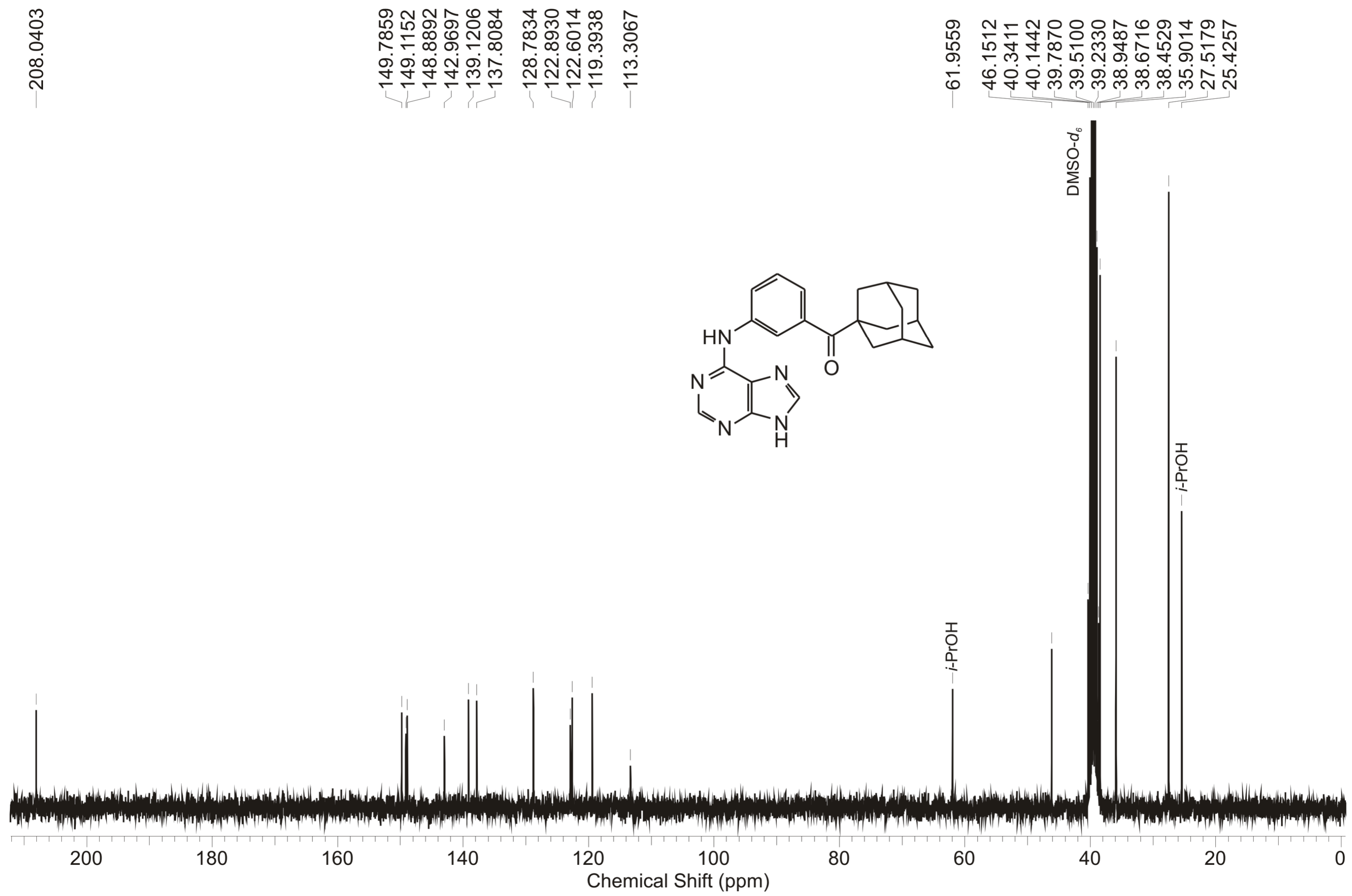

Figure S13. ^{13}C NMR spectrum (DMSO-d_6, 125 MHz, 303 K) of compound **9**.

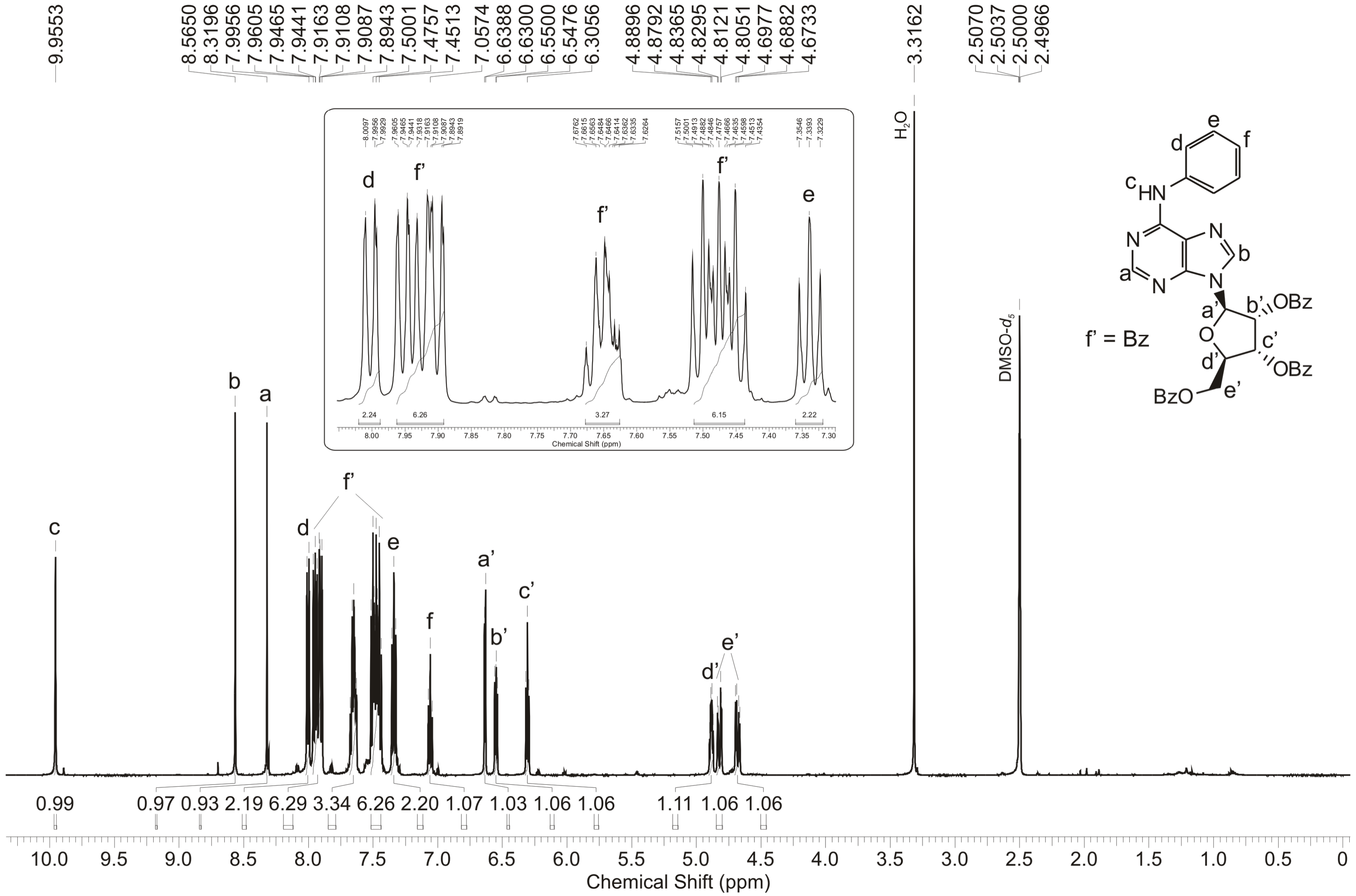

Figure S14. ^{1}H NMR spectrum (DMSO-d_6, 500 MHz, 303 K) of compound **10**.

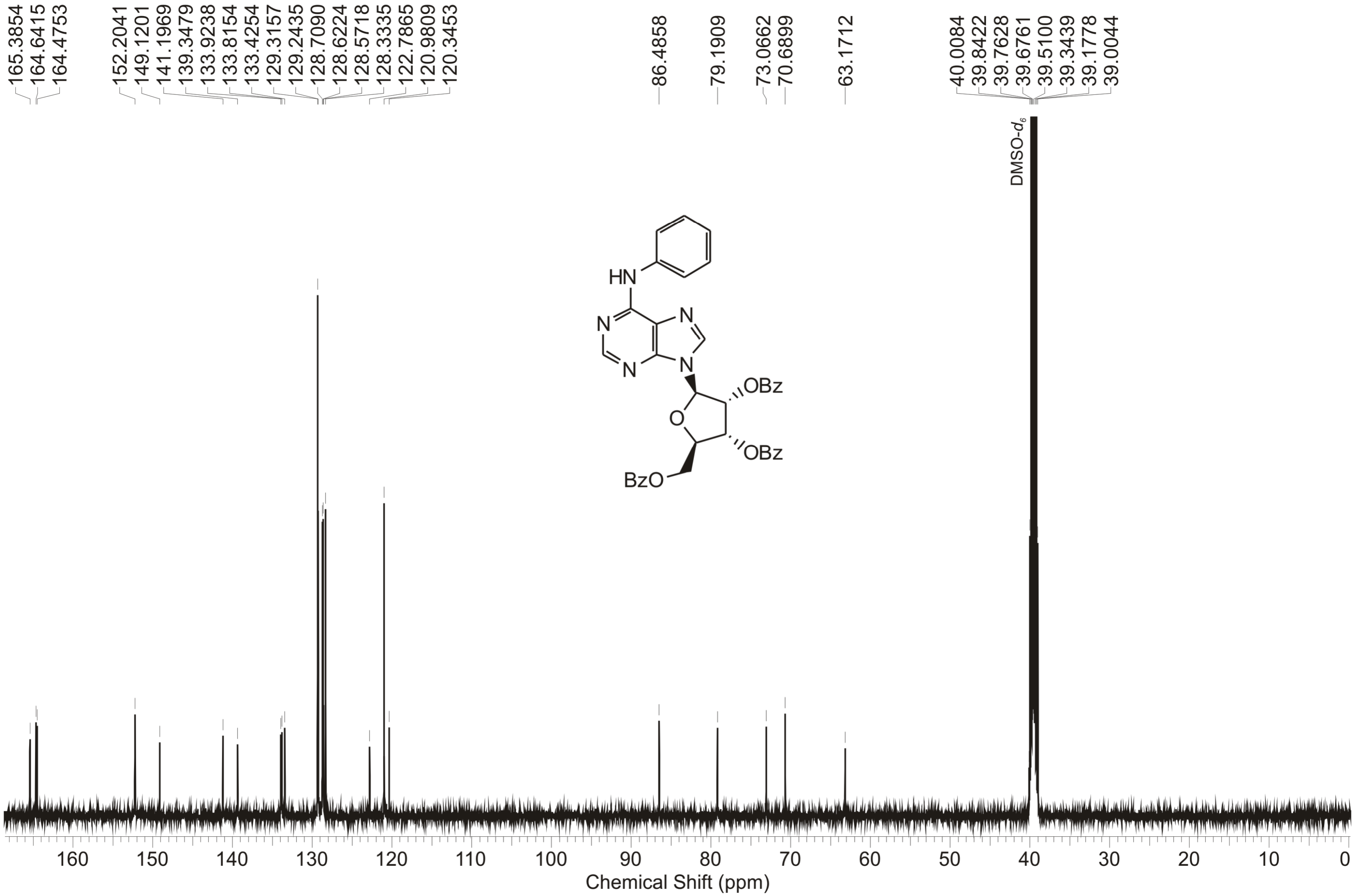

Figure S15. ^{13}C NMR spectrum (DMSO-d_6, 125 MHz, 303 K) of compound **10**.

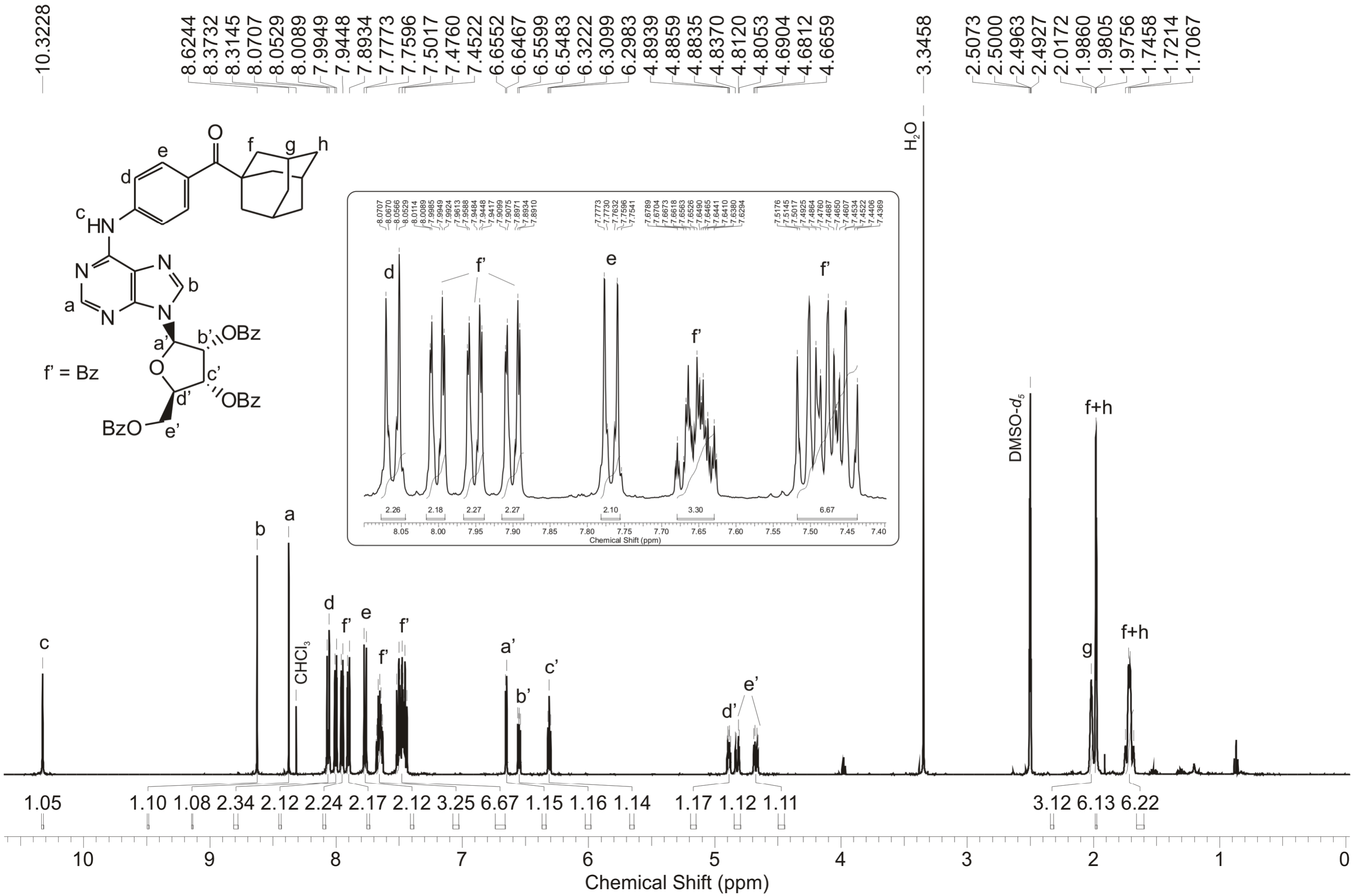

Figure S16. ^{1}H NMR spectrum (DMSO-d_6, 500 MHz, 303 K) of compound **11**.

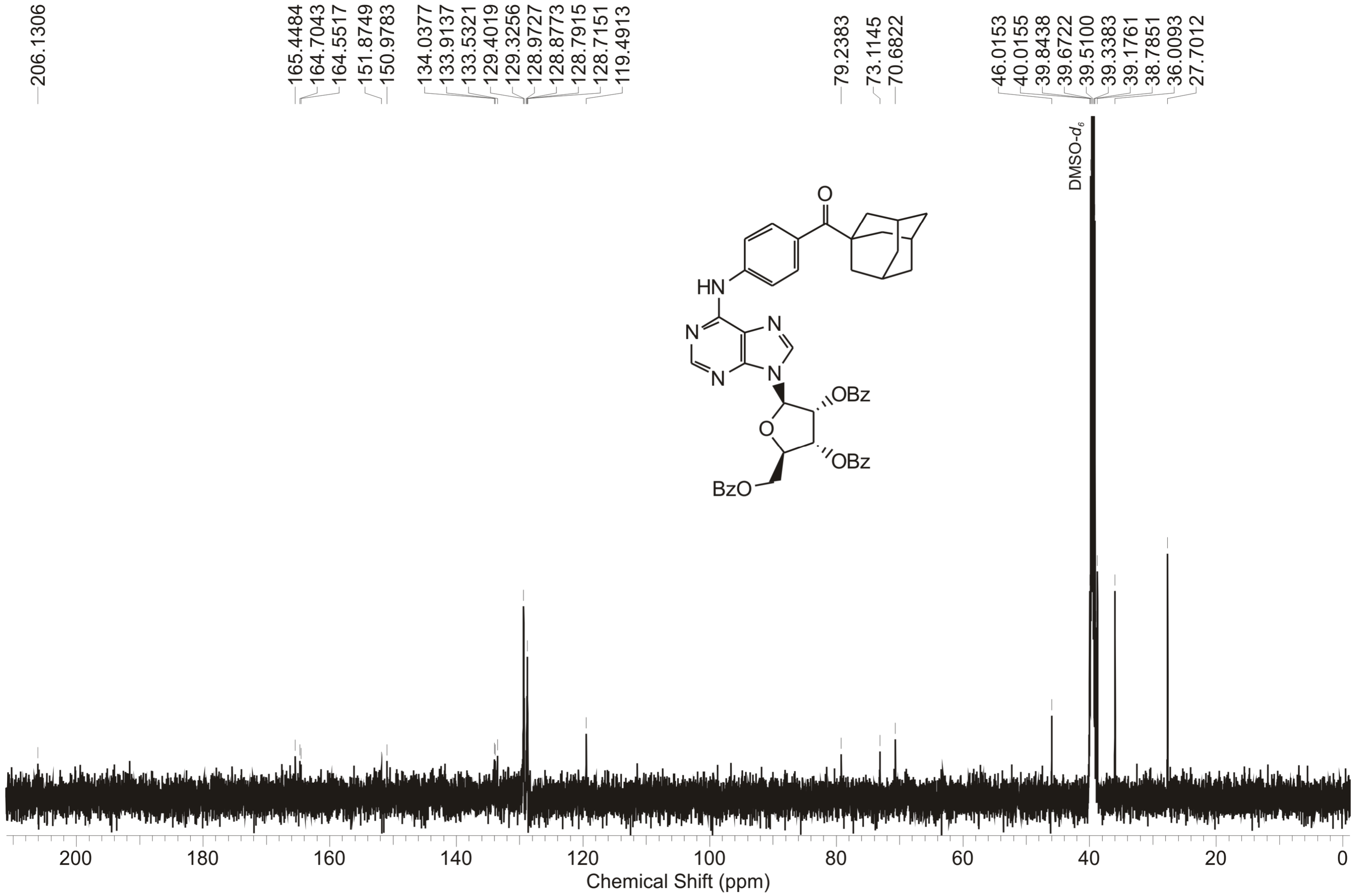

Figure S17. ^{13}C NMR spectrum (DMSO-d_6, 125 MHz, 303 K) of compound **11**.

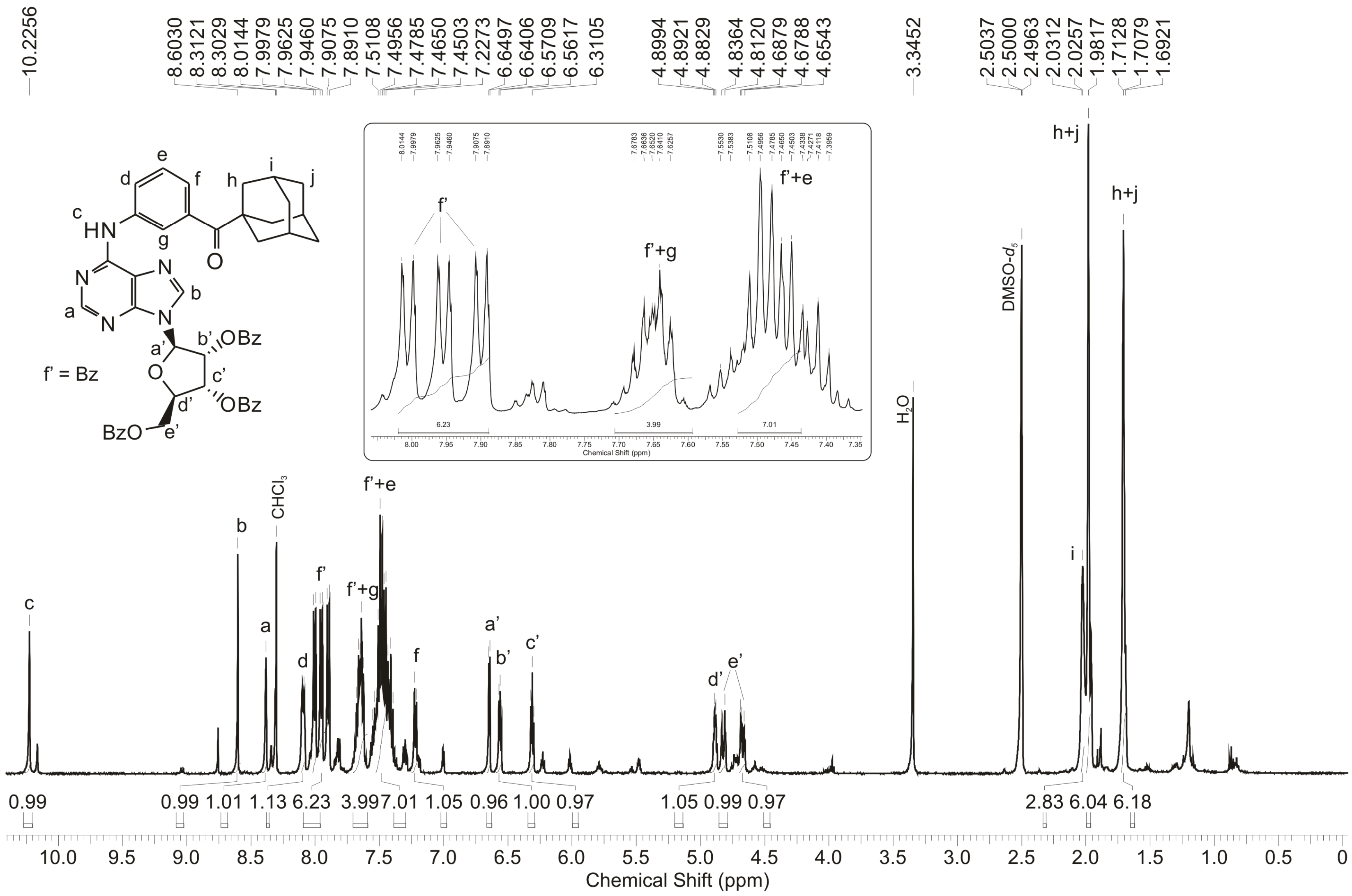

Figure S18. ^{1}H NMR spectrum (DMSO-d_6, 500 MHz, 303 K) of compound **12**.

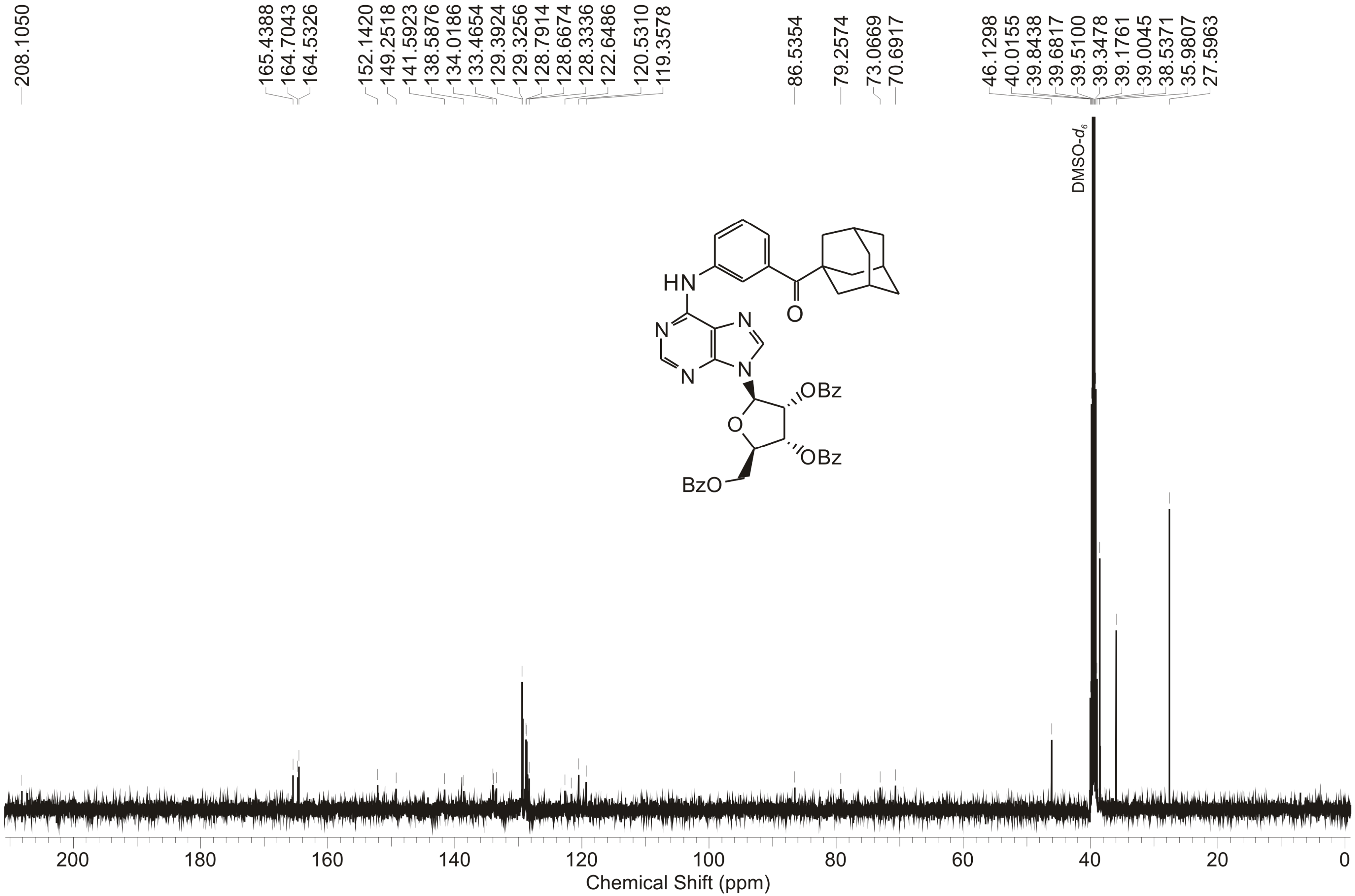

Figure S19. ^{13}C NMR spectrum (DMSO-d_6, 125 MHz, 303 K) of compound **12**.

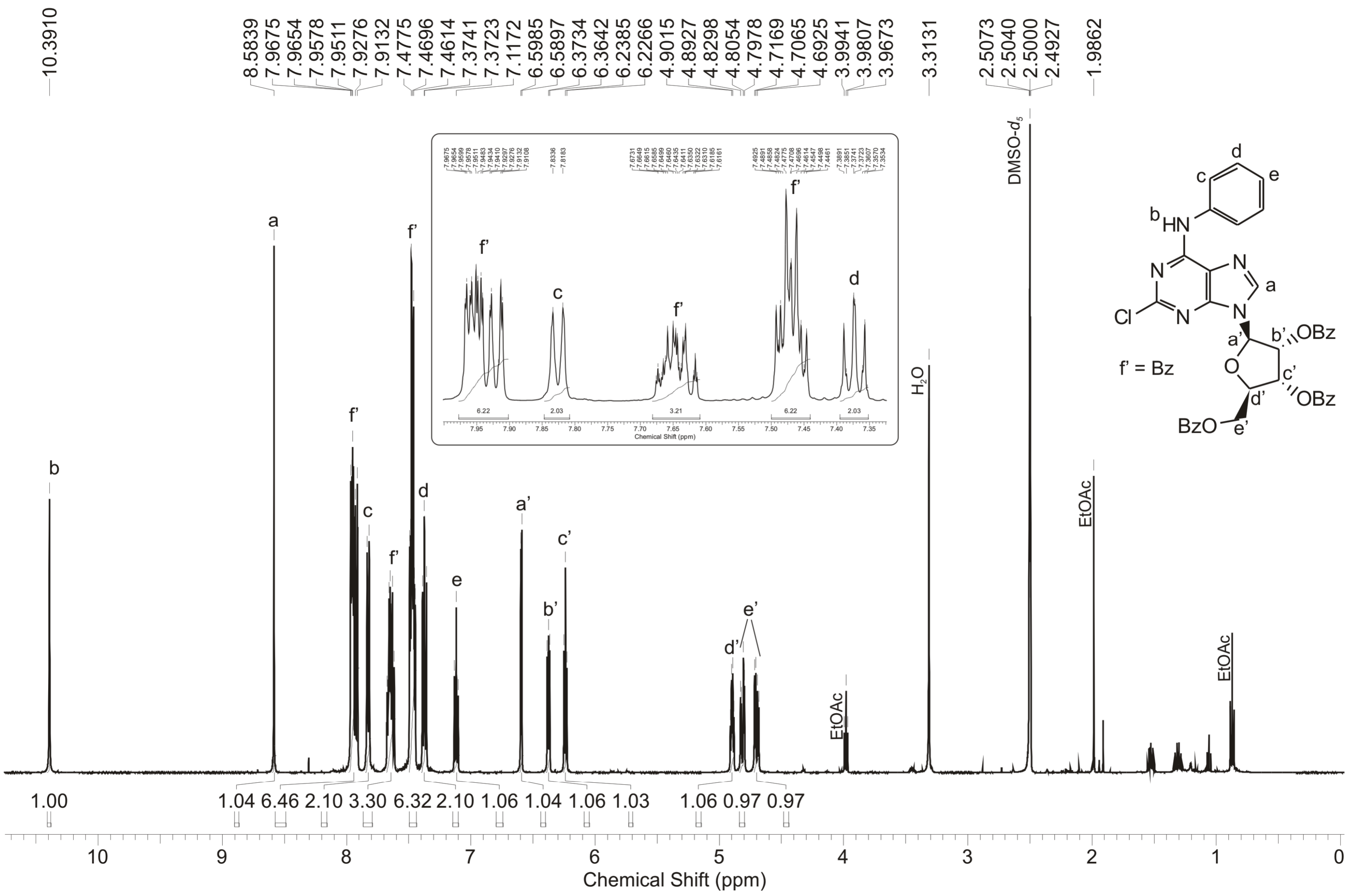

Figure S20. ^{1}H NMR spectrum (DMSO-d_6, 500 MHz, 303 K) of compound **13**.

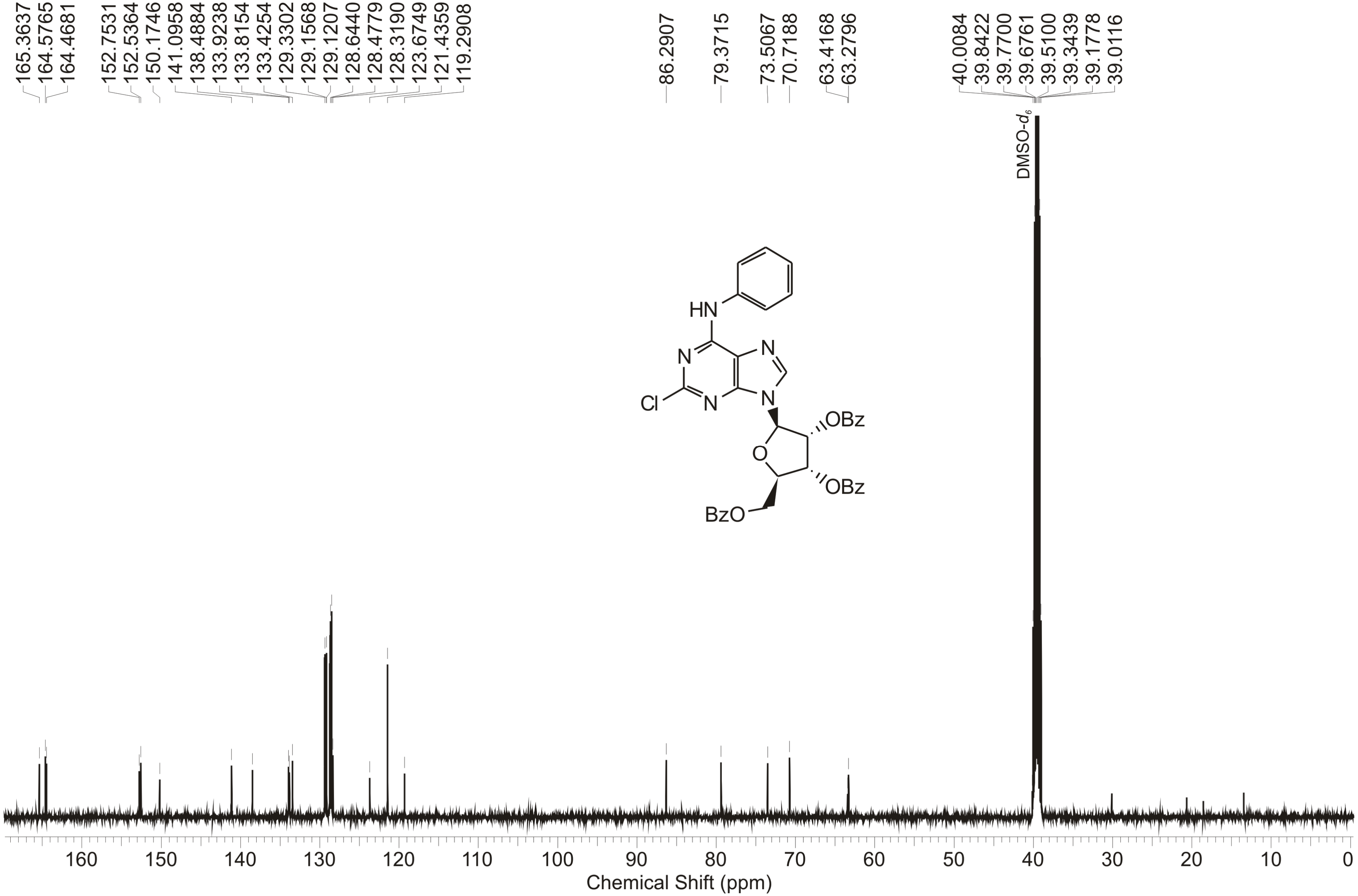

Figure S21. ^{13}C NMR spectrum (DMSO-d_6, 125 MHz, 303 K) of compound **13**.

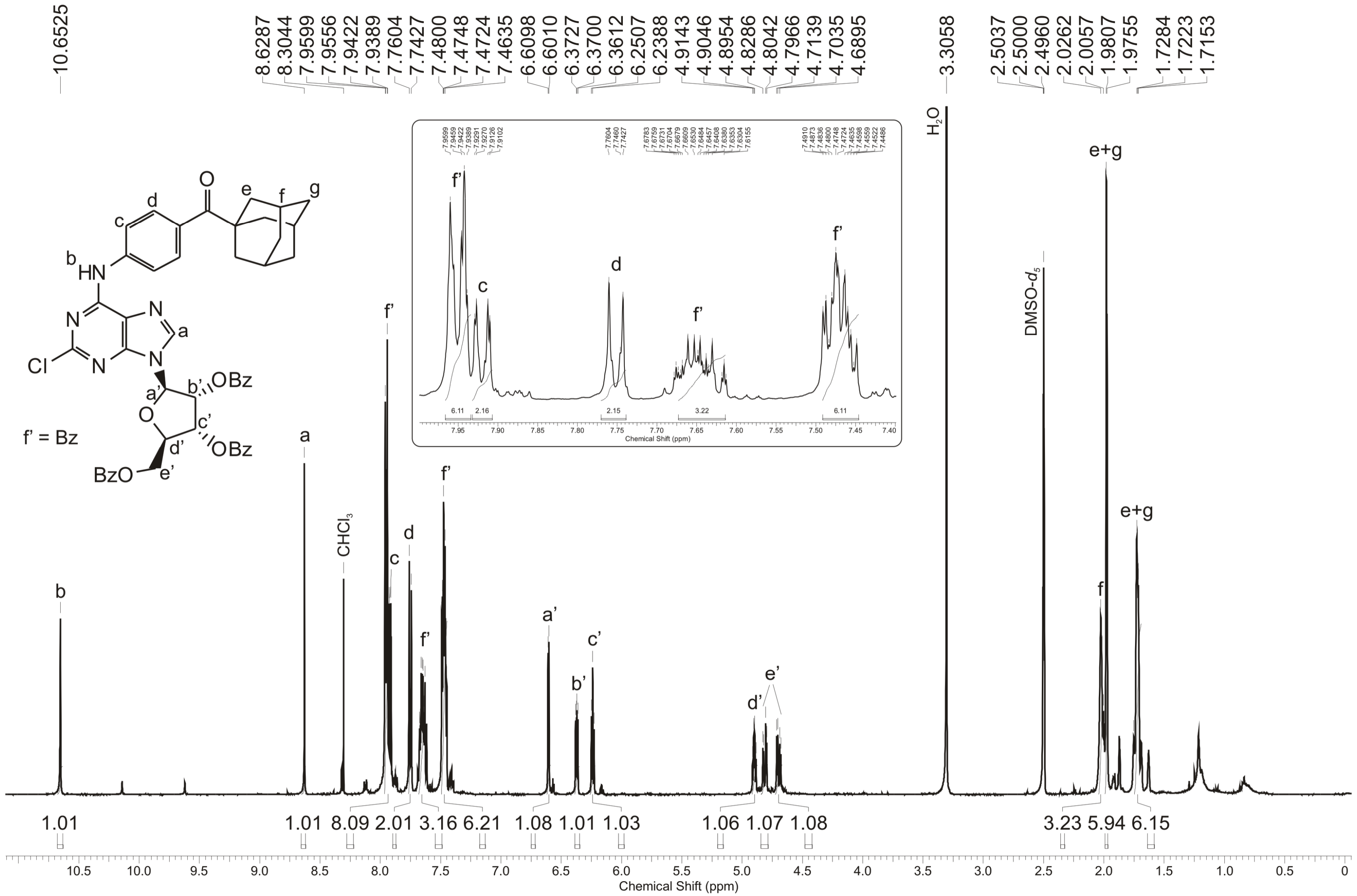

Figure S22. ^{1}H NMR spectrum (DMSO-d_6, 500 MHz, 303 K) of compound **14**.

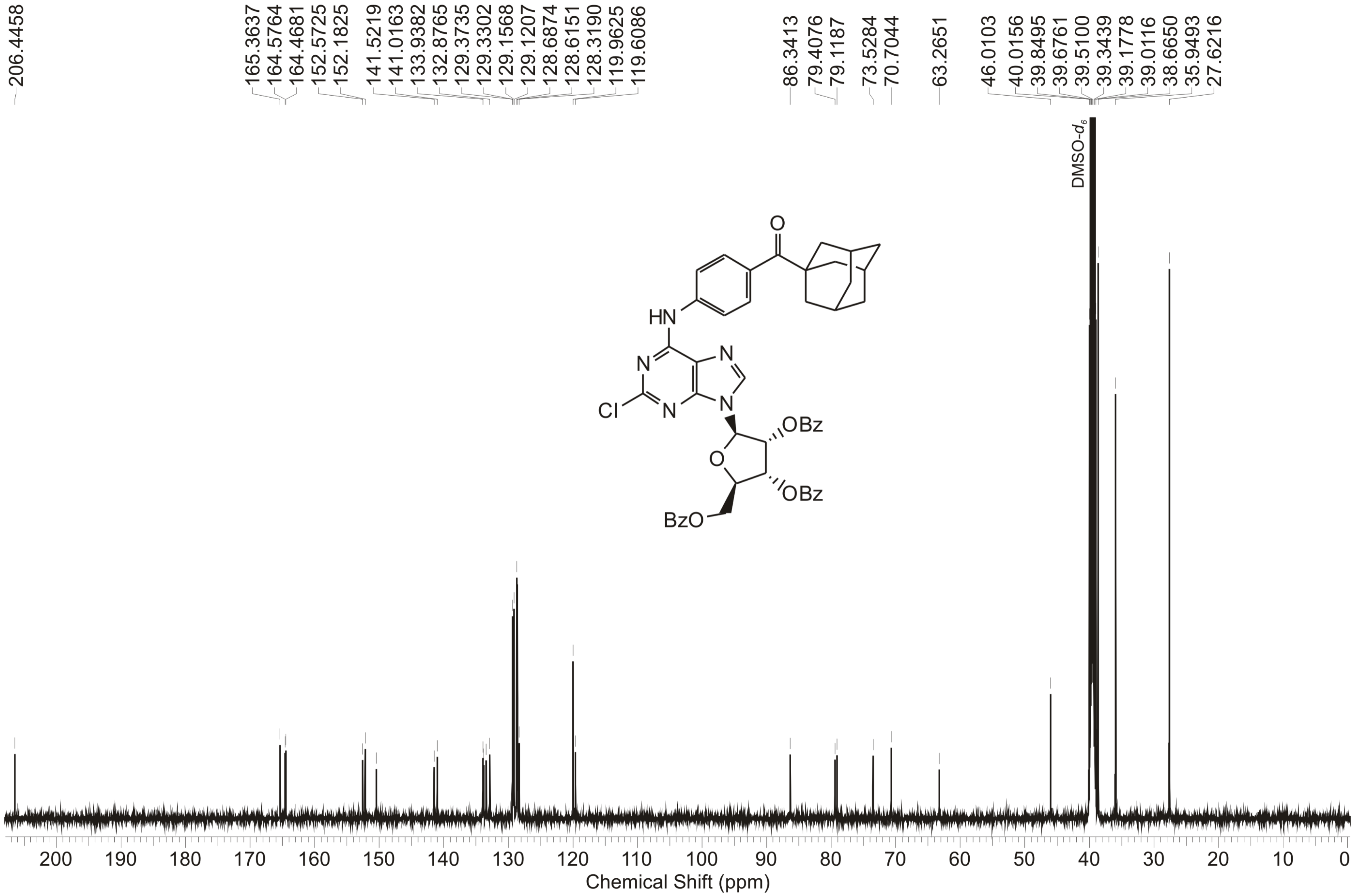

Figure S23. ^{13}C NMR spectrum (DMSO-d_6, 125 MHz, 303 K) of compound **14**.

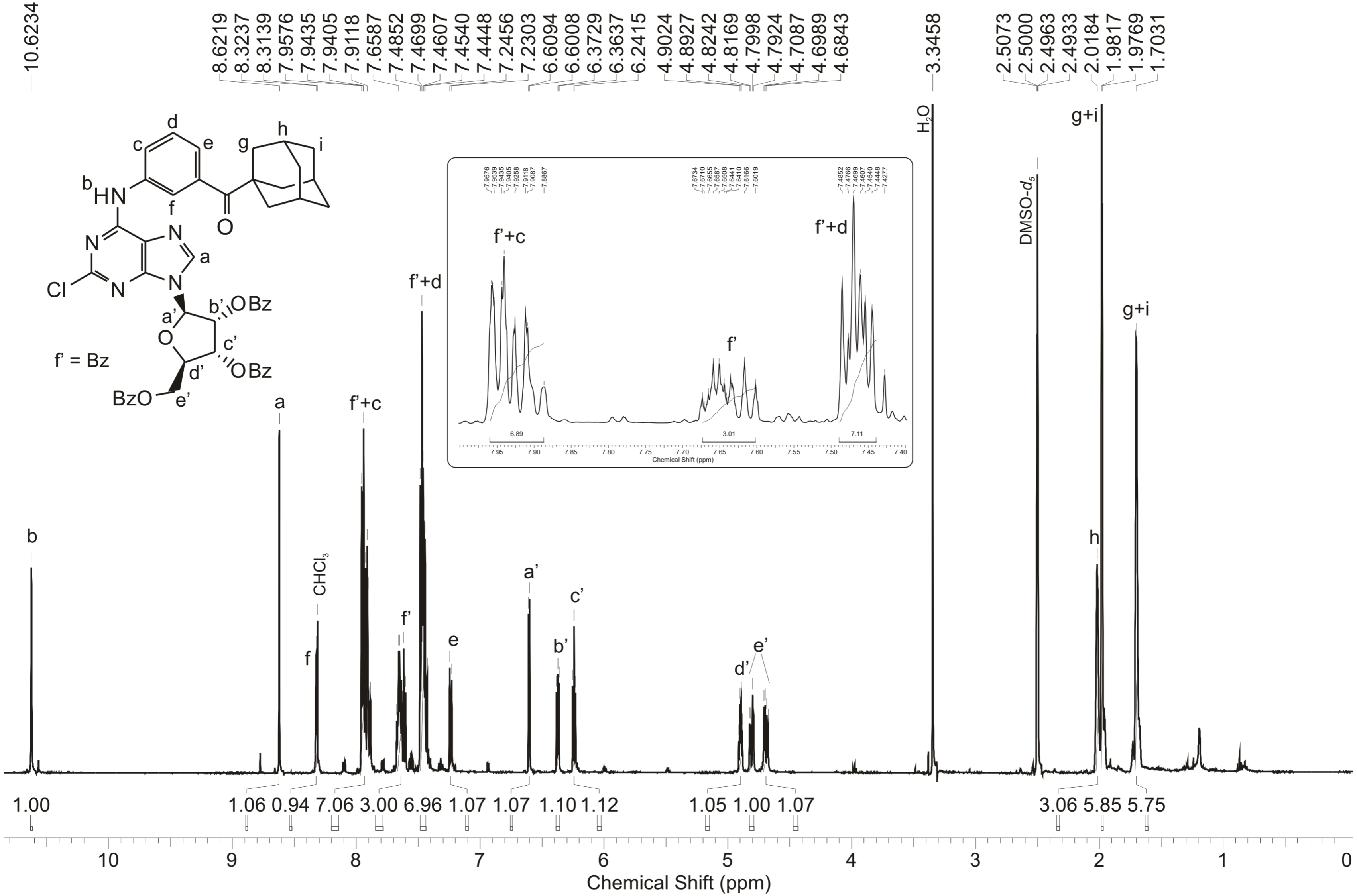

Figure S24. ^{1}H NMR spectrum (DMSO-d_6, 500 MHz, 303 K) of compound **15**.

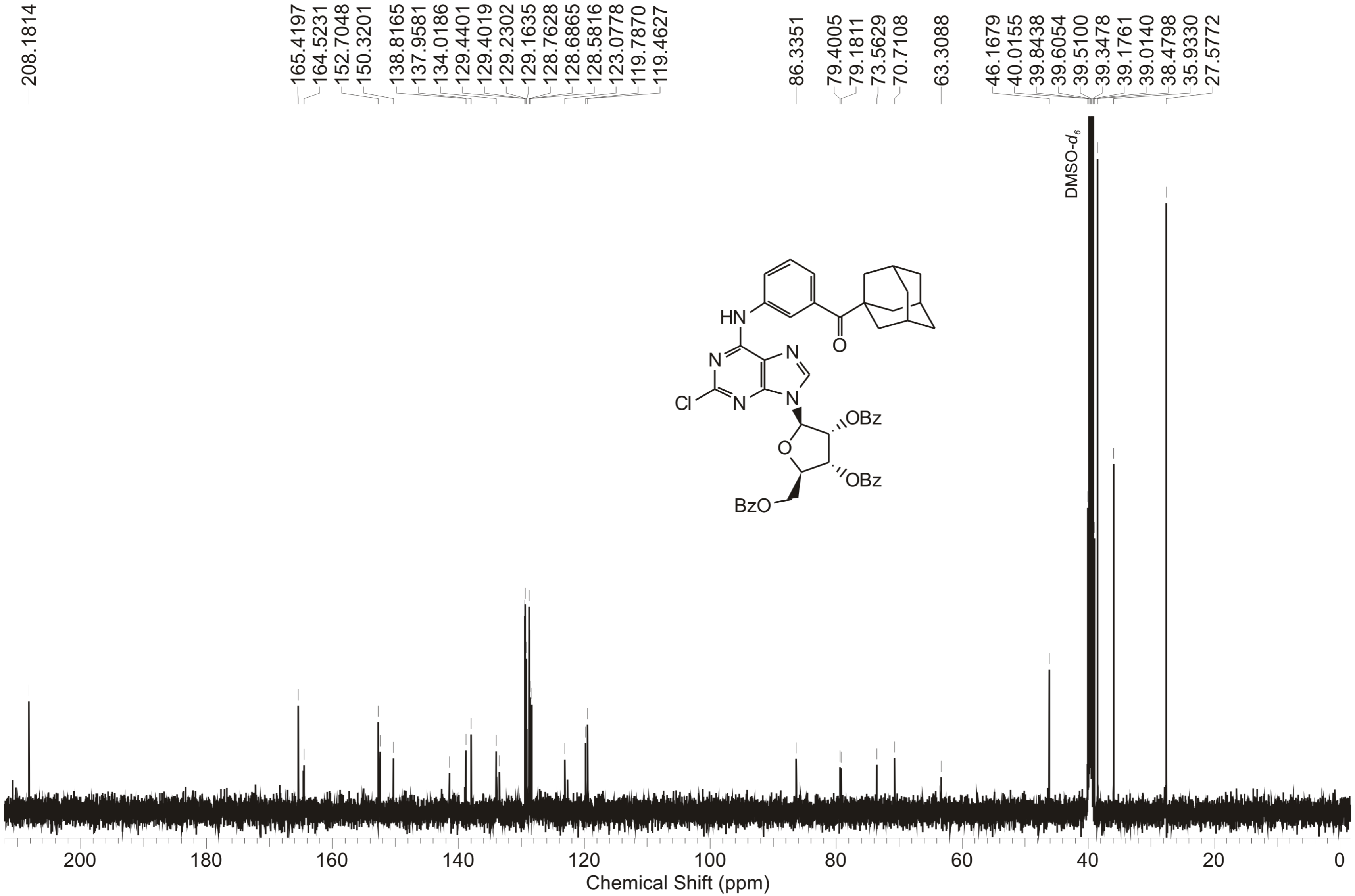

Figure S25. ^{13}C NMR spectrum (DMSO-d_6, 125 MHz, 303 K) of compound **15**.

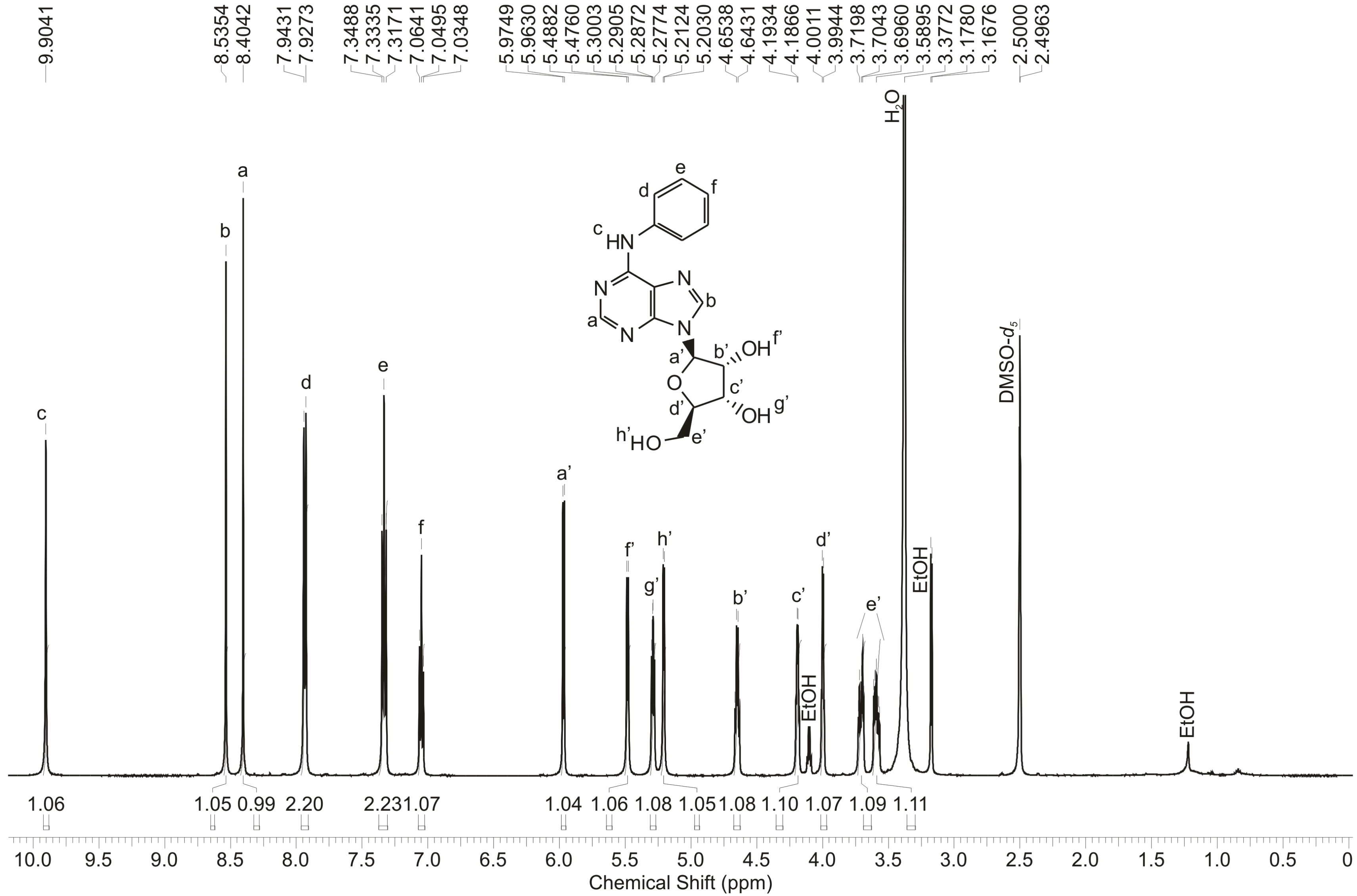

Figure S26. ^{1}H NMR spectrum (DMSO-d_6, 500 MHz, 303 K) of compound **16**.

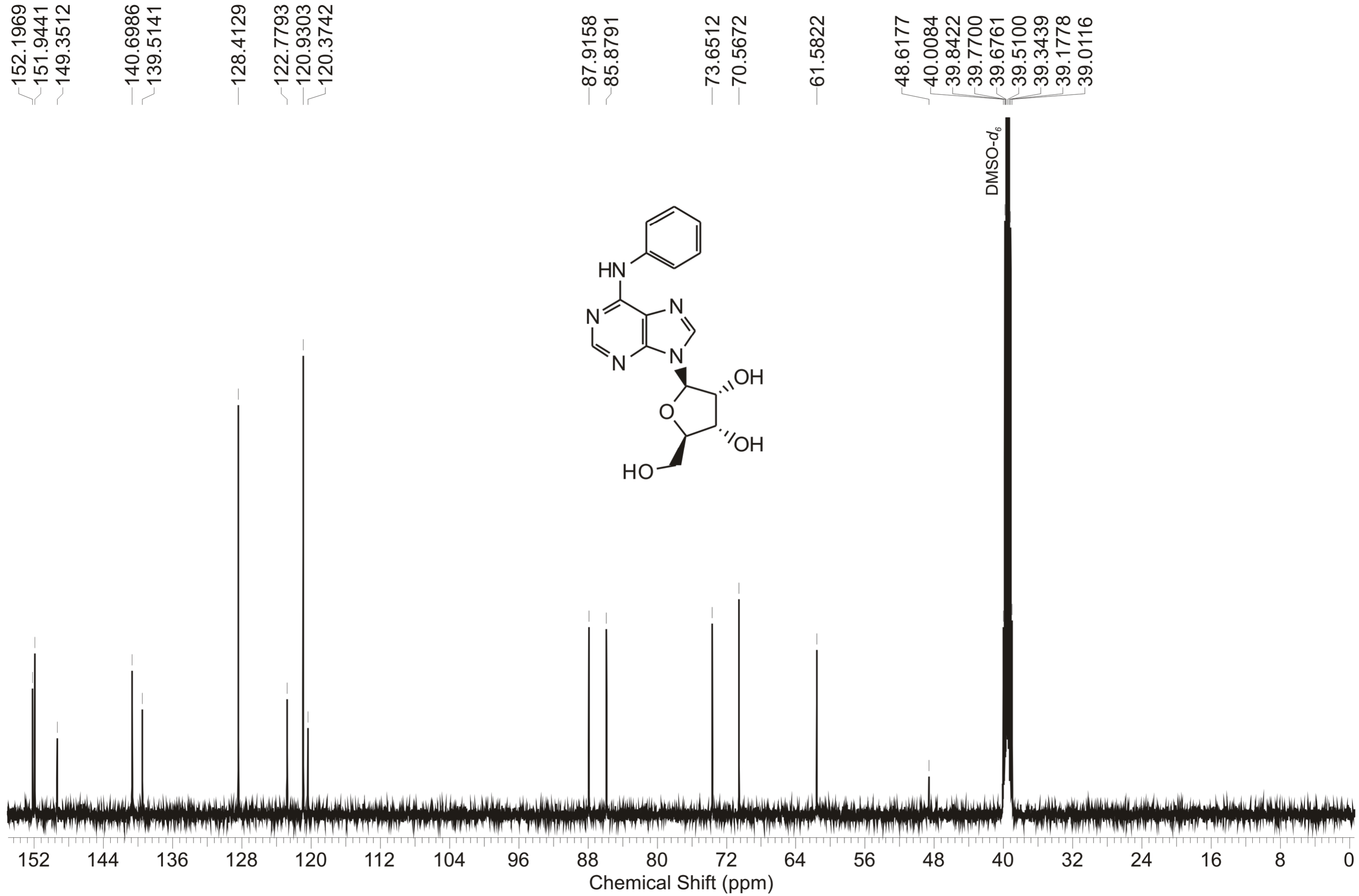

Figure S27. ^{13}C NMR spectrum (DMSO-d_6, 125 MHz, 303 K) of compound **16**.

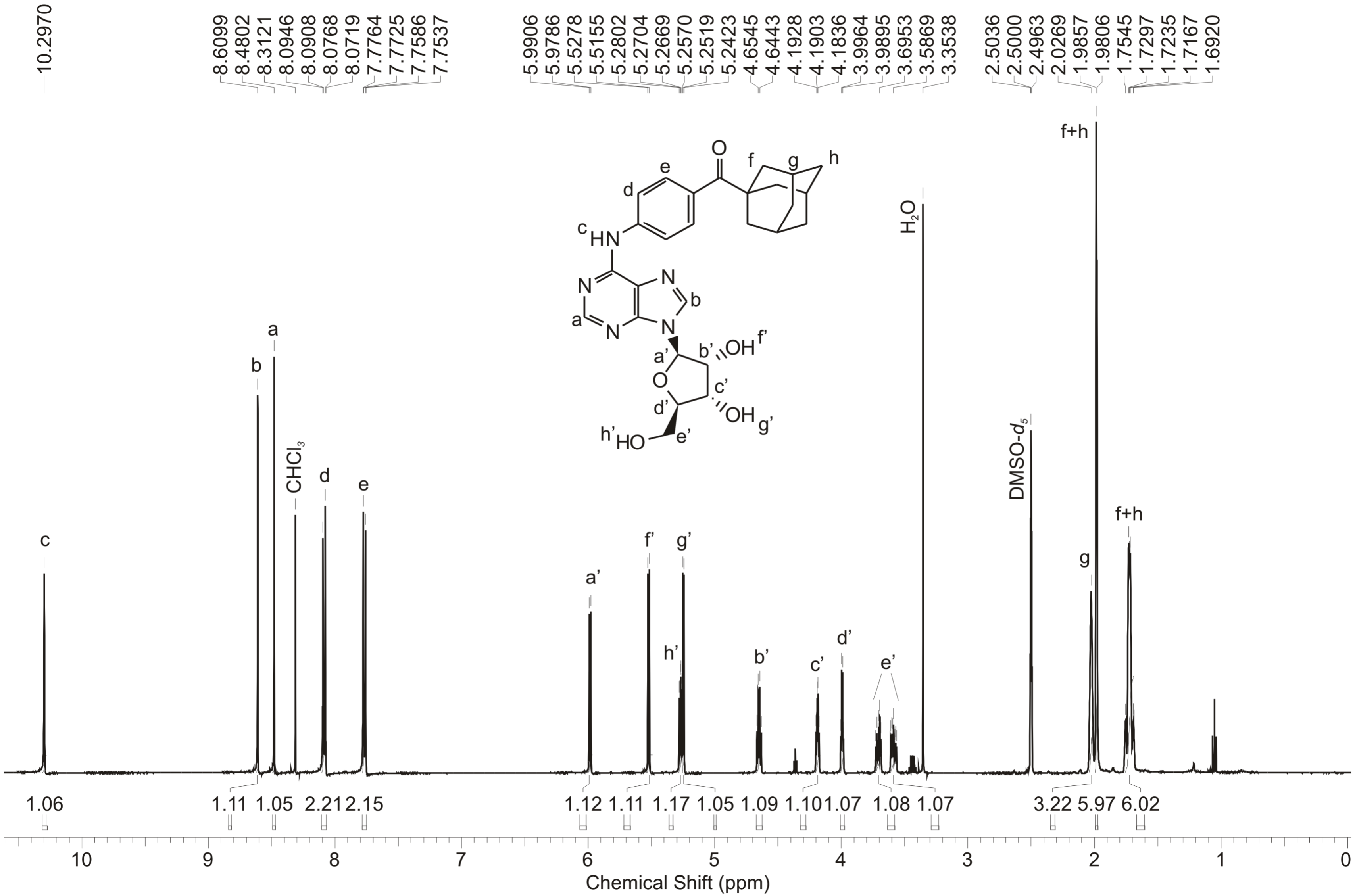

Figure S28. ^{1}H NMR spectrum (DMSO-d_6, 500 MHz, 303 K) of compound **17**.

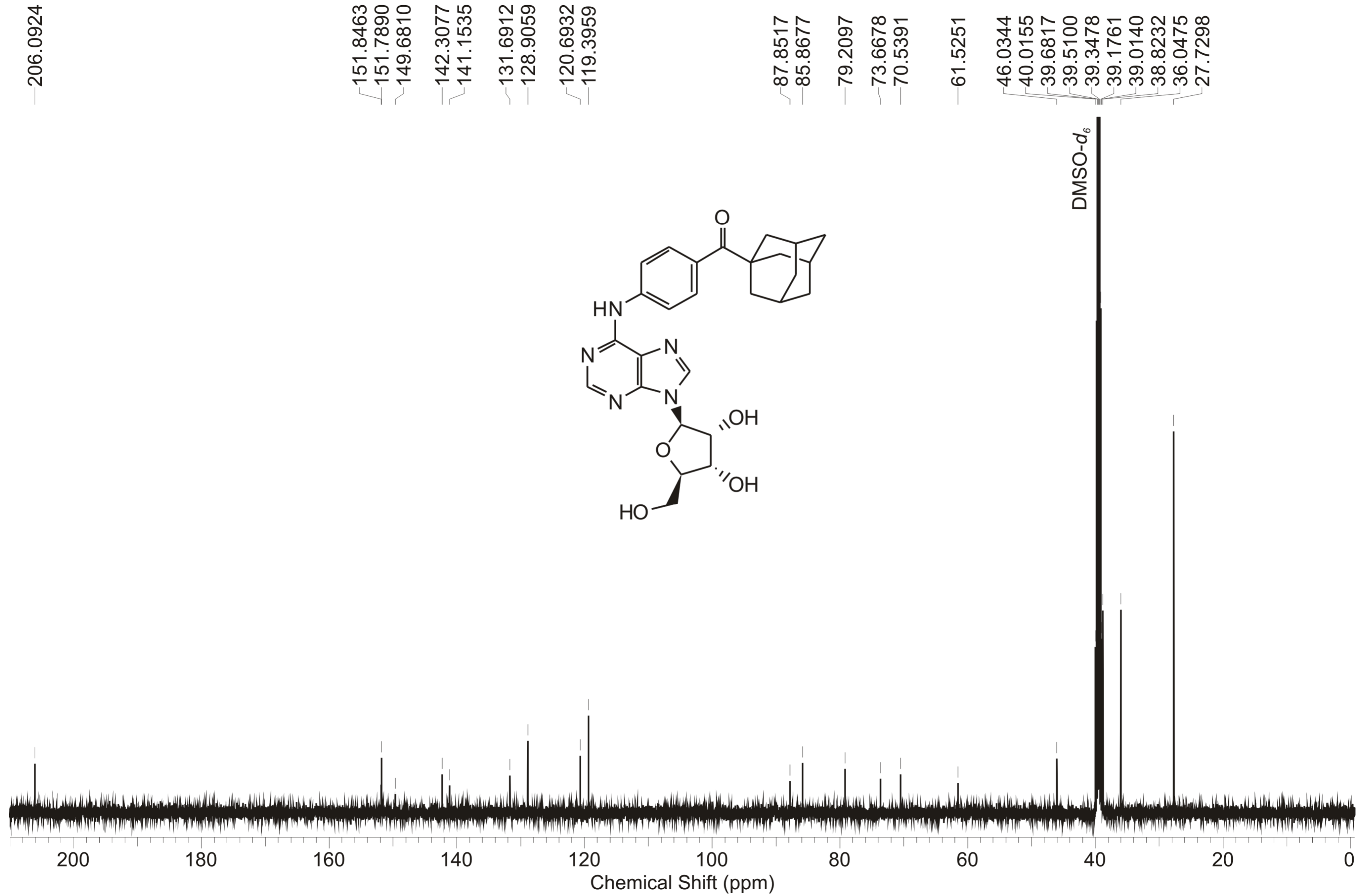

Figure S29. ^{13}C NMR spectrum (DMSO-d_6, 125 MHz, 303 K) of compound **17**.

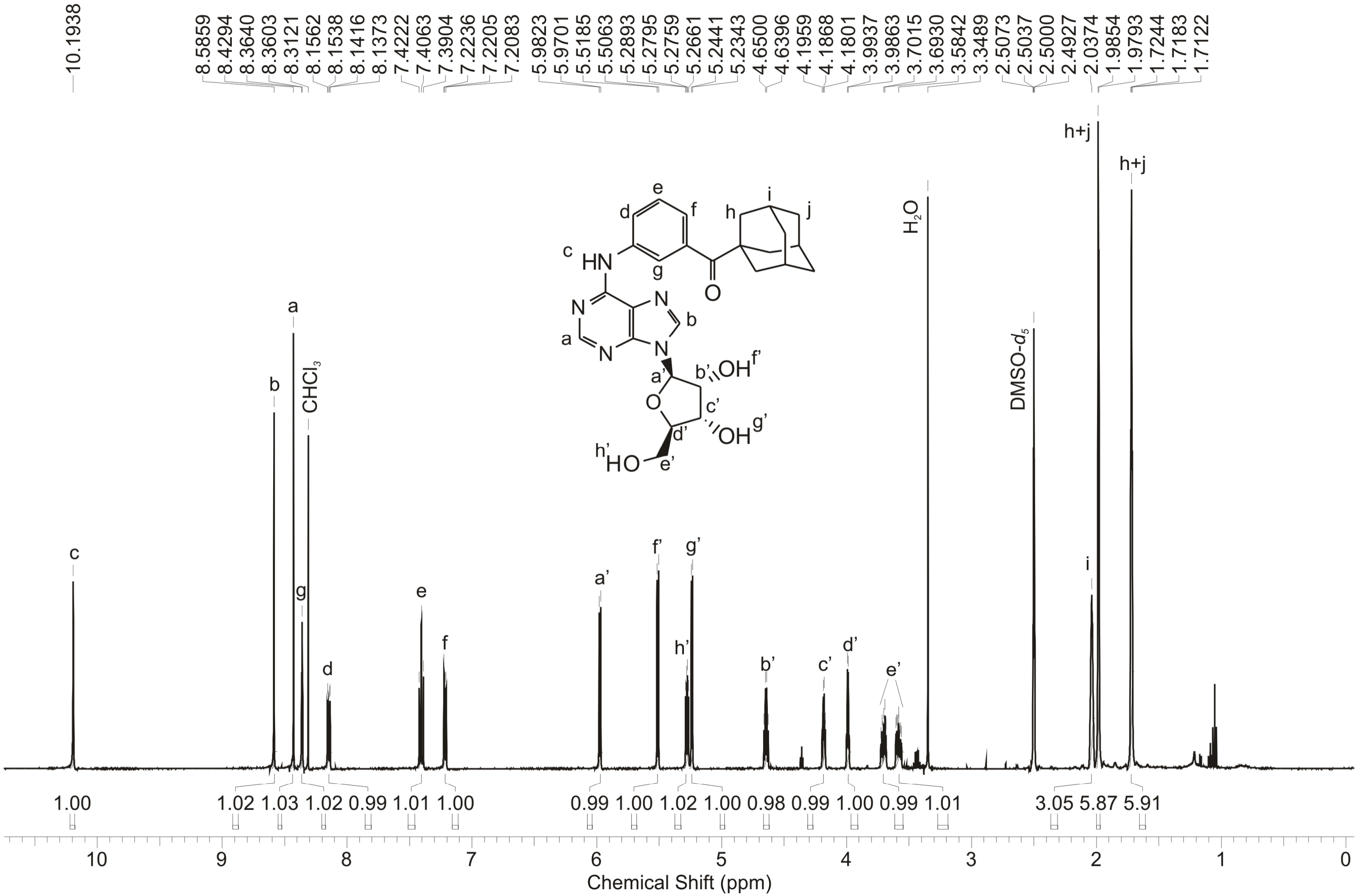

Figure S30. ^{1}H NMR spectrum (DMSO-d_6, 500 MHz, 303 K) of compound **18**.

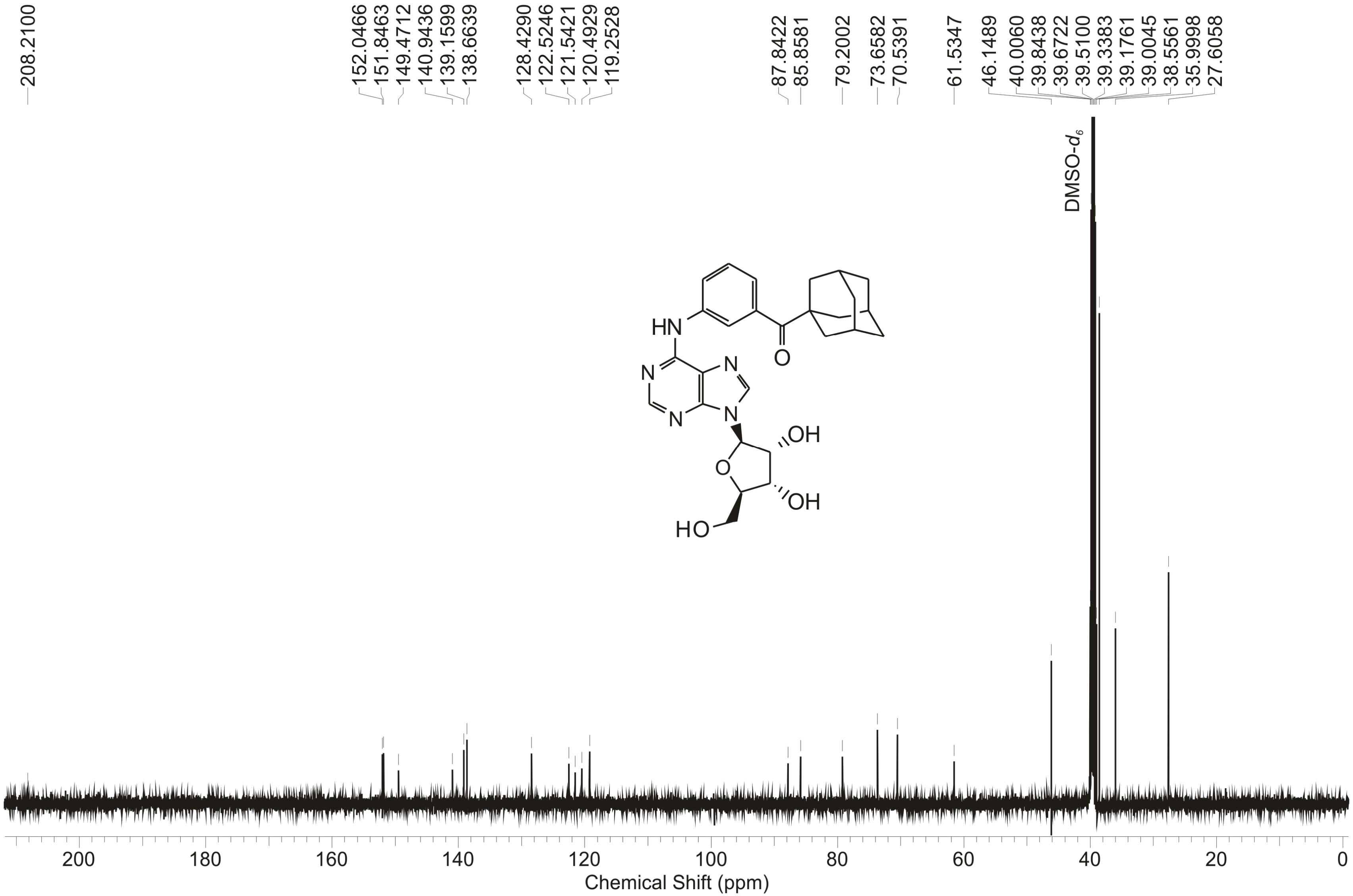

Figure S31. ^{13}C NMR spectrum (DMSO-d_6, 125 MHz, 303 K) of compound **18**.

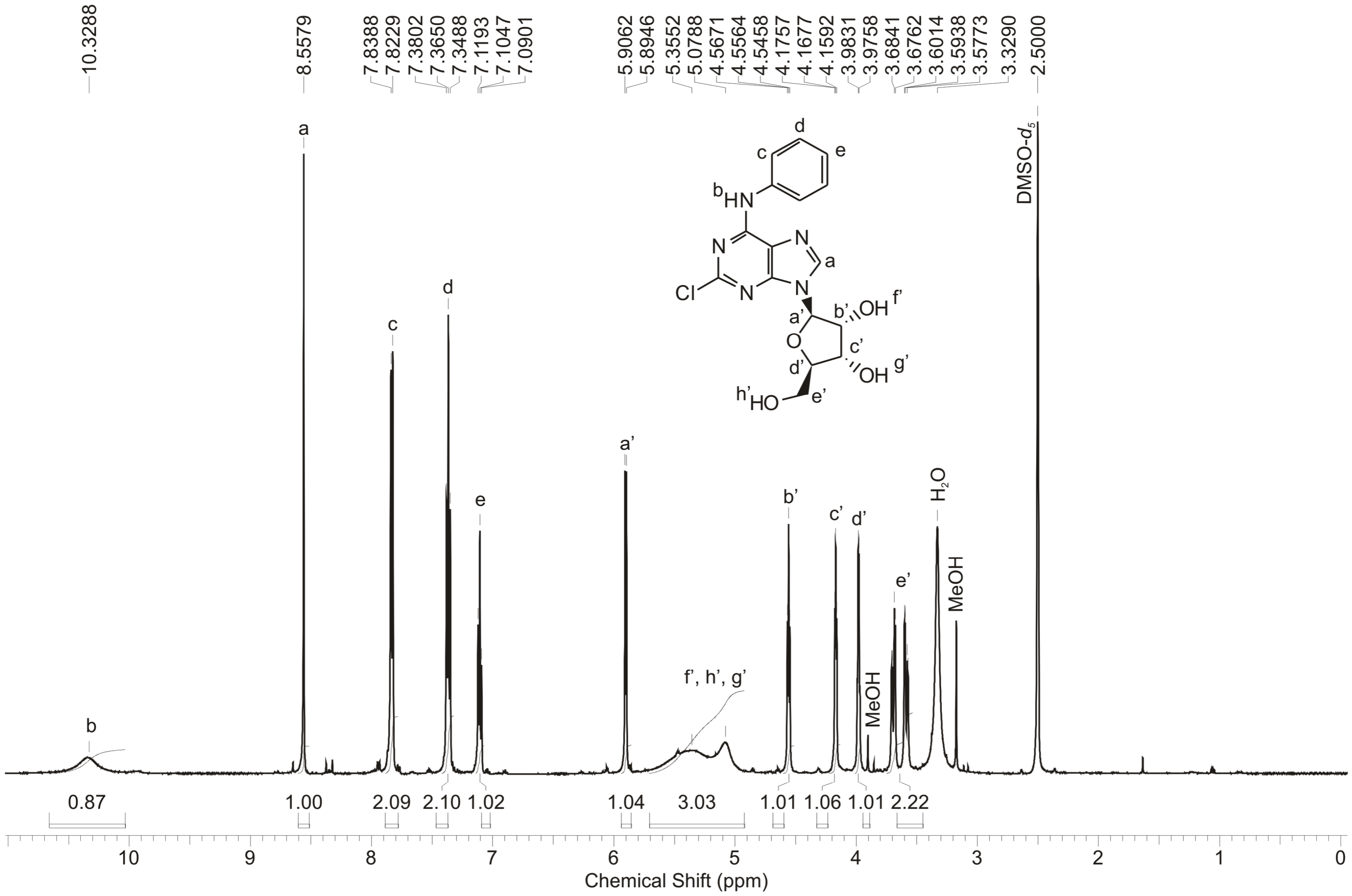

Figure S32. ^{1}H NMR spectrum (DMSO-d_6, 500 MHz, 303 K) of compound **19**.

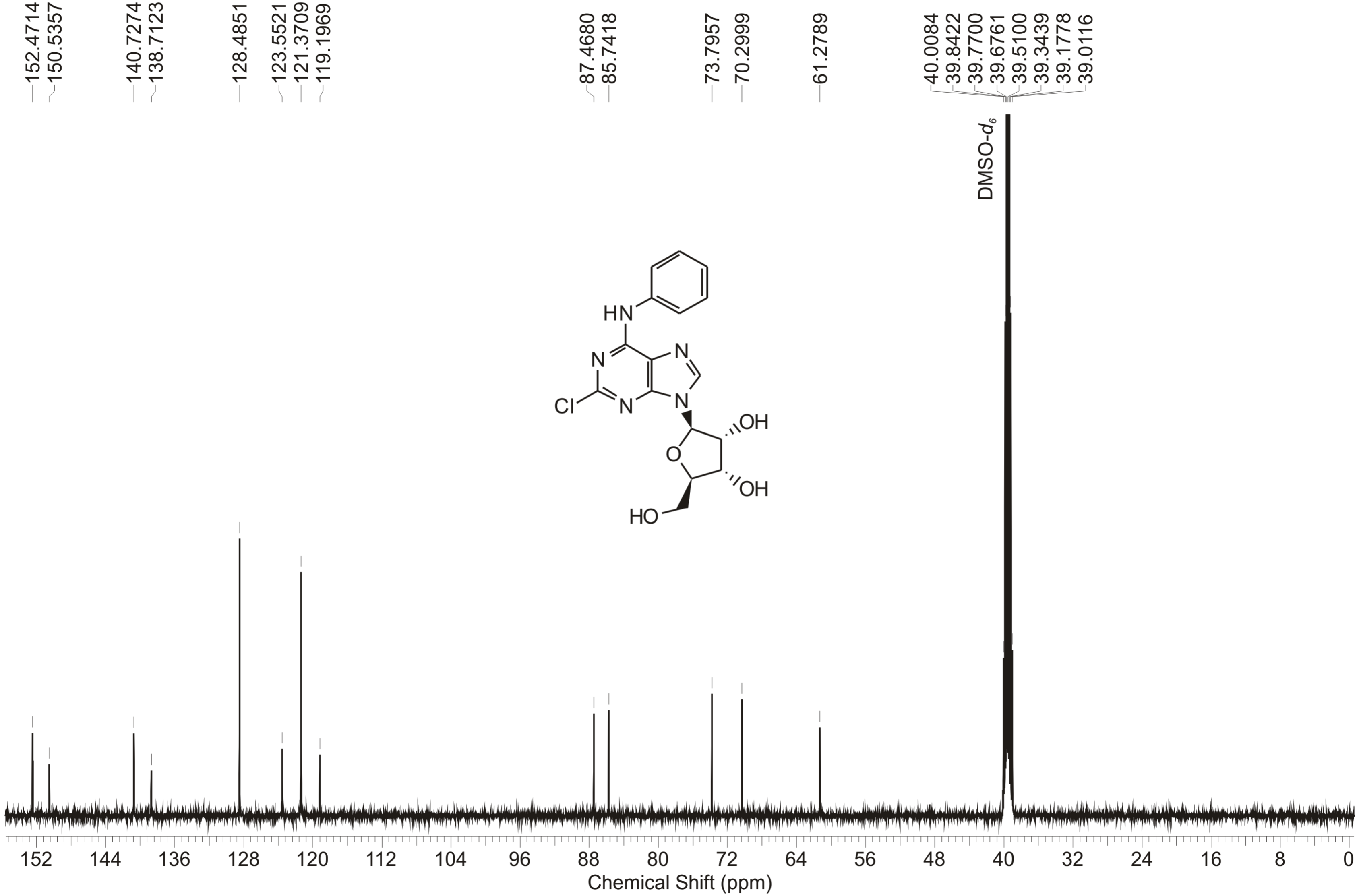

Figure S33. ^{13}C NMR spectrum (DMSO-d_6, 125 MHz, 303 K) of compound **19**.

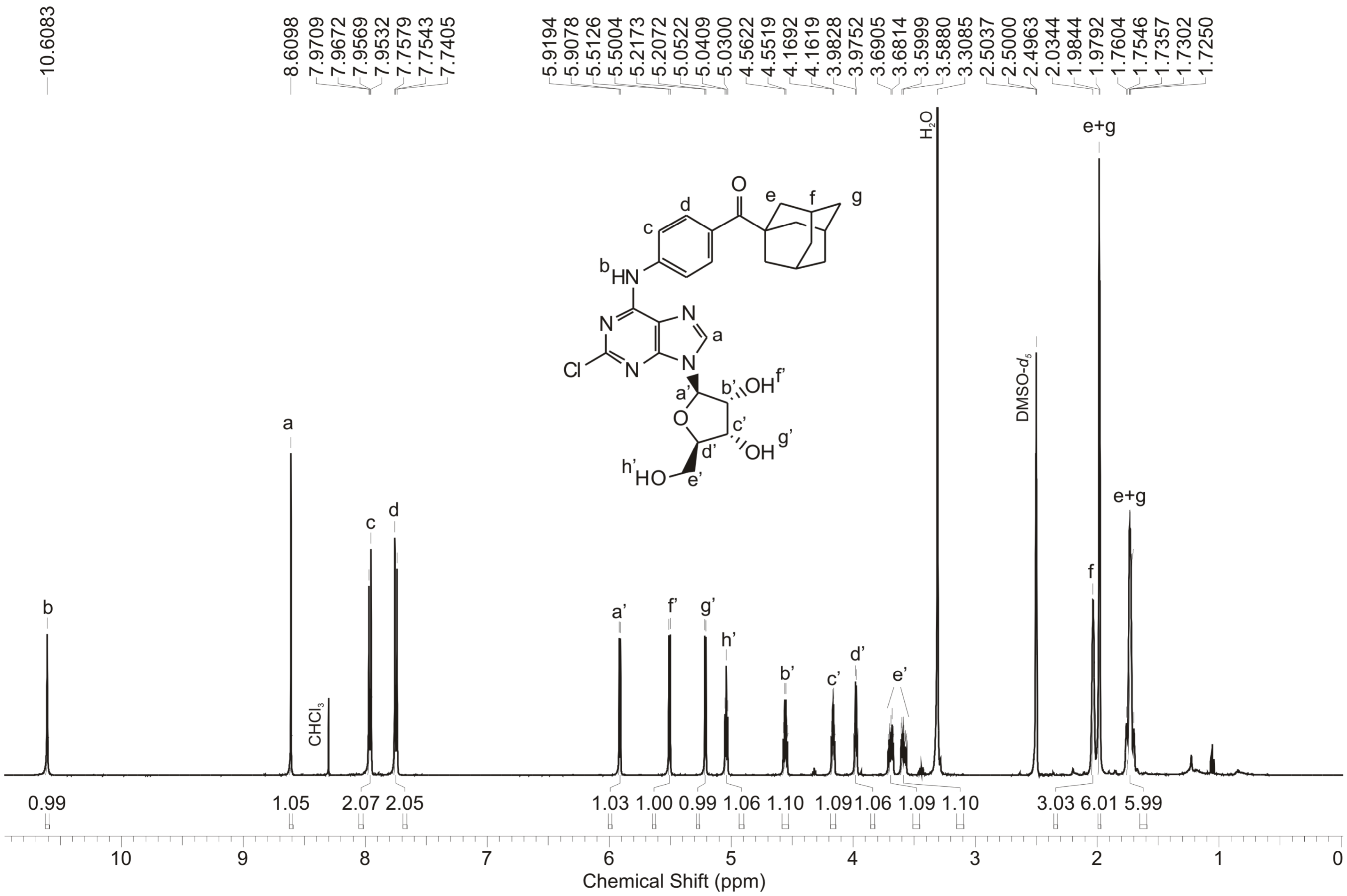

Figure S34. ^{1}H NMR spectrum (DMSO-d_6, 500 MHz, 303 K) of compound **20**.

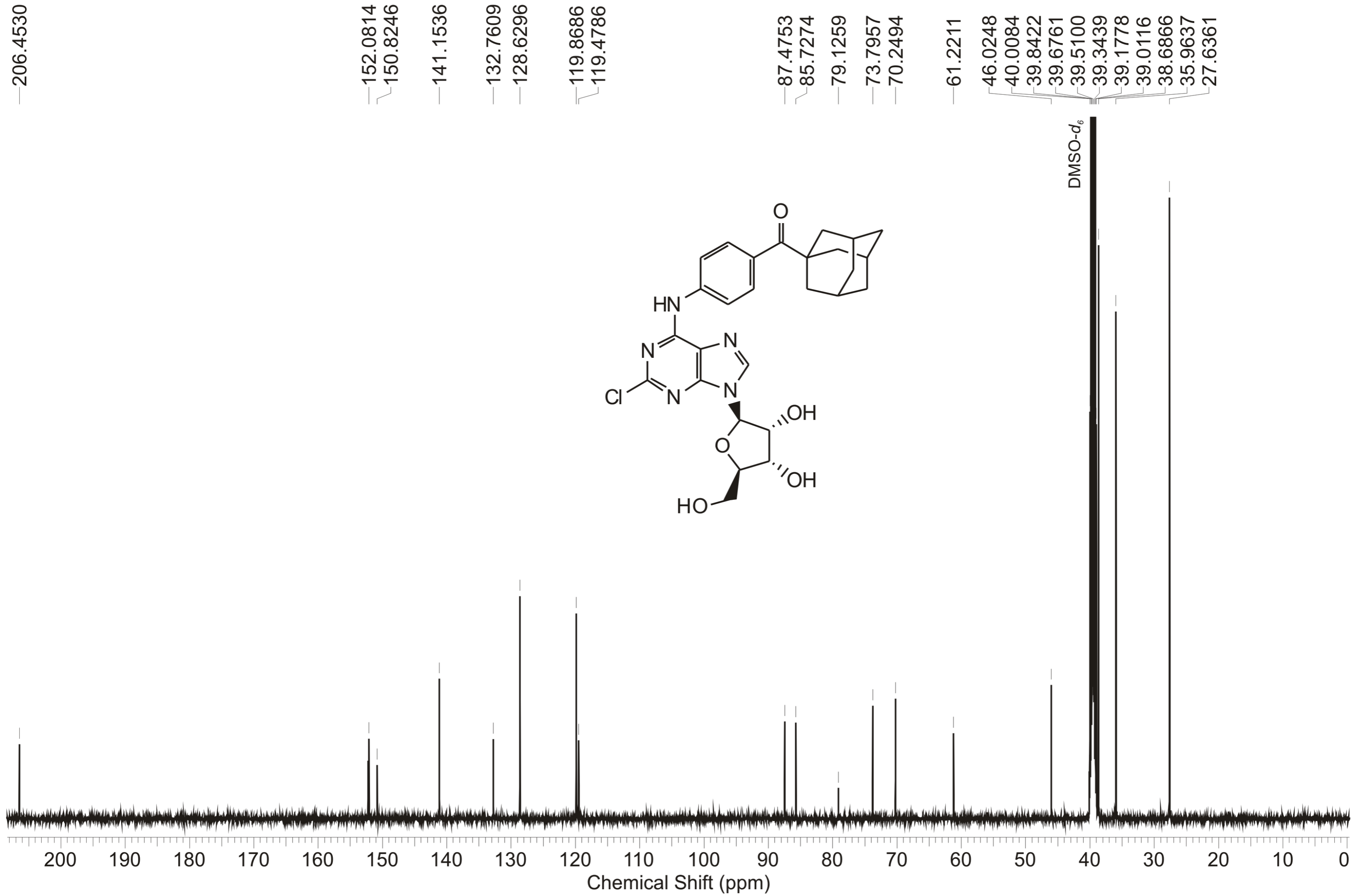

Figure S35. ^{13}C NMR spectrum (DMSO-d_6, 125 MHz, 303 K) of compound **20**.

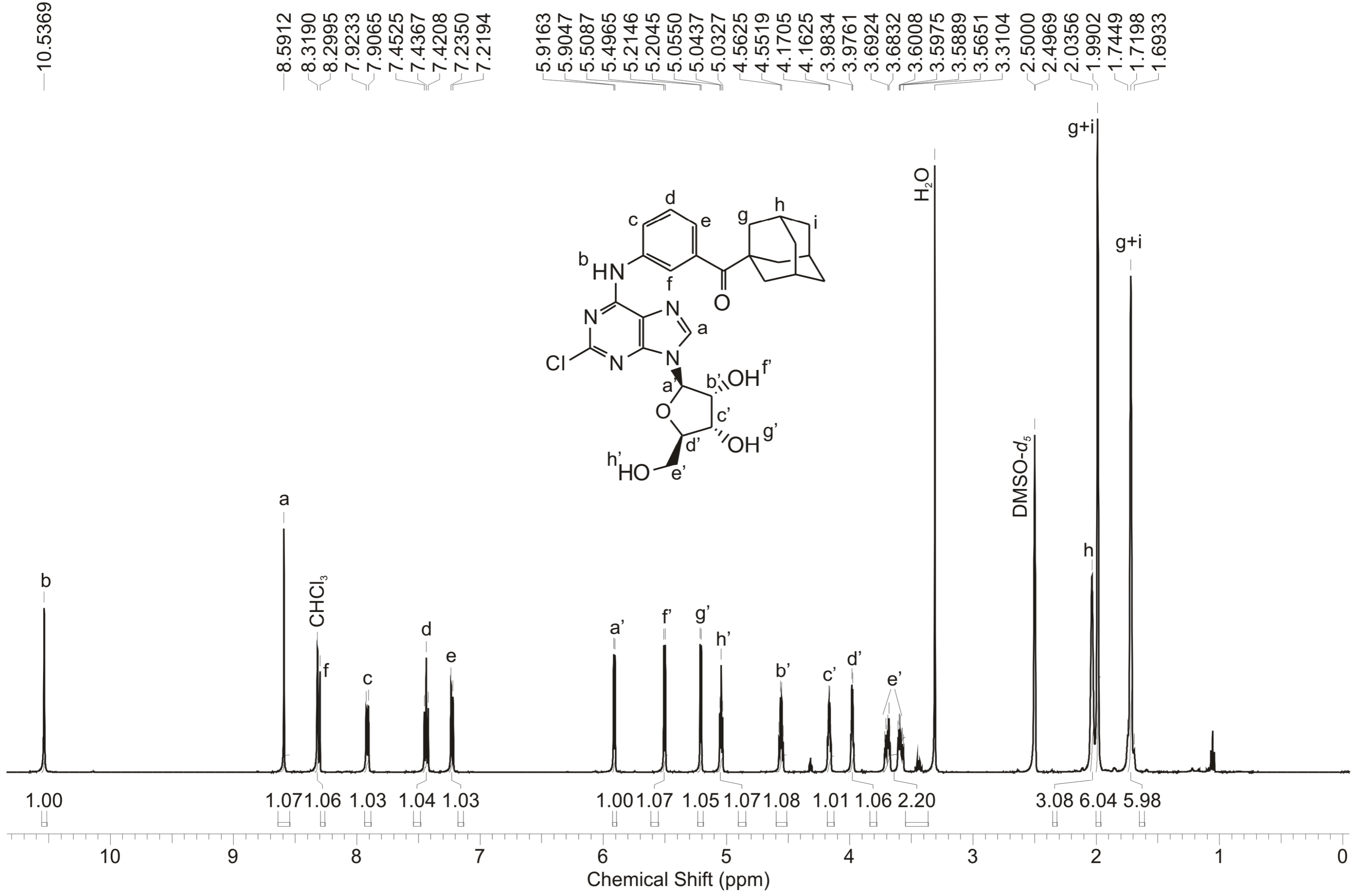

Figure S36. ^{1}H NMR spectrum (DMSO-d_6, 500 MHz, 303 K) of compound **21**.

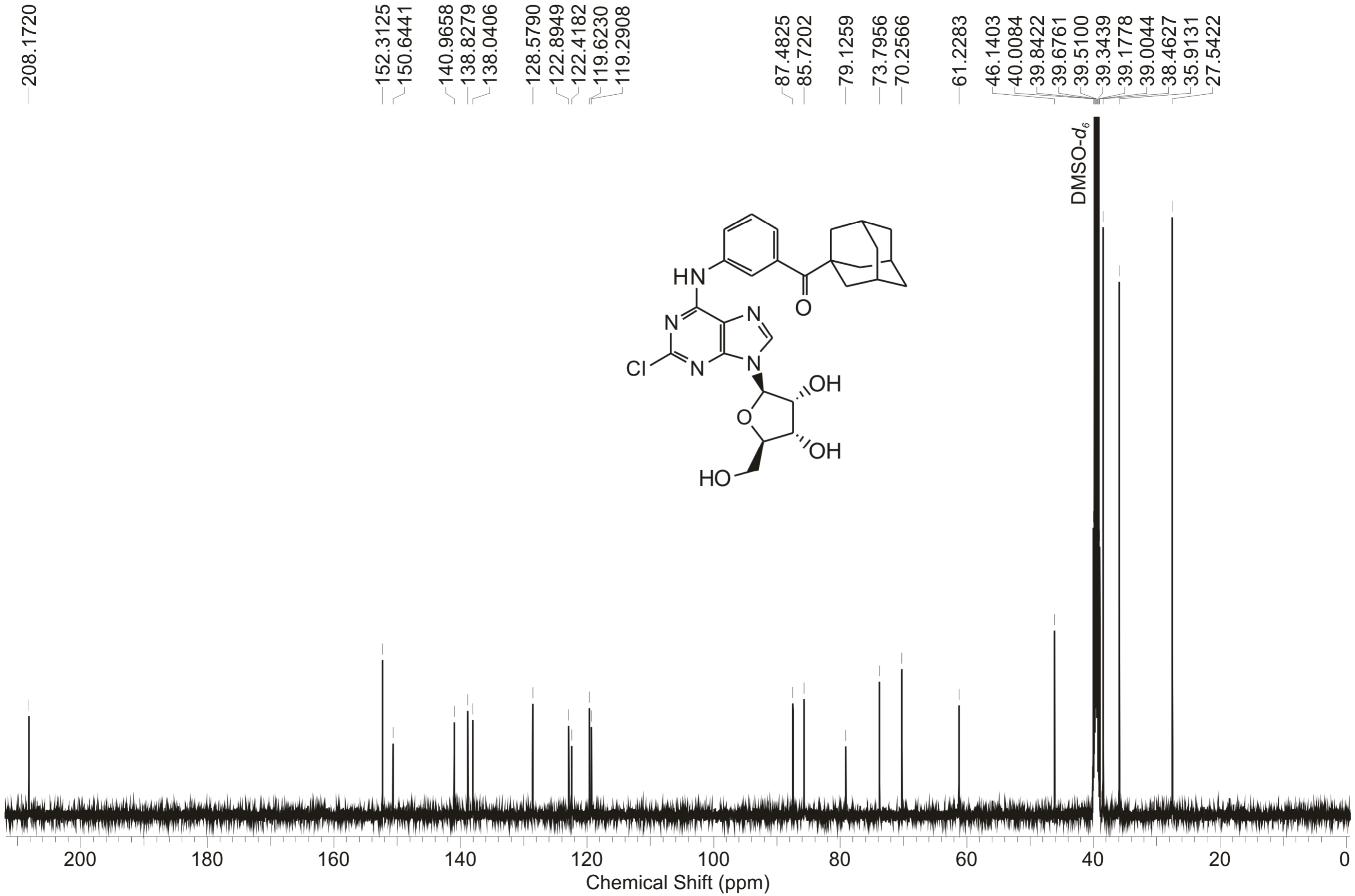

Figure S37. ^{13}C NMR spectrum (DMSO-d_6, 125 MHz, 303 K) of compound **21**.

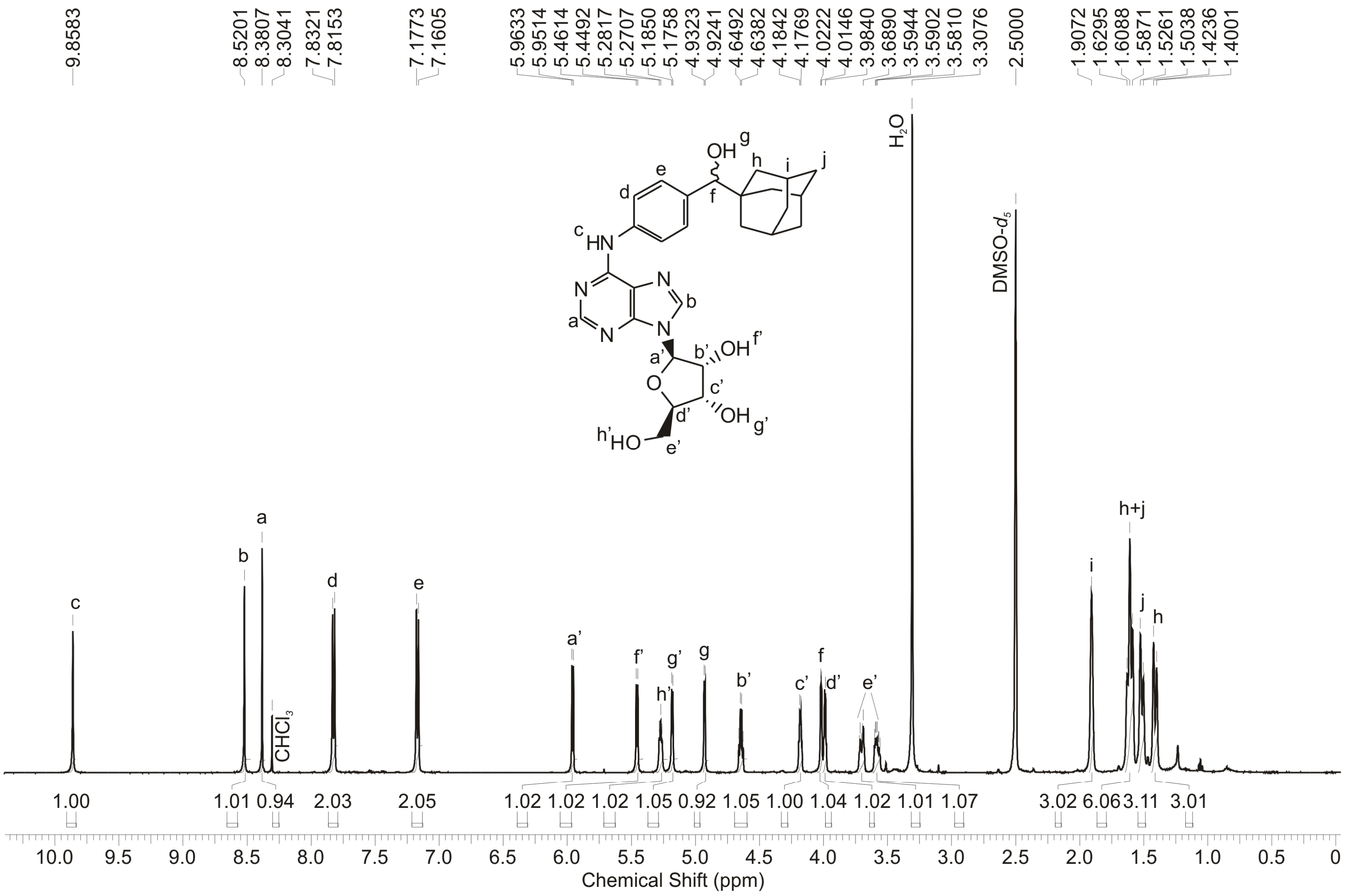

Figure S38. ^{1}H NMR spectrum (DMSO-d_6, 500 MHz, 303 K) of compound **22**.

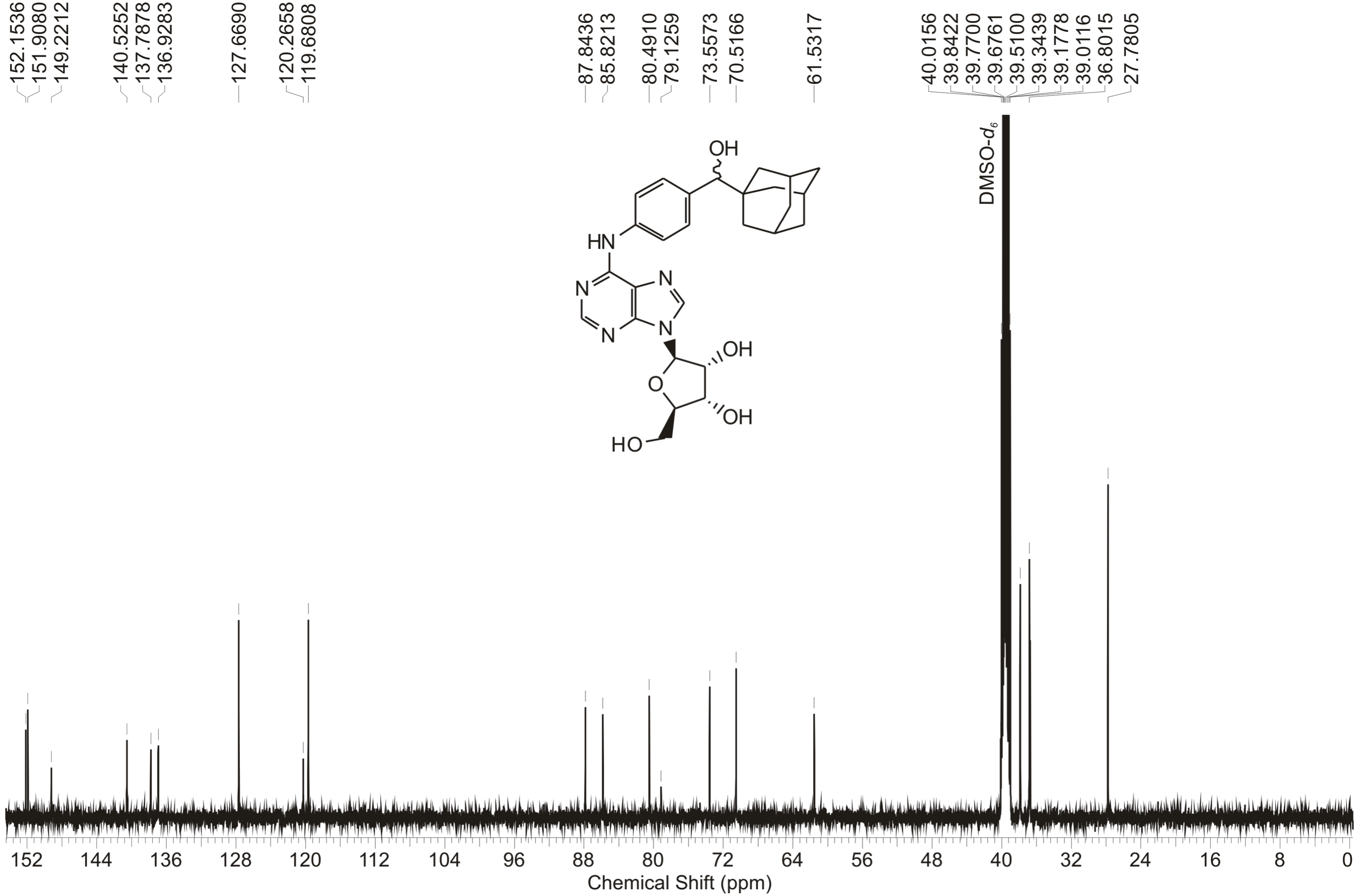

Figure S39. ^{13}C NMR spectrum (DMSO-d_6, 125 MHz, 303 K) of compound **22**.

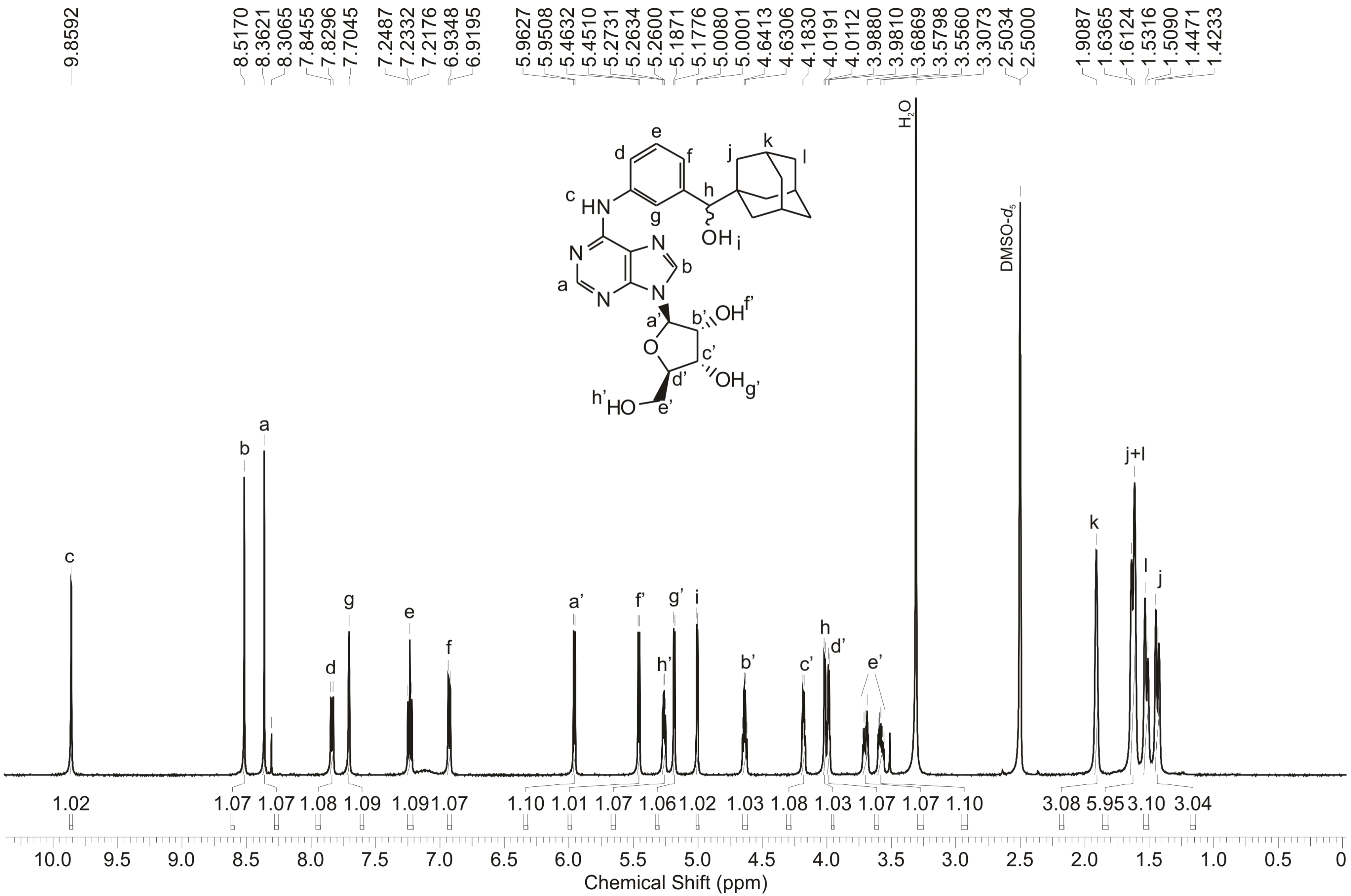

Figure S40. ^{1}H NMR spectrum (DMSO-d_6, 500 MHz, 303 K) of compound **23**.

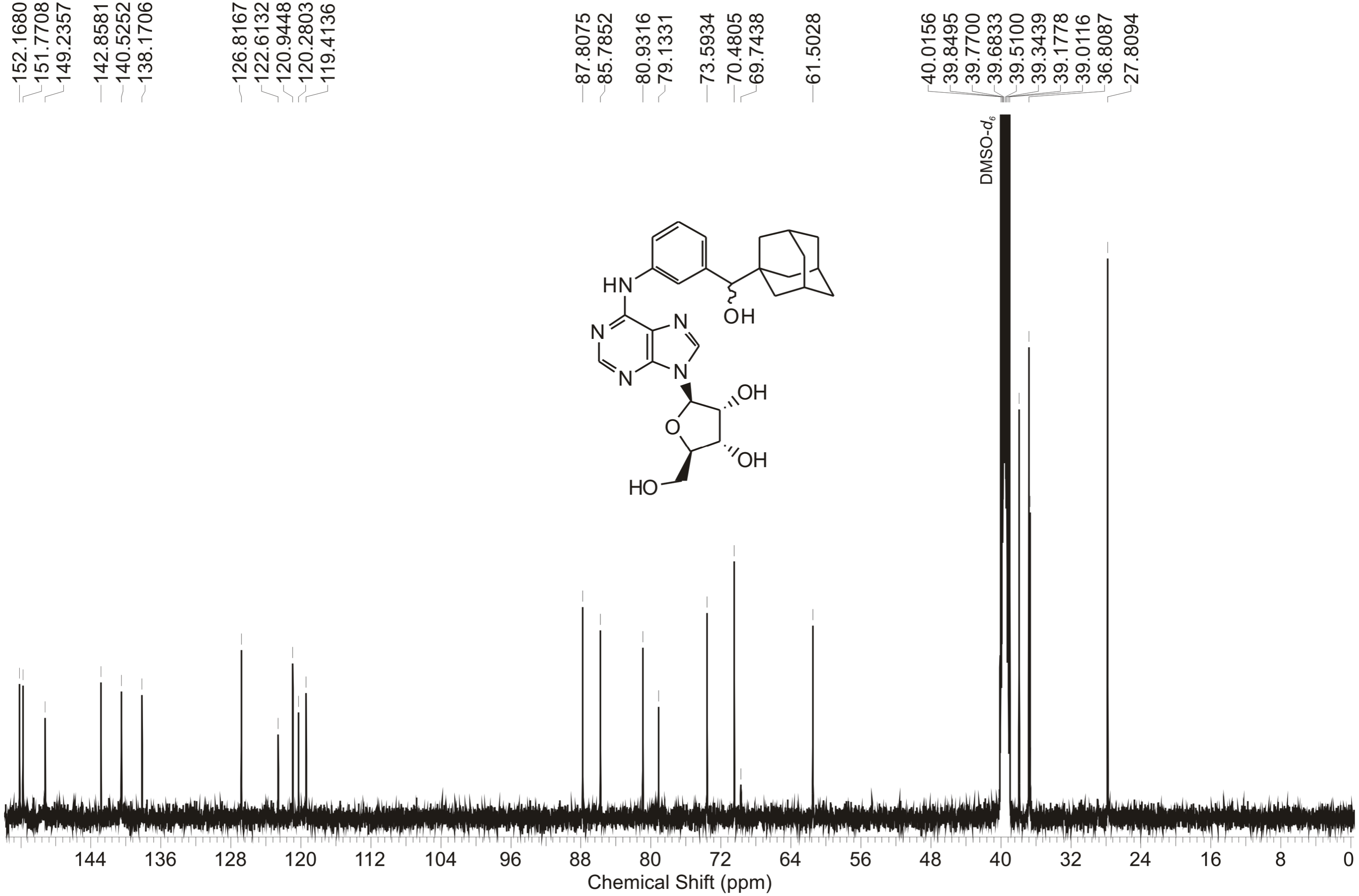

Figure S41. ^{13}C NMR spectrum (DMSO-d_6, 125 MHz, 303 K) of compound **23**.

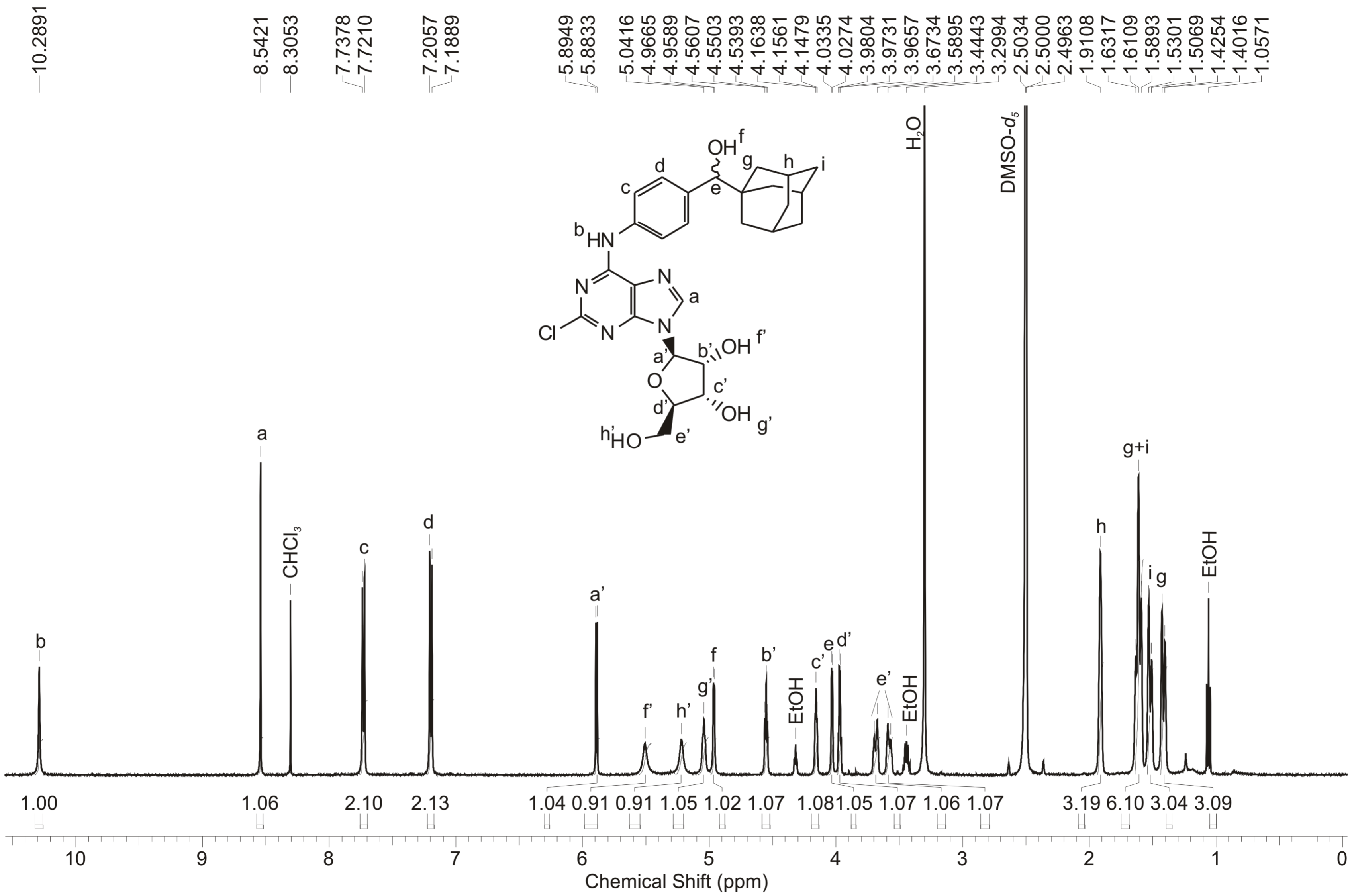

Figure S42. ^{1}H NMR spectrum (DMSO-d_6, 500 MHz, 303 K) of compound **24**.

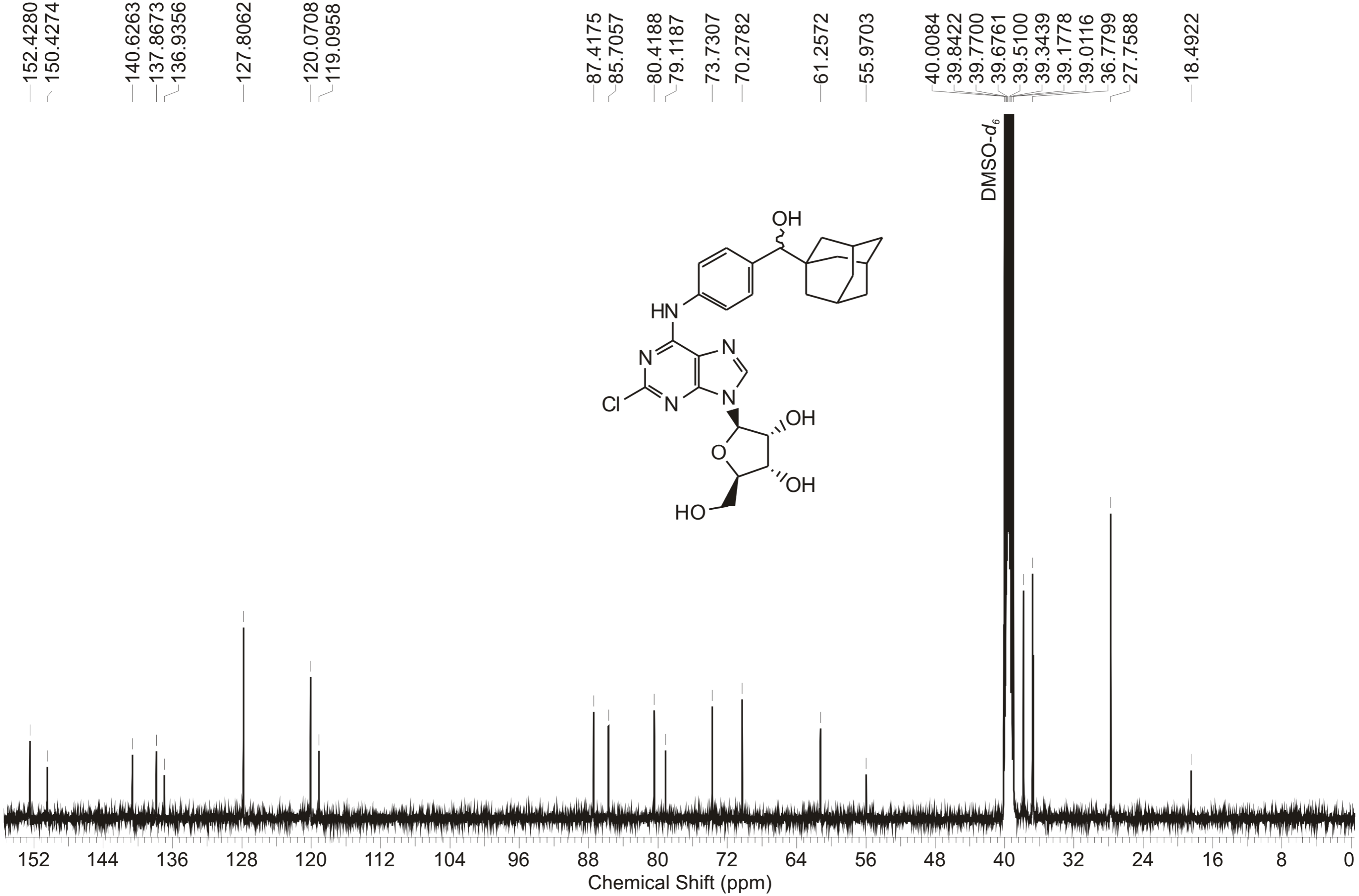

Figure S43. ^{13}C NMR spectrum (DMSO-d_6, 125 MHz, 303 K) of compound **24**.

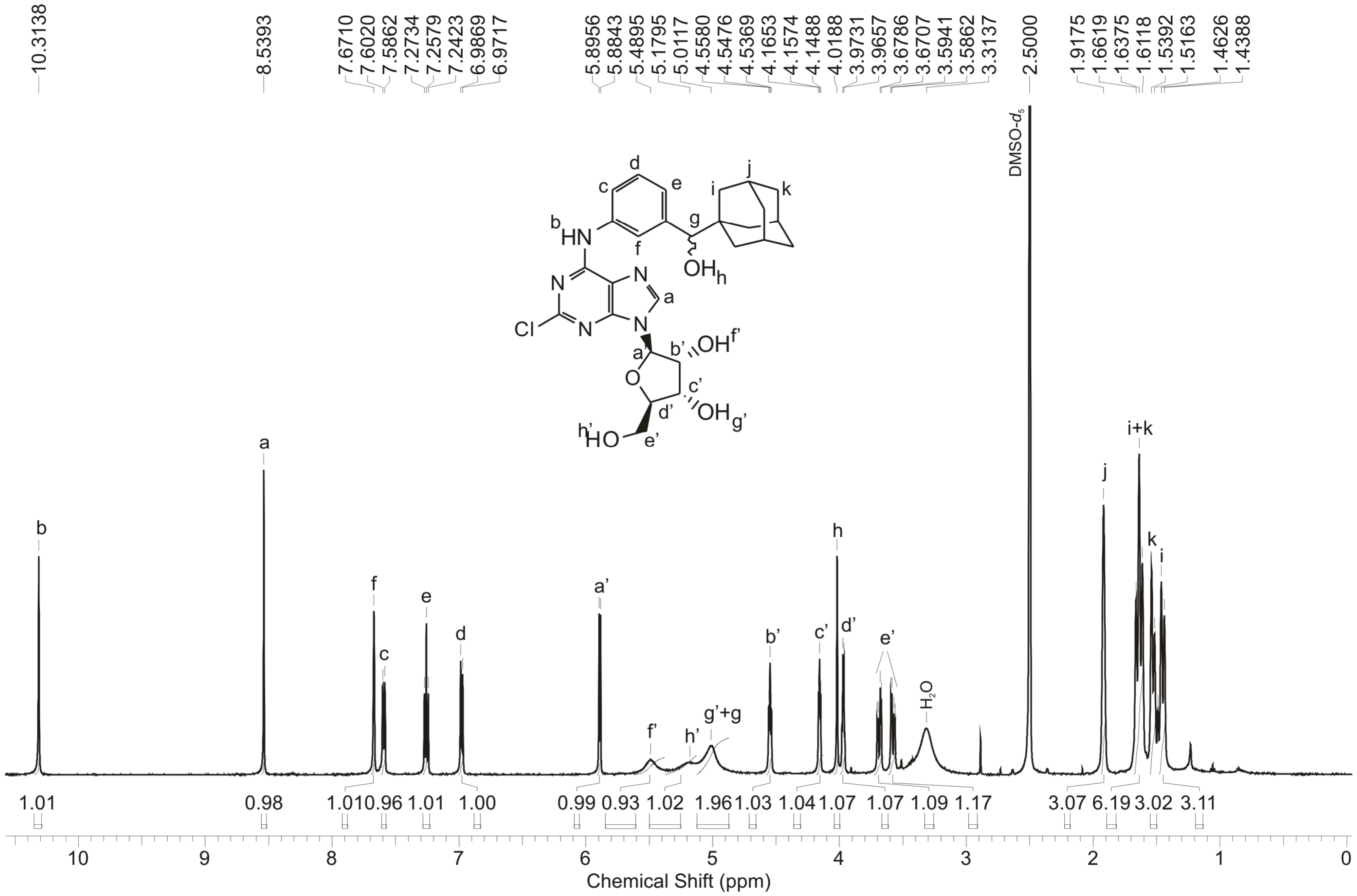

Figure S44. ^{1}H NMR spectrum (DMSO-d_6, 500 MHz, 303 K) of compound **25**.

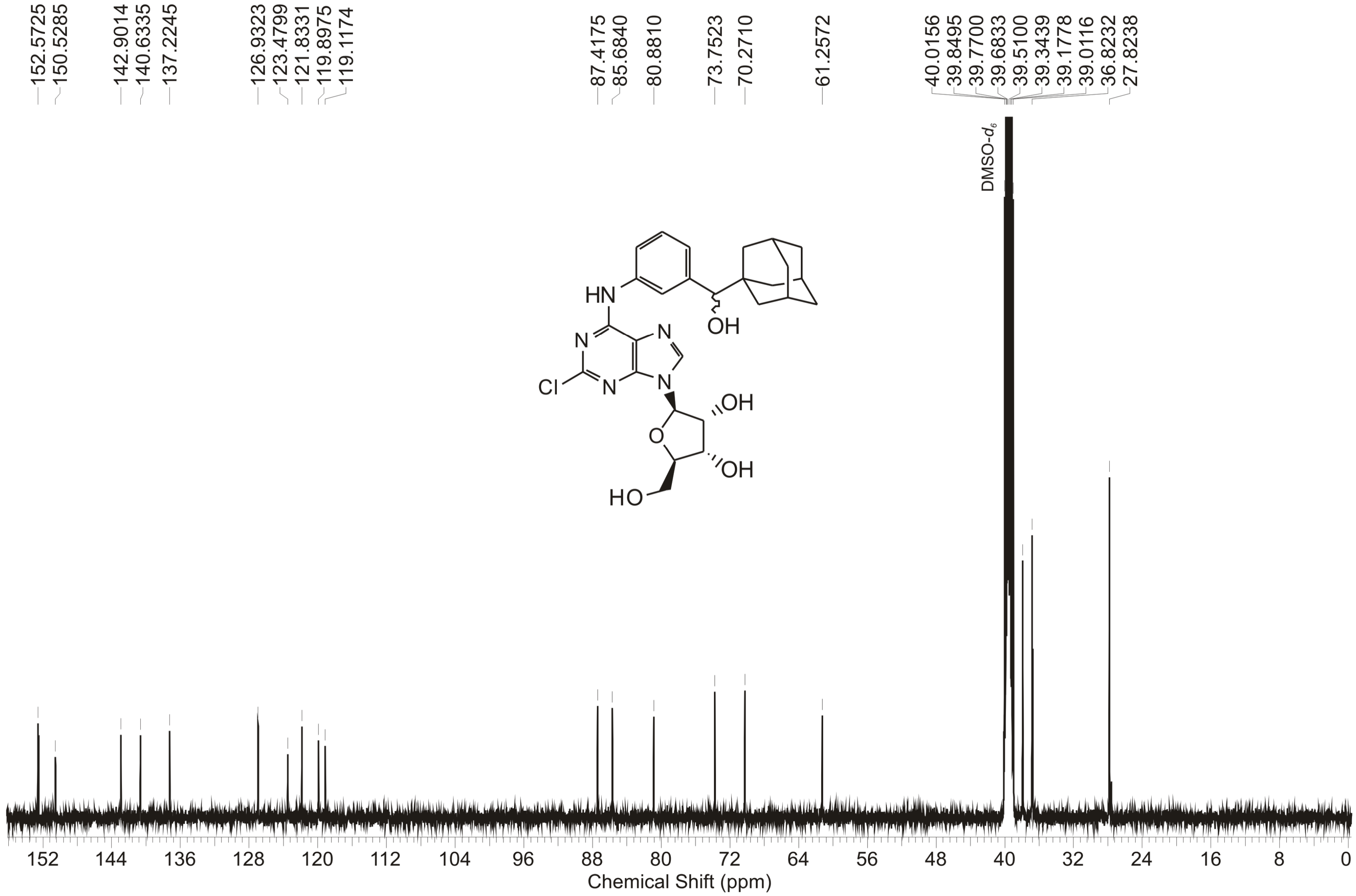

Figure S45. ^{13}C NMR spectrum (DMSO-d_6, 125 MHz, 303 K) of compound **25**.

Crystal data and structure refinement of compound 13

Table S1. Crystal data and structure refinement of compound **13**.

Compound	**13**
CCDC deposition number	2219330
Empirical formula	$C_{37}H_{28}ClN_5O_7$
Formula weight (g·mol^{-1})	690.09
Colour; shape	Colourless; block
Crystal size	0.20 × 0.20 × 0.15
Source; λ[Å]	Mo K/α; 0.71073
Measured temperature (K)	120 (2)
Crystal system	Orthorhombic
Space group	P 2_1 2_1 2_1
Unit cell dimensions (Å, °)	a = 10.3487(2)
	b = 13.0978(2)
	c = 24.0396(3)
	$\alpha = \beta = \gamma = 90$
Volume (Å^3)	3258.45(9)
Z	4
D_x (g·cm^{-3})	1.407
μ (mm^{-1})	0.178
Absorption correction	0.92073 to 1.00000
F(000)	1432
θ Range (°)	2.980 to 25.346
Completeness to θ (%)	0.998
h	$-11 \le h \le 12$
k	$-15 \le k \le 15$
l	$-28 \le l \le 28$
Reflections collected	19309
Reflections unique	5942 [R(int) = 0.0273]
Unique reflections with $I \ge 2\sigma(I)$	5636
Number of parameters	451
Goodness-of-fit on F^2	1.061
Final R indices [$I \ge 2\sigma(I)$]	R_1 = 0.0284, wR_2 =
R indices (all data)	R_1 = 0.0306, wR_2 =
Residual highest peak and deepest hole [illegible]	0.158 and −0.162

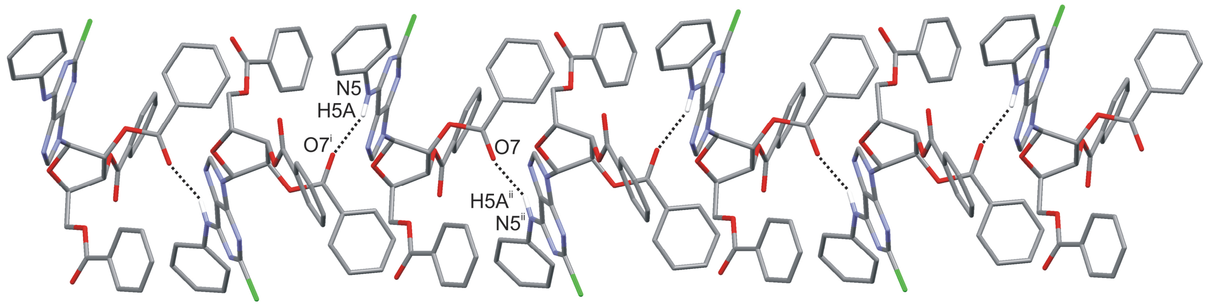

Figure S46. A crystal packing of compound **13**. Chains linked via N—H···O H-bonds are formed along the *b*-axis. H-atoms not participating in H-bonds are omitted for clarity. Symmetry: i 2−*x*, −0.5+*y*, 1.5−*z*; ii 2−*x*, 0.5+*y*, 1.5−*z*.
